# The dynamic role of nucleoprotein SHCBP1 in the cancer cell cycle and its potential as a synergistic target for DNA-damaging agents in cancer therapy

**DOI:** 10.1186/s12964-024-01513-0

**Published:** 2024-02-16

**Authors:** Mei Zhou, Limin Duan, Jiangbin Chen, Yumei Li, Zhengrong Yin, Siwei Song, Yaqi Cao, Ping Luo, Fan Hu, Guanghai Yang, Juanjuan Xu, Tingting Liao, Yang Jin

**Affiliations:** 1 Department of Respiratory and Critical Care Medicine, Hubei Province Clinical Research Center for Major Respiratory Diseases, NHC Key Laboratory of Pulmonary Diseases, Union Hospital, Tongji Medical College, Huazhong University of Science and Technology, Wuhan, Hubei 430030 China; 2 Hubei Province Key Laboratory of Biological Targeted Therapy, MOE Key Laboratory of Biological Targeted Therapy, Union Hospital, Tongji Medical College, Huazhong University of Science and Technology, Wuhan, Hubei 430022 China; 3 Hubei Province Engineering Research Center for Tumour-Targeted Biochemotherapy, Union HospitalTongji Medical CollegeHuazhong University of Science and Technology, Wuhan, Hubei 430022 China; 4 Department of Critical Care Medicine, Institute of Anesthesia and Critical Care Medicine, Union Hospital, Tongji Medical College, Huazhong University of Science and Technology, Wuhan, 430022 China; 5 Department of Translational Medicine Center, Union Hospital, Tongji Medical College, Huazhong University of Science and Technology, Wuhan, Hubei 430022 China; 6 Medical Subcenter of HUST Analytical & Testing Center, Tongji Medical College, Huazhong University of Science and Technology, Wuhan, Hubei 430022 China; 7 Department of Thoracic Surgery, Union Hospital, Tongji Medical College, Huazhong University of Science and Technology, Wuhan, Hubei 430022 China

**Keywords:** Tumour cell cycle, SHCBP1, DNA-damaging agents, Synergistic target

## Abstract

**Background:**

Malignant tumours seriously threaten human life and health, and effective treatments for cancer are still being explored. The ability of SHC SH2 domain-binding protein 1 (SHCBP1) to induce cell cycle disturbance and inhibit tumour growth has been increasingly studied, but its dynamic role in the tumour cell cycle and corresponding effects leading to mitotic catastrophe and DNA damage have rarely been studied.

**Results:**

In this paper, we found that the nucleoprotein SHCBP1 exhibits dynamic spatiotemporal expression during the tumour cell cycle, and SHCBP1 knockdown slowed cell cycle progression by inducing spindle disorder, as reflected by premature mitotic entry and multipolar spindle formation. This dysfunction was caused by G2/M checkpoint impairment mediated by downregulated WEE1 kinase and NEK7 (a member of the mammalian NIMA-related kinase family) expression and upregulated centromere/kinetochore protein Zeste White 10 (ZW10) expression. Moreover, both in vivo and in vitro experiments confirmed the significant inhibitory effects of SHCBP1 knockdown on tumour growth. Based on these findings, SHCBP1 knockdown in combination with low-dose DNA-damaging agents had synergistic tumouricidal effects on tumour cells. In response to this treatment, tumour cells were forced into the mitotic phase with considerable unrepaired DNA lesions, inducing mitotic catastrophe. These synergistic effects were attributed not only to the abrogation of the G2/M checkpoint and disrupted spindle function but also to the impairment of the DNA damage repair system, as demonstrated by mass spectrometry-based proteomic and western blotting analyses. Consistently, patients with low SHCBP1 expression in tumour tissue were more sensitive to radiotherapy. However, SHCBP1 knockdown combined with tubulin-toxic drugs weakened the killing effect of the drugs on tumour cells, which may guide the choice of chemotherapeutic agents in clinical practice.

**Conclusion:**

In summary, we elucidated the role of the nucleoprotein SHCBP1 in tumour cell cycle progression and described a novel mechanism by which SHCBP1 regulates tumour progression and through which targeting SHCBP1 increases sensitivity to DNA-damaging agent therapy, indicating its potential as a cancer treatment.

**Graphical Abstract:**

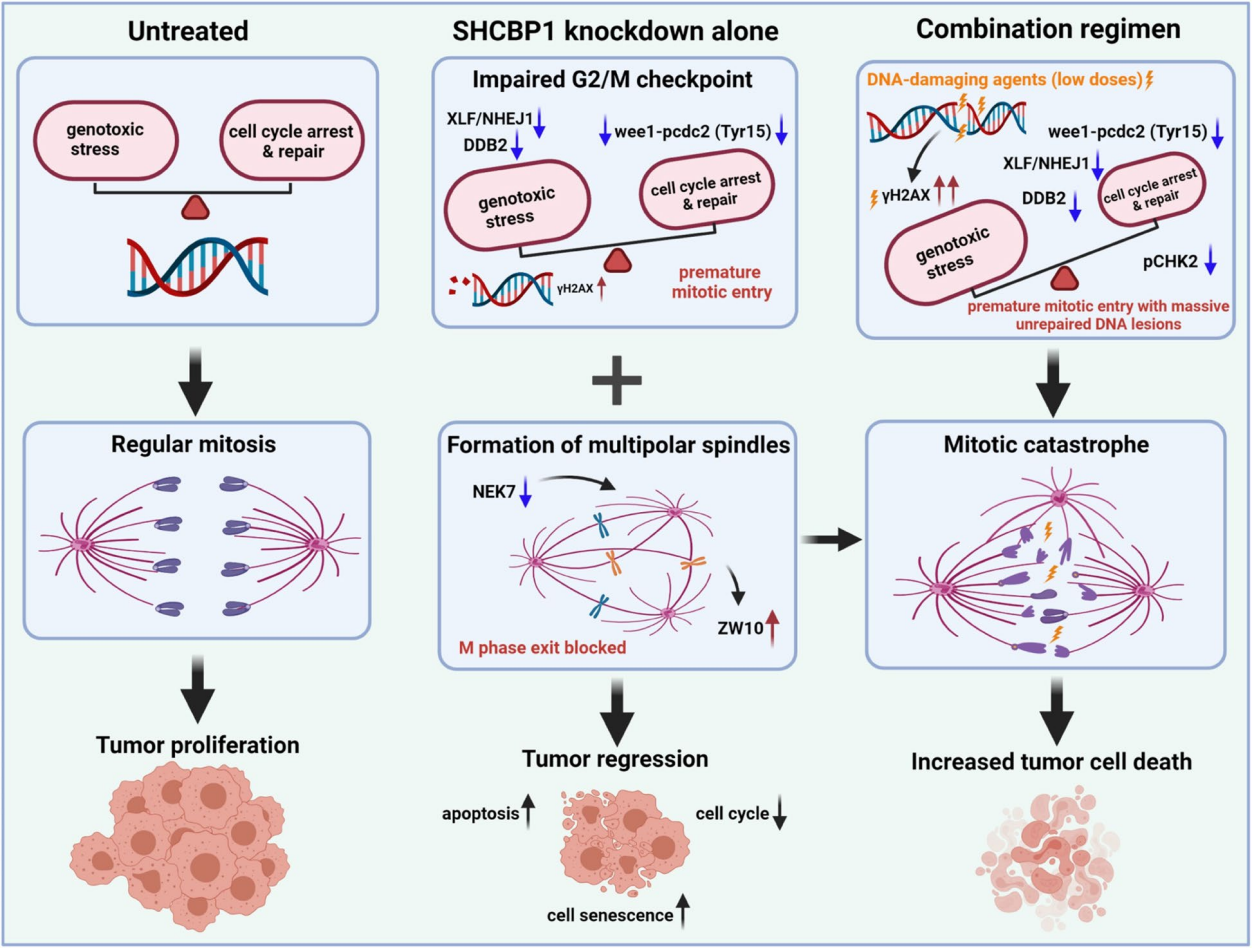

**Supplementary Information:**

The online version contains supplementary material available at 10.1186/s12964-024-01513-0.

## Introduction

The latest global cancer statistics reported approximately 19.3 million new cancer cases and 10 million cancer deaths in 2020 [1]. Lung cancer is the leading cause of cancer deaths worldwide. Non-small cell lung cancer (NSCLC) accounts for 85% of all lung cancer cases [2], with adenocarcinoma representing 50–60% of NSCLC cases [3]. Therefore, identifying potential targets to counteract tumour progression, especially that of lung adenocarcinoma (LUAD), is critical. Radiotherapy, platinum-based frontline chemotherapy, molecular targeted therapy, and immunotherapy are the main treatments for nonsurgical lung cancer [4]. However, the most advanced NSCLC progresses as a result of treatment resistance, and the overall mortality rate remains high. Therefore, novel effective therapeutic approaches for this aggressive malignancy are urgently needed.

The cell cycle is a highly controlled process, and the activity of cell cycle proteins in normal cells is strictly controlled by cell cycle-specific transcription, protein degradation, and related CDK inhibitor proteins [5], machinery that is often disrupted in most human cancers, leading to the progression of malignancy. This special nature of the tumour cell cycle provides opportunities for tumour-specific targeted therapies. While normal cells can repair endogenously or exogenously damaged DNA during G1 arrest, cancer cells often have a deficient G1–S checkpoint, thus depending on a functional G2–M checkpoint for DNA repair [6–8]. Therefore, synthetic therapeutic strategies targeting the G2–M checkpoint in the tumour cell cycle may yield favourable clinical outcomes with little toxicity to normal cells [6, 9–12]. DNA-damaging agents, including cytotoxic therapies and radiation, activate a cellular protection system that includes cell cycle arrest and DNA repair [13]. The G2–M checkpoint plays a key role in this process, allowing cancer cells time to repair DNA damage before mitosis, thereby preventing cell death caused by severe genotoxic stress. Hence, molecules targeting the G2–M transition may increase the efficacy of cancer therapy via DNA-damaging agents [6–8].

ShcSH2 domain-binding protein (SHCBP1, also known as mPAL) was first described by Schmandt et al. in a yeast two-hybrid screen of murine activated T-cell and embryo libraries [14], followed by reports of its pro-proliferative and development-related functions [15–19]. Although the potential cell cycle-related roles of SHCBP1 in promoting tumour proliferation, including LUAD, have been indicated in several studies [15, 20–23], the spatial–temporal expression and distribution of SHCBP1 in each phase of the tumour cell cycle and its related cell cycle functions have not been determined. In addition, whether targeting SHCBP1 could improve the anti-tumour effects of current chemotherapy strategies in NSCLC remains largely unknown. The results of the present study demonstrated that SHCBP1 is another tumour target molecule linked to cell cycle and DNA damage, We investigated the spatial–temporal changes in the nucleoprotein SHCBP1 throughout the tumour cell cycle and revealed that SHCBP1 knockdown induced formation of multipolar spindles because of dysregulated NEK7 and ZW10 expression, and forced premature mitotic entry by significantly down-regulating WEE1­phospho-cdc2 (Tyr15) axis, leading to a compromised G2–M checkpoint. Moreover, DNA damage induces the upregulation of SHCBP1 expression, and the antitumour effect is augmented by the combination of SHCBP1 inhibition with low-dose DNA-damaging agents, which induces tumour cells with unrepaired DNA damage, and especially those with P53 deficiency, to enter mitosis, triggering mitotic catastrophe.

## Materials and methods

### Human lung cancer samples

Specimens of tumour tissues and corresponding adjacent normal tissues (ANTs) from 213 patients with primary non-small cell lung cancer (NSCLC) who were referred for surgical resection to the Department of Thoracic Surgery, Wuhan Union Hospital, from Feb. 2012 to Dec. 2018 were collected for subsequent immunoblotting, real-time PCR and immunohistochemistry or immunofluorescence staining. The ANT tissues used in our study were taken from the surgical margin of the resected lung tissue. Pathologically, the surgical margins of all patients were proven to be tumouor free, and the margin distance (the shortest distance between the surgical margin and the tumour) was at least 2 cm or greater than the maximum tumour diameter. These patients were followed up once every six months after surgery, and the last follow-up was in December 2019. All patients received neither radiotherapy nor chemotherapy prior to surgery. Clinical information, such as age, sex, smoking history, and clinicopathologic characteristics, including TNM stage and EGFR mutation status, was collected. Malignant pleural effusion (MPE) specimens from 24 patients with lung adenocarcinoma (LUAD) who were referred to the Department of Respiratory and Critical Care Medicine, Wuhan Union Hospital, from June 2018 to October 2019 were collected. A tissue microarray (HLugA180Su02) from 93 patients with LUAD was purchased from Shanghai Outdo Biotech Co., Ltd.

All sample collections were performed with the understanding and written consent of each subject and in accordance with the Declaration of Helsinki. The study protocol was reviewed and approved by the Institutional Review Boards of the Medical Ethics Committee of Wuhan Union Hospital ([2010] IEC (S202) and [2017] IEC (S1006)).

### Cell lines and cell culture

All cell lines, including HBE, A549, NCI-H1299, NCI-H460, NCI-H292, HEK293T, HeLa and mouse-derived Lewis lung carcinoma (LLC) cell lines, were purchased from American Type Culture Collection (ATCC). The cells were maintained in RPMI 1640 or DMEM (Gibco, Carlsbad, CA, USA) supplemented with 10% FBS (Gibco, Carlsbad, CA, USA) and 1% penicillin/streptomycin in a 5% CO2 incubator at 37 °C. The stable luciferase (LUC)-expressing A549 (A549-LUC), HeLa (HeLa-LUC) and LLC (LLC-LUC) cells used in this study were infected with lentivirus packaged with a LUC overexpression vector (Ubi-MCS-Luc-IRES-puromycin). Cells were selected with puromycin (Sigma‒Aldrich, 1.5 μg/ml) for 7 days after lentiviral infection for 72 h, followed by puromycin (0.5 μg/ml) maintenance.

### Mouse models

Five- to six-week-old male C57BL/6 mice and female BALB/c nude mice were purchased from Changzhou Cavens Laboratory Animal Ltd. Mice were raised under specific pathogen-free conditions in the Experimental Animal Center of Tongji Medical College and allowed to adapt to housing in the animal facility for 1 week before the initiation of experiments. Mice were ear-tagged and randomly grouped prior to the experiment. The C57BL/6 mouse lung cancer metastasis model was generated via tail vein injection of LLC-LUC cells. Female BALB/c nude mice were used to establish a subcutaneous tumour model. Animals were photographed using bioluminescence (Goldbio Firefly D-Luciferin, potassium salt, #115144–35-9) under an in vivo optical imaging system (In Vivo FX PRO, Bruker Corporation) at the indicated times to track tumour growth and metastasis.

### Knockdown of SHCBP1 by RNA interference and lentiviral infection

In this study, SHCBP1 knockdown was accomplished by transfecting cancer cells with siRNAs against SHCBP1 via Lipofectamine™ RNAiMAX Transfection Reagent (Thermo Fisher Scientific, #13778150) following the manufacturer’s instructions. The sequences of the four different siRNAs used are listed in the key resources table of the Supplementary materials. siRNA3 and siRNA4 were used for verification. For in vivo or in vitro experiments requiring long-term observation, lentivirus-carrying SHCBP1 short hairpin (sh)RNAs were used for SHCBP1 knockdown. SHCBP1-shRNA was constructed into a hU6-MCS-CMV-EGFP plasmid stably expressing green fluorescent protein (GFP). The sequences of the shRNAs can be found in the key resources table of the Supplementary materials. The methods used for lentivirus packaging, infection and cell selection are described in the Supplementary materials.

### Immunohistochemical staining and scoring

The human NSCLC tissues from Wuhan Union Hospital and the purchased tissue microarray were subjected to immunohistochemical (IHC) staining for SHCBP1 and Ki67 following the standard protocol, which was performed with horseradish peroxidase (HRP) conjugates and diaminobenzidine (DAB) detection. IHC images were acquired by a Nikon Biological Microscope (Ni-E, Japan), and the IHC staining of SHCBP1 and Ki67 was scored by two observers in a blinded manner (see details in the Supplementary methods). The IHC staining scores were used for subsequent survival and correlation analyses.

### Immunofluorescence staining

Lung adenocarcinoma tissue was paraffin-embedded and sectioned, and cells were processed as previously indicated. Then, the tissue and cells were subjected to immunofluorescence staining for SHCBP1, α-tubulin, γ-tubulin, γH2AX, phosphorylated histone H3 (Ser10), PLK1, RACGAP1, MKLP1, and NPM1. Tissue processing was the same as that for IHC staining described previously, except that secondary antibodies conjugated with Alexa Fluor were used. Cells growing in 12- or 24-well plates with sterile glass slides were washed with PBS, fixed in 4% PFA for 15 min at room temperature, permeabilized with PBS containing 0.5% Triton-X100 and blocked in PBS containing 3% BSA. While protected from light, the sections were incubated with primary antibodies overnight at 4 °C and then with Alexa Fluor–conjugated secondary antibodies for 1 h at 37 °C. Hoechst was used for nuclear staining. High-resolution, 3D confocal imaging of the slides was performed with an LSM 780 laser scanning confocal microscope (Carl Zeiss, AG, Germany).

### Preparation of cytoplasmic and nuclear fractions

The treated cells were washed and scraped with cold PBS. A portion of the cells was directly lysed to extract total protein (T) and stored at –80 °C. The other samples were then centrifuged and resuspended in cytoplasmic extraction buffer. The cells were then properly homogenized and centrifuged at 4000 rpm for 5 min (4 °C). The supernatant was centrifuged again at 13000 rpm for 15 min (4 °C) to obtain the second supernatant (cytoplasmic fraction), which was stored at –80 °C until use. The nuclei in the pellet were isolated by gradient centrifugation with sucrose solution and resuspended in nuclear extraction buffer for 20 min at 4 °C. After sonication and centrifugation at 15,000 × g for 15 min at 4 °C, the supernatant (nuclear fraction) was stored at –80 °C. Finally, the total, cytoplasmic and nuclear fractions were subjected to western blot analysis. GAPDH and Lamin B1 served as loading controls for the cytoplasmic and nuclear fractions, respectively.

### Coimmunoprecipitation

To detect endogenous interactions between SHCBP1, RACGAP1, MKLP1 and PLK1, tumour cells growing in 10 cm dishes were lysed in 1 ml of NP-40 buffer supplemented with PMSF and complete protease inhibitor cocktail. Following centrifugation at 12,000 × g at 4 °C for 5 min, the supernatants were collected, and the protein concentrations were determined using a BCA protein quantification kit. Lysates containing 0.5–2 mg of protein were subjected to IP (depending on the protein abundance), and some lysates were stored at –80 °C as immunoblotting controls. Briefly, lysates (1 mg protein) were diluted with NP-40 buffer to 500–800 μl and incubated with 30 μl protein A/G agarose beads and 5 μg primary antibody overnight at 4 °C with vertical rotation. Then, the immunoprecipitates were washed and resuspended in Tris-SDS buffer, boiled at 95 °C for 5 min, and chilled on ice, followed by centrifugation at 16,200 × g for 5 min. The supernatants were then mixed with β-mercaptoethanol and bromophenol blue for subsequent western blotting.

### Western blotting

Frozen tissue specimens were ground under liquid nitrogen for protein extraction. Cells were plated on 12-well or 6-well plates and treated as indicated. Whole-cell extracts were prepared using western lysis buffer supplemented with PMSF and complete protease inhibitor cocktail. The protein concentration was determined by the BCA method as described above. Lysates containing 5–30 µg of protein (mixed with β-mercaptoethanol and bromophenol blue) were loaded onto SDS‒PAGE gels and transferred onto nitrocellulose membranes. The membranes were blocked in 3% w/v milk and then incubated with the specific diluted primary antibody overnight at 4 °C. The membranes were washed and then incubated with a 1:10,000 dilution of the secondary antibody (IRDye® 800CW anti-rabbit or mouse IgG). The membranes were again washed in TBST, and the signals were visualized and analysed using Odyssey CLX (LI-COR, USA). The membranes were stripped using Restore™ Western Blot Stripping Buffer (Thermo Fisher) for sequential detection.

### RNA extraction and real-time polymerase chain reaction (RT–qPCR)

Total RNA was extracted from frozen tissue specimens and cells with TRIzol buffer using a standard RNA extraction protocol. The RNA was then reverse transcribed to cDNA using a cDNA synthesis kit (TOYOBO). Finally, the mRNA levels were quantified in triplicate using a real-time PCR system (CFX Connect Real-Time PCR Detection System, Bio-Rad) with a SYBR-Green-based All-in One qPCR RT Kit (Genecopoeia). The changes in the expression of the target genes relative to the expression of the housekeeping gene control were determined by the 2^−^ΔΔCt method. All the primer sequences used are provided in the supplementary materials (Table S4).

### Cell cycle synchronization

For cell synchronization, cells were synchronized to the G0 phase in serum-free medium, to the G1/S phase by double thymidine blockade, to the late G2 phase by RO3306 arrest and to the early M phase by nocodazole treatment. Briefly, cells were synchronized to the G0 phase by serum deprivation with a culture medium containing 0.2% FBS for 16–18 h. To synchronize cells to the G1/S phase, 2.5 mM thymidine (at 50–70% confluency) was added to the culture for 16 h, after which the cells were released for 8 h and then incubated again in thymidine for 16 h. Cells were arrested in the late G2 phase by adding 9 μM RO3306 for 20–24 h and in the early M phase after treatment with 0.2–0.4 μM nocodazole for 12–16 h. Cells were either collected for protein-related analyses or released from the above arrest for subsequent assays.

### DNA damage attack in G2 phase

Cells were synchronized in S phase by double thymidine treatment, followed by a 5–8 h release (depending on the doubling times of different cell lines) to allow progression into G2 phase, and pulsed with 15 μM etoposide for 1.5 h to induce robust DNA damage in G2 phase before washing and releasing into fresh medium (with or without 0.2 μM nocodazole).

### Flow cytometry (FCM) staining and cell cycle analysis

For the FCM analysis of apoptosis, 1.0–5.0 × 10^6^ cultured cells were collected by trypsin digestion without EDTA and washed twice by cold PBS. After centrifuged at 800 rpm for 5 min, cells were resuspended in 200 μL 1xAnnexin V Binding Buffer (BD Pharmingen) and stained under dark with 2 μl APC -conjugated Annexin V antibody (BD Pharmingen) for 30 min. After being washed twice, cells were resuspended in 200 μL of 1xAnnexin V Binding Buffer again and added with 5 μL 7-AAD to incubate for 15–20 min, which were finally examined by BD LSRFortessa X-20 flow cytometer (San Diego,CA).

For the FCM analysis of the cell cycle, trypsinized cells were fixed overnight in 70% ethanol at -20 °C, washed with staining buffer, and stained with an anti-phospho-histone H3 (PHH3) antibody (Alexa Fluor® 488 Conjugate; Cell Signaling Technology, 1:100) at 4 °C for 30 min. DNA was subsequently stained with 40 μg/ml propidium iodide (PI) solution for 15–20 min. The cells were finally examined by flow cytometry. All of the acquired flow cytometry data were analysed with FlowJo 10.0 or ModFit LT5.0 software. To dynamically study the process of tumour cell cycle progression, we blocked A549, NCI-H1299 and HeLa cells at the G1/S or G2/M phase by double thymidine or RO3306 blockade as previously mentioned, followed by release into fresh medium for various time periods and subsequent cell cycle analysis.

### Click-EdU pulse chase flow cytometry assay

A549 cells transfected with siCtrl or siSHCBP1 were incubated with 10 µM EdU (Click-iT™ EdU Alexa Fluor™ 647 kit, Thermo Fisher Scientific, Cat# C10424) for 45 min, after which the medium was removed and replaced with new medium containing 20 µM thymidine for 0, 2, 4, 6, 8, 10, 12, 14, 16, or 18 h. The cells were subsequently harvested for EdU and PI staining according to the manufacturer's instructions, after which FCM was performed. Finally, fractions of cells in the EdU-negative G1-phase compartment, EdU-negative G2/M phase compartment, and fraction of EdU positive cells that have divided were analyzed [24–27].

### Cell proliferation assays

Cell growth was estimated by live cell imaging (Celigo imaging cytometer, Nexcelcom), a Cell Counting Kit-8 (CCK8) assay, or an EdU incorporation assay (Thermo Fisher Scientific) according to the manufacturer’s instructions. Tumour cells were infected with shCtrl or shSHCBP1 lentivirus (expressing GFP) for 48–72 h or otherwise indicated and then subjected to the above assays. The specific details and procedures can be found in the Supplementary materials.

### Colony formation assay

In triplicate, cells infected with shCtrl or shSHCBP1 lentivirus for 72 h were seeded in 6-well plates at 500–1500 cells per well containing 1.5 ml of medium. After culturing for 10–14 days in a 37 °C, 5% CO2 incubator, the cells were fixed with 4% paraformaldehyde, stained with crystal violet dye, and then observed under a Nikon Biological Microscope (Japan).

### β-galactosidase staining

To investigate cell senescence, β-galactosidase (SA-β-Gal) staining was performed using a commercial kit (Cell Signaling Technology) following the manufacturer’s instructions. Briefly, cells were cultured to ~ 60% confluence in 12-well plates, transfected with control or SHCBP1 siRNA for 24–36 h and subsequently treated with low-dose etoposide (1 μM for A549 cells and 3 μM for HeLa cells) or the corresponding vehicle for another 24 h. The cells were fixed with 1 × fixative solution at room temperature for 15–20 min, washed with PBS three times and incubated with β-galactosidase staining solution (pH 6.0) overnight at 37 °C in a shaking incubator in the dark. Senescent cells produced a green fluorescent product and were observed and photographed under a Nikon biomicroscope (Ni-E, Japan).

### Proteomic analysis

For sample preparation, four sets of cell samples were prepared, each with two biological replicates. Briefly, NCI-H1299 cells were transfected with control or SHCBP1 siRNA for 24 h and then exposed to 5 μM etoposide or the corresponding vehicle (DMSO) for 24 h. The cells were washed and scraped off in 500 μl cold PBS on ice and subsequently lysed and ultrasonicated. After the proteins were digested with trypsin, the peptides were desalted on a Strata X C18 SPE column (Phenomenex), vacuum-dried, labelled with a tandem mass tag (TMT), and separated according to the instructions for the LC‐MS/MS system (see details in the Supplementary methods). The resulting MS/MS data were processed using the MaxQuant search engine (v.1.5.2.8). Tandem mass spectra were searched against the human UniProt database concatenated with the reverse decoy database. The search parameters were set to the modified default values.

The protein concentration was quantified by taking the median of the corresponding specific peptide quantification values. For each replicate experiment, the ratio of protein quantification values between two different samples (fold change) was considered the differential expression of the comparison group. For each comparison, we used the average fold change (FC) and coefficient of variation (CV) of the fold change (FC) values of two repeated experiments to identify the differentially expressed proteins (DEPs) for which the CV value was < 0.2 and the FC was > 1.25 or < 0.80. The MS information and analysis between each comparison group are listed in the Supplementary Materials.

The proteins were functionally annotated by GO analysis into three categories: biological process (BP), cellular compartment (CC) and molecular function (MF). The Encyclopedia of Genes and Genomes (KEGG) database was used to identify enriched pathways. Two-tailed Fisher’s exact tests were used to test the enrichment of the DEPs against all identified proteins. A corrected *p* value < 0.05 was considered to indicate statistical significance. To identify evolutionary homologues (orthologous and paralogous) of DEPs, we performed euKaryotic Conserved Orthologous Groups (KOG) analysis. Furthermore, to cluster DEPs according to the expression patterns of the four groups of samples that received different treatments, we applied fuzzy c-means clustering (k = 6, m = 2) to all DEPs by using the R package Mfuzz.

### Mouse experiment

A total of 1 x 10^6^ LLC-LUC cells (LLCs with stable luciferase expression) infected with shCtrl or shSHCBP1 lentivirus for 4–5 days were injected into 6–7-week-old C57BL/6 mice through tail vein to establish the mouse lung metastasis model. The mice were weighed every other day and their hair, breathing and activity were observed. The first in vivo imaging was performed to observe systemic metastasis of lung cancer cells in mice on day 25 after inoculation with LLC-LUC cells. The second in vivo imaging was performed on the 30th day and the surviving mice were sacrificed. At the same time, the lung tissues were removed for formaldehyde fixation and photographed.

To establish xenograft tumour model, female BALB/c nude mice aged 6 to 7 weeks were randomly divided into two groups (shCtrl vs. shSHCBP1). 5 × 10^6^ A549-LUC cells (A549 cells with stable luciferase expression) infected with shCtrl or shSHCBP1 lentivirus for 4 to 5 days were subcutaneously inoculated in the right hind limb of each mouse in two groups. Subcutaneous tumour size and the body weight of the mice were monitored every other day, tumour volume was measured with a caliper and calculated using the formula V (cm3) = (length x width^2^)/2. Mice were subjected to in vivo imaging using bioluminescence and sacrificed 23 days after tumour inoculation.

To identify the combination effects in vivo, 5 x 10^5^ HeLa-LUC cells (Hela cells with stable luciferase expression) infected with shCtrl or shSHCBP1 lentivirus for 4–5 days were subcutaneously inoculated in the bilateral inguinal region of BALB/c nude mice, respectively. The tumour-bearing mice were given low-dose etoposide (15 mg/kg) or the normal saline (NS) through intraperitoneal injection every other day when the subcutaneous tumour reaching around 200 cm^3^, and the mice were sacrificed 31 days after tumour inoculation (study end point). Mice underwent in vivo imaging before the first etoposide (day 21) administration and after the last etoposide administration (day 31). At the study end point, all tumour specimens will be randomly selected to detect the knockdown efficiency of SHCBP1 through the RT-PCR and western blotting analysis. All animal experimental protocols in this study were approved by the Animal Care Committee of Tongji Medical College ([2017] IACUC: S849) and complied with AALAC and NIH animal care guidelines.

### Statistical analysis

All data were shown as mean ± standard deviation (SD) or n (%). The cutoff for gene expression in public database was defined by average values. Student’s t test (paired or unpaired), one-way analysis of variance (ANOVA) with multiple comparisons, and χ2 analysis were used to compare the difference in cancer cells, tissue samples or clinical data. Animal data and the cell proportion change curve of each cell cycle phase were analyzed by Two-Way ANOVA. Log-rank test was used to assess survival significance. All statistical tests used are noted in figure legends and performed using GraphPad Prism 7.0 software (GraphPad) or SPSS (version 26). A two-tailed *p* value < 0.05 was considered statistically significant and was represented by *, *p* < 0.05; **, *p* < 0.01; ***, *p* < 0.001; ****, *p* < 0.0001.

## Results

### High SHCBP1 levels indicates poor survival outcomes and low sensitivity to DNA-damaging treatment in NSCLC patients

**Fig. 1:**
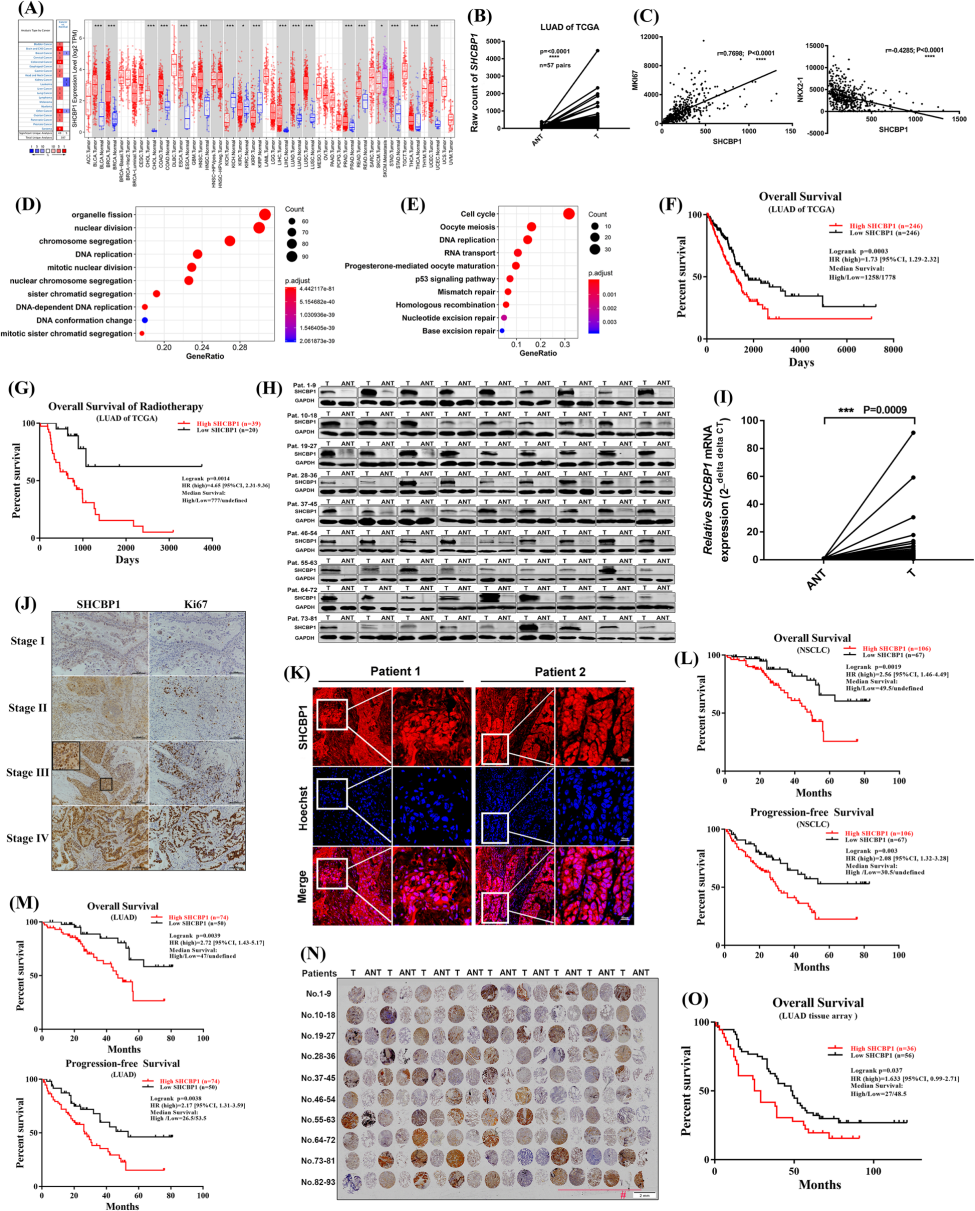
High expression of SHCBP1 in LUAD tissues was associated with poor prognosis. **A** SHCBP1 expression in paired tumour and normal tissues in pan-cancer data of TCGA. The Oncomine dataset (left panel) represents the expression of SHCBP1 in the existing pan-cancer database. The selected criteria were fold change > 2 and *p*-value < 0.0001. The SHCBP1expression in 37 kinds of tumour types with or without adjacent normal tissues determined by TIMER from TCGA (right panel). Statistical significance is represented as *, *P* < 0.05; **, *P* < 0.01; ***, *P* < 0.001. Boxplots indicate median, lower and upper quartile. **B** SHCBP1 mRNA expression in paired LUAD and normal tissues (*n* = 57) from the TCGA database. Paired t-test was used for the analysis; ****, *P* < 0.0001. **C** Correlation analysis of mRNA expression level between SHCBP1 and MKI67 or NKX2-1 in LUAD tissues from TCGA database (*n* = 504). Pearson correlation analysis provides correlation coefficient (r) and *P* -value. **D**, **E** GO (**D**) and KEGG (**E**) enrichment analysis of 346 genes significantly correlated with SHCBP1 expression (*p* < 0.0001, *r* ≥ 0.5) in LUAD tissues (data downloaded from online database, http://www.cbioportal.org/). The enrichment analysis was performed by using the R package cluster Profiler. Only the top 10 scoring pathways are presented. **F**, **G** Kaplan–Meier overall survival curves of LUAD patients with high or low SHCBP1expression (stratified by median value) (**F**) and LUAD patients receiving radiotherapy with high or low SHCBP1expression (stratified by median value) (**G**). Data was downloaded from http://www.oncolnc.org/. *p* values were determined by log-rank test. HR, hazard ratio. **H** Western blot analysis of SHCBP1 expression in tumour tissues and paired normal tissues taken from 81 patients with surgically resected LUAD. T, tumour tissue; ANT, adjacent normal tissue. **I** SHCBP1 mRNA level in tumour tissues and paired normal tissues taken from 77 patients with surgically resected LUAD detected by Real-time quantitative PCR (qRT-PCR). Line graph analyzed by paired t-test, each data point represents the mean value of three technical replicates of one sample. T, tumour tissue; ANT, adjacent normal tissue. **J** Representative immunohistochemistry images of SHCBP1 and Ki67 staining in different clinical stages of patients with LUAD. Scale bars, 100um. The area surrounded by a box in stage III was magnified, which highlights the SHCBP1 staining in tumour cells. **K** Representative immunofluorescence images of SHCBP1 staining in two patients with LUAD. Scale bars, 50um. The area surrounded by a box was magnified. **L**, **M** Kaplan–Meier overall survival and progression-free survival curves of patients with surgically resected NSCLC (**L**) or LUAD (**M**) with high or low SHCBP1 expression. SHCBP1 expression was stratified by the immunohistochemistry staining score (high, score = 12; low, score < 12). **N**, **O** Immunohistochemical staining (**N**) and Kaplan–Meier survival curves (**O**) of SHCBP1 expression in tissue microarrays from 93 patients with LUAD from another hospital. #, refers to the last 6 patients without ANT. T, tumour tissue; ANT, adjacent normal tissue

To determine the clinical significance of SHCBP1 in NSCLC, we analyzed publicly available datasets (TCGA, Oncomine, and TIMER), clinical lung cancer specimens, and LUAD tissue microarray data to examine SHCBP1 expression in tumour and normal tissues, and the correlation of SHCBP1 with patient clinical characteristics. The clinical characteristics of 504 patients with LUAD from the TCGA database are shown in Table S1. Higher SHCBP1 expression were found in tumour tissues than in normal tissues of public database (Fig. 1A, B), and was significantly correlated with TNM clinical stage in patients with LUAD (all *p* < 0.05) (Table S1), positively correlated with MKI67 (proliferation marker), but negatively correlated with NKX2.1 (a differentiation marker) expression in tumour tissues (Fig. 1C). GO, BP and KEGG pathway enrichment analysis of 346 genes highly correlated with SHCBP1 (*r* ≥ 0.5, *p* < 0.0001) showed involvement of mainly chromosome separation, DNA replication, cell cycle and DNA damage repair pathways (Fig. 1D, E), indicating that high SHCBP1 expression in tumour tissues may play a role in tumour cell cycle progression and related DNA damage repair function. Survival analysis revealed that high SHCBP1 expression indicated poor patient survival (*p* = 0.0003, hazard ratio [HR] = 1.73 [95% CI, 1.29–2.23]) (Fig. 1F). Among the 59 LUAD patients who received radiotherapy, those with high SHCBP1 expression showed a worse survival prognosis (Fig. 1G), with a much higher HR than that in Fig. 1F (HR: 4.65 vs. 1.73), suggesting that LUAD patients with high SHCBP1 expression may be less sensitive to radiotherapy.

Moreover, 213 lung cancer tissues from Wuhan Union Hospital and LUAD tissue microarrays from another hospital were analyzed (Table S2, S3). SHCBP1 expression, quantified by the immunohistochemical (IHC) scoring, was also significantly correlated with patient tumour stages and ki67 index (*p* < 0.05) (Table S2, S3). Western blotting and qRT-qPCR analyses showed higher SHCBP1 expression in tumour tissues than in adjacent normal tissues (ANTs) (Fig. 1H–I), with most ANTs hardly expressing SHCBP1 (Fig. 1H). SHCBP1 and ki67 expression were synchronously increased with the increase of pathological stage in LAUD patients (Fig. 1J). In addition, SHCBP1 was found to be mainly expressed in the nucleus of cancer cells in NSCLC tissues, regardless of IHC or immunofluorescence staining (Fig. 1J, K), which may be related to its functions. Overall survival and progression-free survival (PFS) were consistent with previous reports [15, 16], in which high SHCBP1 level predicted poor patient survival (Fig. 1L-M). The IHC staining of SHCBP1 on LUAD tissue microarrays further validated that conclusion (Fig. 1N-O). Furthermore, most primary LUAD cells isolated from patients with malignant pleural effusion also showed relatively high and heterogeneous SHCBP1 expression (Supplementary Fig. 1A-B), as normal lung tissues hardly express SHCBP1 (Fig. 1H), and SHCBP1 mRNA levels were significantly correlated with EPCAM, MKI67, and PCNA mRNA levels (*r *> 0.8, *p* < 0.001) (Supplementary Fig. 1C-D). In general, the online public databases and LAUD specimens from two hospitals showed high SHCBP1 expression in cancer tissues, and the expression was closely correlated with patient survival and tumour proliferation. Additionally, patients with higher SHCBP1 expression tended to be less sensitive to radiotherapy.

### Dynamic expression and subcellular localization of SHCBP1 throughout the tumour cell cycle

#### SHCBP1 is mainly located in the nucleus and relatively highly expressed in the G2 and M phases of the cell cycle

**Fig. 2:**
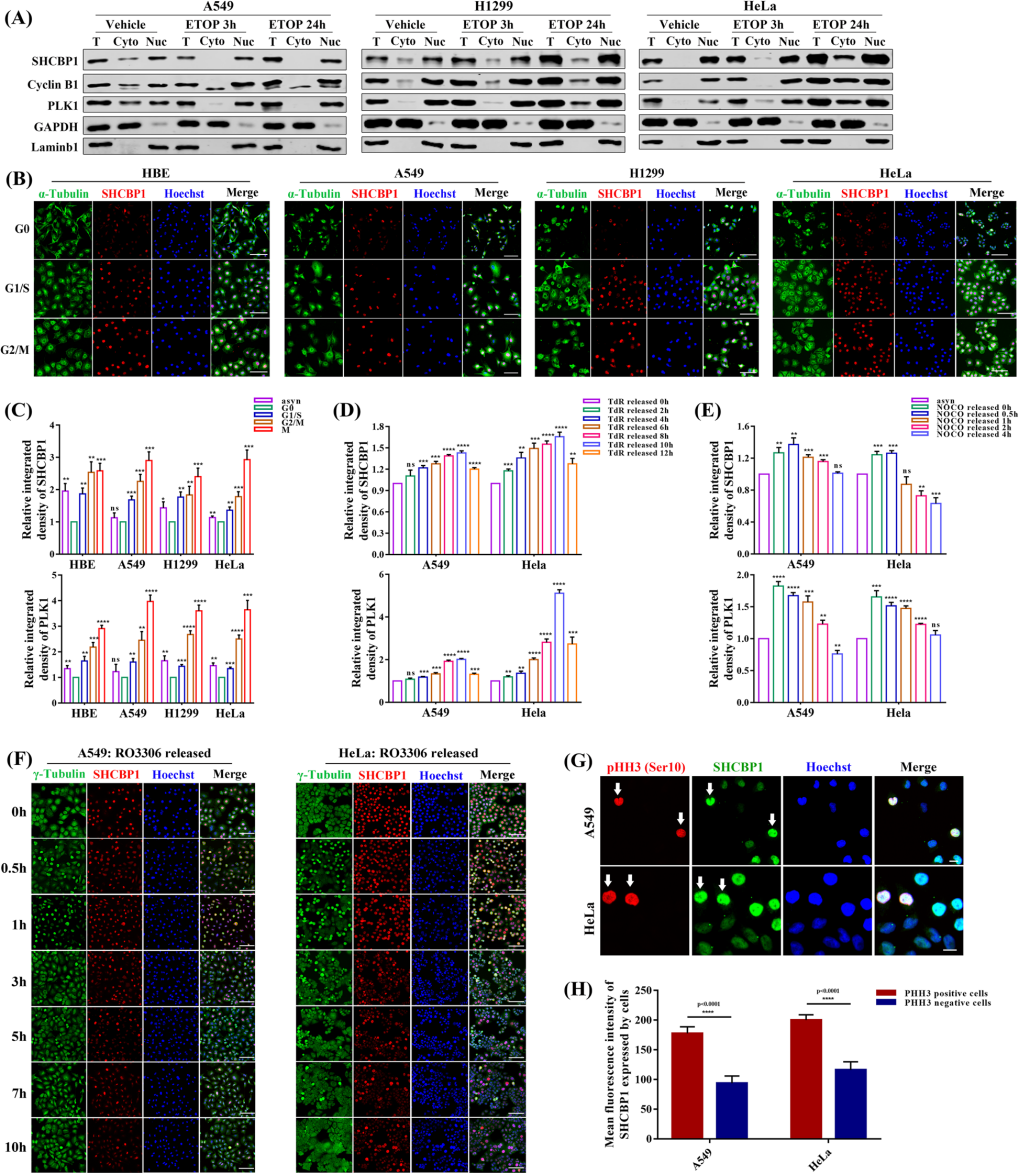
SHCBP1 is mainly located in the nucleus, with increased expression during the G2 and M phases of the cell cycle. **A** Representative western blot images of SHCBP1, Cyclin B1 and PLK1 protein in total (T), cytoplasmic (Cyto), and nuclear (Nuc) extracts of A549, H1299 and Hela cells after treating with low-dose ETOP (A549, 1μΜ; H1299, 5 μM; Hela, 3 μM) or vehicle for 3 h and 24 h. GAPDH and Lamin B1 were used as internal controls for cytoplasmic and nuclear extracts, respectively. **B**,** C** Immunofluorescence (IF) staining (**B**) and western blot densitometric analysis (**C**) of SHCBP1 and PLK1 throughout the cell cycle of HBE, A549, H1299 and HeLa cells (cells were synchronized to G0, G1/S, and G2/M phase by serum starvation, double thymidine and RO3306 block, respectively). In the IF staining (**B**), cells were co-stained with anti-SHCBP1 antibody (red), anti-α-tubulin antibody (green), and Hoechst (blue); Scale bars, 100 μm. Representative Western blot images of panel (**C**) are shown in Supplementary Fig. 4, data are expressed as mean ± SD (*n* = 3 independent experiments) and analyzed by the unpaired Student’s t test (ns, not significant; *, *P* < 0.05; **, *P* < 0.01; ***, *P* < 0.001; ****, *P* < 0.0001). Note: asyn, asynchronized. PLK1: well-known G2–M phase-associated proteins. **D**,** E** Western blot densitometric analysis of SHCBP1 and PLK1 in A549 and HeLa cells analyzed at indicated time points after nocodazole (NOCO) (**D**) and double thymidine (TdR) release (**E**), respectively. Data are expressed as mean ± SD (*n* = 3 independent experiments) and analyzed by the unpaired Student’s t test (ns, not significant; *, *P* < 0.05; **, *P* < 0.01; ***, *P* < 0.001; ****, *P* < 0.0001). **F** Immunofluorescence staining of SHCBP1 at indicated time points after RO3306 release in A549 and HeLa cells. Cells were co-stained with anti-SHCBP1 antibody (red), anti-γ-tubulin antibody (green), and Hoechst (blue). Scale bars, 100um. **G**, **H** Representative immunofluorescence images (**G**) showing co-localization of SHCBP1 (green) with PHH3 (Ser10) (red) and the corresponding statistical analysis (**H**) of SHCBP1 mean fluorescence intensity between PHH3 (Ser10) positive and negative cells. The PHH3 (Ser10) positive cells (mitotic cells) are highlighted by the white arrows; Scale bars, 20 μm. Data in panel (H) are expressed as mean ± SD (n ≥ 4, with at least 200 cells per analysis) and analyzed by the unpaired Student’s t test (****, *P* < 0.0001)

**Fig. 3:**
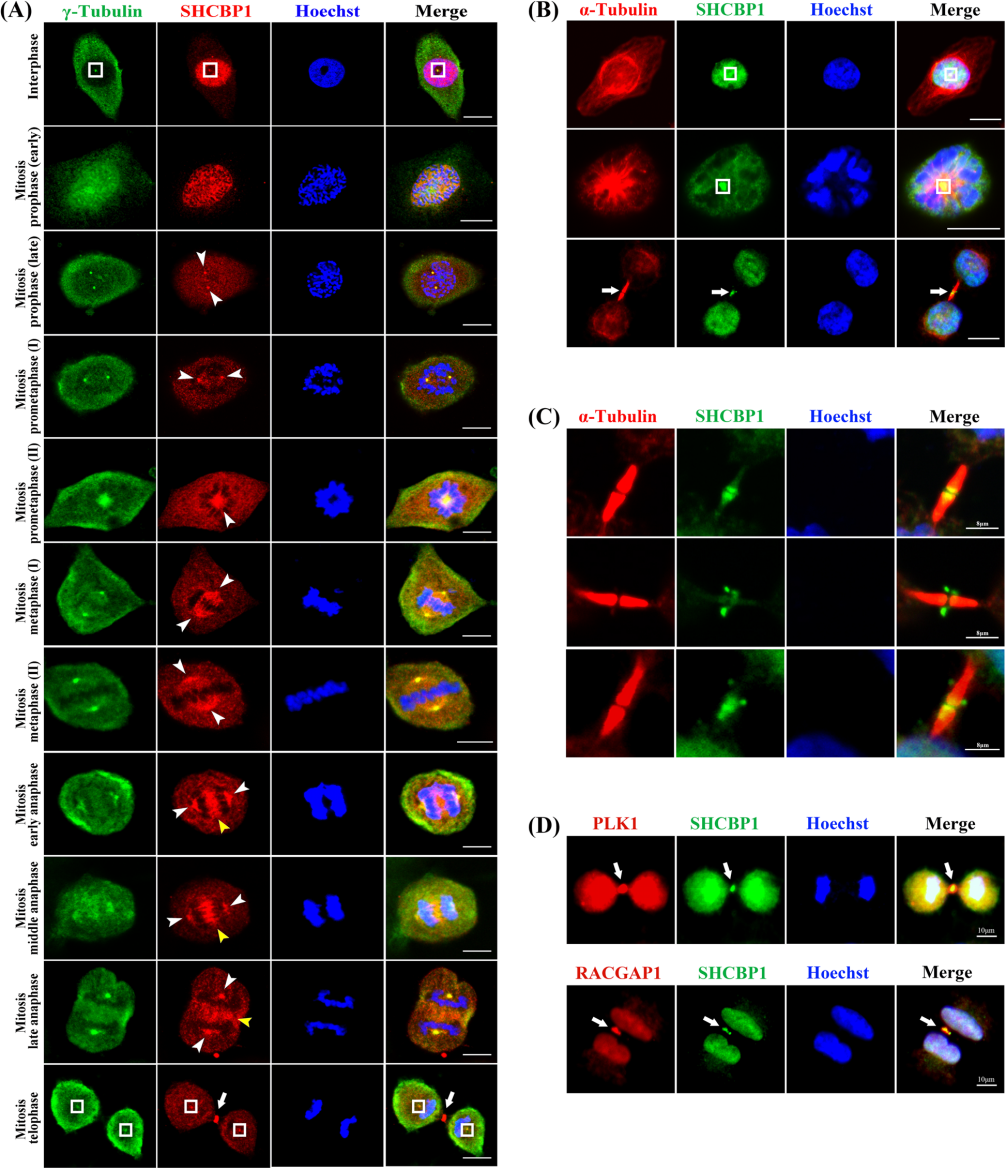
SHCBP1 dynamically localizes to centrosomes and different spindle sites during cell mitosis. **A** Immunofluorescence analysis of SHCBP1 localization (red) throughout the cell cycle (interphase; early and late prophase; prometaphase; metaphase; early, middle, and late anaphase; and telophase) as assessed by co-staining with γ-tubulin (green) and Hoechst (blue). White boxes mark the centrosome in the interphase or after cytokinesis; white-arrowheads indicate the spindle poles during the mitosis; yellow-arrowheads point to the central spindle; white arrows: midbody. Scale bars, 20 μm. **B** Immunofluorescence co-staining of SHCBP1 (green) and α-tubulin (red) in NSCLC cells. White boxes: centrosomes; white arrows: midbody. Scale bars, 20 μm. **C** Immunofluorescence co-staining of SHCBP1 (green) and α-tubulin (red) at the midbody. Scale bars, 8 μm. **D** Immunofluorescence co-staining of SHCBP1 (green) and PLK1 (red) or RACGAP1 (red) in NSCLC cells. White arrows indicate midbody. Scale bars, 10 μm

To understand why SHCBP1 was closely correlated with tumour proliferation, we first assessed its expression in human-derived cell lines, including immortalized bronchial epithelial (HBE), lung cancer (A549, NCI-H1299, NCI-H460, NCI-H292), HeLa, and 293wt cells. SHCBP1 expression was higher in all tumour cells than in immortalized HBE and 293wt cells (Supplementary Fig. 2A–C). Based on the SHCBP1 expression levels and the purpose of better studying the tumour cell cycle, we mainly used A549 (lowest expression, p53 wild type), NCI-H1299 (medium expression, p53 null), HeLa (highest expression, classic tumour tool cells that are widely used in cell cycle research), and HBE cells in the subsequent experiments. Previous correlation analysis of SHCBP1 showed that the cell cycle ranked first in the KEGG enrichment pathway, mainly involving chromosome separation and DNA replication (Fig. 1E). To determine the specific function of SHCBP1 in the cell cycle, we assessed its dynamic expression and subcellular localization throughout the cell cycle (Figs. 2 and 3). SHCBP1 protein was detected mainly in the nucleus of tumour cells, with a small amount present in the cytoplasm (Fig. 2A). Meanwhile, SHCBP1 co-localization and immunoprecipitation with the nucleolar protein NPM1 showed diffuse SHCBP1 expression in the nucleus but not the nucleoli (Supplementary Fig. 3A-B). When the cells entered mitosis, the nucleolus disappeared and SHCBP1 was concentrated at the spindle poles (Supplementary Fig. 3A). Moreover, the expression of SHCBP1 changed dynamically with the cell cycle (Fig. 2B–F), with the lowest expression in G0 phase, followed by G1 and S phases, and peaked in G2 and M phase (Fig. 2B, C). Natural release assays of nocodazole (synchronized in M phase), double thymidine (TdR) (synchronized in G1/S phases), and RO3306 (synchronized in late G2 phase) blocks further confirmed relatively higher SHCBP1 expression in G2 and M phase of the cell cycle (Fig. 2D–F). SHCBP1 expression gradually increased when entering G2–M phase (Fig. 2D), and decreased with M phase exit (Fig. 2E, F), comparable to the expression trends of PLK1 and Cyclin B1, both well-known G2–M phase-associated proteins (Supplementary Fig. 4). Moreover, SHCBP1, PLK1, and PHH3 (Ser10) expression in tumour cells gradually increased with increasing paclitaxel concentration, further demonstrating higher SHCBP1 expression in M phase (Supplementary Fig. 3C–E). Similarly, SHCBP1 expression in tumour cells increased after docetaxel (DTX) exposure (Supplementary Fig. 3F). The co-localization assay also showed higher SHCBP1 expression in PHH3 (Ser10)-positive cells than in PHH3 (Ser10)-negative cells (Fig. 2G, H).

#### SHCBP1 dynamically localizes to centrosomes and different spindle sites during cell mitosis

Next, we further explored its changes in localization during the cell cycle. Consistent with previous studies [20–22], SHCBP1 was observed in the centrosome, central spindle, and midbody of dividing cells (Fig. 3A–D). However, a panoramic view of the dynamic localization of SHCBP1 throughout the cell cycle has not yet been reported. Co-localization of SHCBP with γ- or α-tubulin in tumour cells continuously released from G2–M (RO3306) block at different time points showed that SHCBP1 changed from a diffuse and uniform nuclear distribution to aggregation in mitotic spindle-related sites when the cells entered the mitotic phase from interphase (Fig. 3A). In the prophase and metaphase of mitosis, SHCBP1 mainly dynamically accumulated in the spindle poles (centrosomes) and mitotic spindle sites; however, during anaphase to telophase, SHCBP1 gathered to the central spindle and then the midbody sites, finally participating in cytokinesis (Fig. 3A, B). SHCBP1 also accumulated at the centrosome during the interphase (Fig. 3A). Interestingly, SHCBP1 distribution varied in the midbody, from the end, on both sides, or in the middle of the microtubule bundles of the two daughter cells (Fig. 3C). SHCBP1 reportedly co-localized with the centralspindlin complex (RACGAP1 and MKLP1) at the midbody and was responsible for cytokinesis [20, 21]. A recent study further demonstrated the physical interaction of SHCBP1 with PLK1 in mitotic cells to promote tumour cell mitosis through an SHCBP1-PLK1-MISP axis. Our findings also showed the high coincidence of SHCBP1 with the centralsplindlin complex and PLK1 regardless of the expression and distribution (Fig. 3D, Supplementary Fig. 3G-J). However, we only observed the physical interaction of SHCBP1 with RACGAP1 and MKLP1, but not with PLK1 in asynchronous tumour cells (Supplementary Fig. 3K), suggesting that SHCBP1 may only interact with PLK1 in the mitotic phase.

### SHCBP1 knockdown promotes premature mitotic entry by compromising the WEE1-mediated G2–M checkpoint

**Fig. 4:**
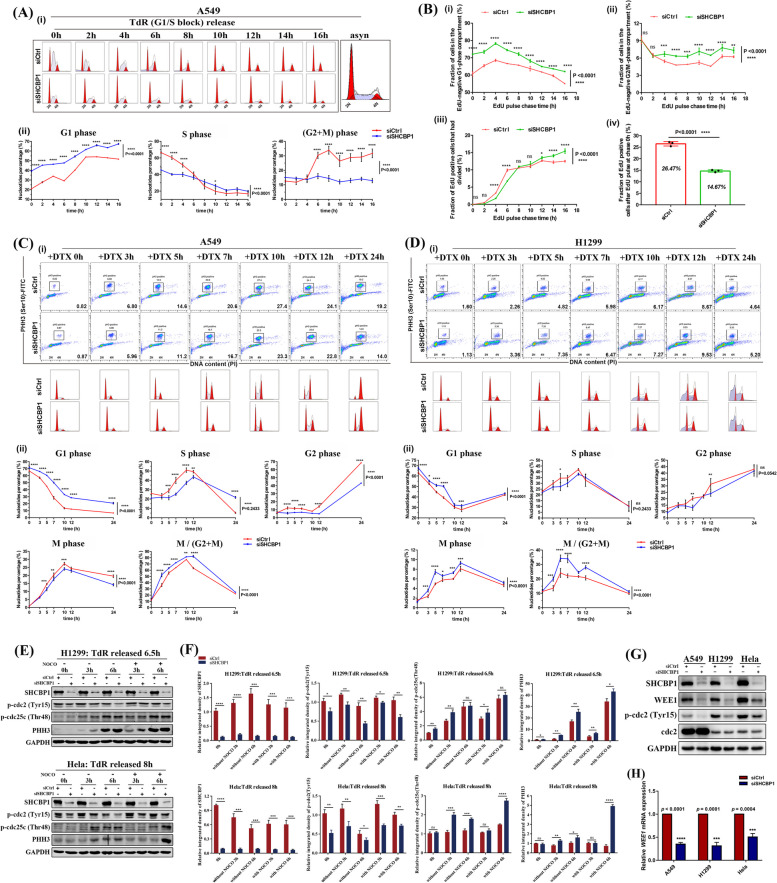
SHCBP1 knockdown slows tumour cell cycle but promotes premature mitotic entry by compromising the WEE1-mediated G2–M checkpoint.** A** Cell cycle analysis of A549 cells transfected with control or SHCBP1 siRNA at indicated time points after double thymidine (TdR) release. (**i**), flow cytometry plots of cells released at various time points and normally growing asynchronous (asyn) cells analyzed by using ModFit LT 5.0 and FlowJo software. Cells were stained with or without PHH3 (Ser10)-FITC antibody followed by propidium iodide (PI) staining. PHH3 (Ser10) positive cells indicating cells in M phase have been marked by small boxes on the dot plot. (**ii**) the line plot shows the change trend of cell proportion in each cell cycle phase (cell proportion of G1, S, G2 and M phase to the whole cell cycle, and the M phase to the G2 + M [4N] phase) over the released time. Data are expressed as mean ± SD (*n* = 3 independent experiments) and analyzed by the two-way ANOVA test (ns, not significant; *, *P* < 0.05; **, *P* < 0.01; ***, *P* < 0.001; ****, *P* < 0.0001). **B** Click-EdU pulse chase flow cytometry analysis of A549 cells. Cells transfected with siCtrl or siSHCBP1 were pulsed with 10 µM EdU for 45 min, and then changed to medium containing 20 µM thymidine to chase and analyze cells at 0, 2, 4, 6, 8, 10, 12, 14, 16, 18 h, cells were harvested at indicated time point for click-EdU reaction and PI staining and then subjected to flow cytometry analysis. Fractions of cells in the EdU-negative G1-phase compartment (i), EdU-negative G2/M phase compartment (ii), fraction of EdU positive cells that have divided (iii), and fraction of EdU positive cells at chase 0 h (iv) were analyzed. Data are expressed as mean ± SD (*n* = 3 independent experiments) and analyzed by the two-way ANOVA test (i-iii) and unpaired Student’s t test (iv), respectively (ns, not significant; *, *P* < 0.05; **, *P* < 0.01; ***, *P* < 0.001; ****, *P* < 0.0001). **C**, **D** A549 (**C**) and NCI-H1299 (**D**) cells transfected with control or SHCBP1 siRNA were subjected to 0.1 μM docetaxel (DTX) treatment for 0 h, 3 h, 5 h, 7 h, 10 h, 12 h and 24 h for cell cycle analysis, respectively. The legends of (i) and (ii) are similar to those in **A**. **E**, **F** NCI-H1299 and HeLa cells transfected with control or SHCBP1 siRNA were synchronized to late G2 phase after the indicated times of release from double thymine (TdR), followed by treatment with nocodazole (NOCO) or vehicle for 0 h, 3 h and 6 h. Representative western blot images of SHCBP1, phos-cdc2(Tyr15), phos-cdc25c(Thr48) and PHH3 (Ser10) with GAPDH as internal reference at each time point (**E**) and the corresponding density analysis (**F**) are shown. Data are expressed as mean ± SD, *P* values were determined by unpaired Student’s t test (ns, not significant; *, *P* < 0.05; **, *P* < 0.01; ***, *P* < 0.001; ****, *P* < 0.0001). **G** Representative western blot images of WEE1, p-cdc2(Tyr15) and total cdc2 in A549, NCI-H1299 and HeLa cells analyzed after SHCBP1 knockdown. **H** WEE1 mRNA expression of A549, NCI-H1299 and HeLa cells relative to GAPDH mRNA expression in cells transfected with control or SHCBP1 siRNA are shown. Relative WEE1 mRNA expression of siSHCBP1 group was calculated as a fold-change versus the siCtrl group. Data are expressed as mean ± SD (*n* = 3 independent experiments) and analyzed by the unpaired Student’s t test (***, *P* < 0.001; ****, *P* < 0.0001)

To test whether the high expression and dynamic localization of SHCBP1 in M phase was closely associated with its functions in tumour cells, we performed SHCBP1 knockdown in NSCLC (A549 and NCI-H1299) and HeLa cell lines to observe their effects on cell cycle progression (Fig. 4A–E). The knockdown efficiencies of them in these cells are shown in Supplementary Fig. 5. Cells transfected with control (siCtrl) or SHCBP1 siRNA (siSHCBP1) were synchronized to the G1–S phase by double TdR block and then released into regular media, with samples harvested at different time points (Fig. 4A, B). DNA histograms obtained by flow cytometry of all cells at different time points after release from the TdR block are shown in Supplementary Fig. 6. The cell cycle progression of A549 cells lagged in siSHCBP1 group, with flat cell proportion curves during each phase (G1, S, G2–M), while the curve slopes of each phase in the siCtrl group were significantly higher than those of the siSHCBP1 group (Fig. 4A). Consistently, click-EdU pulse-chase FCM assay that ensures uninterrupted cell cycle also showed inactive cell cycle progression in A549 cells, such as delayed G1 phase (Fig. 4B i) and arrested G2/M phase (Fig. 4B ii), as well as lagging cell division (Fig. 4B iii). Similarly, when EdU was washed off after incubation for 45 min, there were significantly fewer EdU positive cells in the siSHCBP1 group than in the siCtrl group, which further indicated that the cell proliferation was inhibited after SHCBP1 knockdown (Fig. 4B iv). However, in HeLa cells, the curve lag of the siSHCBP1 group was not as obvious as that in A549 cells (Supplementary Fig. 7A), indicating that A549 cells may be more sensitive to SHCBP1 inhibition. However, the change curve of cell proportions in the G2 phase of HeLa cells peak at 8 h after release from TdR block but showed lower proportions in the siSHCBP1 group than in the siCtrl group (G2 phase curve in Supplementary Fig. 7A ii). Correspondingly, the M phase (PHH3 positive) curve showed higher proportions of cells in the siSHCBP1 group compared to the control group from 4 to 8 h after release (M phase curve in Supplementary Fig. 7A ii), indicating premature M phase entry after SHCBP1 inhibition. But the proportion of G1 phase cells in the siSHCBP1 group was lower than that in the siCtrl group at 12–16 h after release (G1 phase curve in Supplementary Fig. 7A ii), suggesting that the prematurely entered M-phase cells did not progress into the G1 phase normally (i.e., failed mitotic exit).

To further verify our hypothesis, we transfected A549, NCI-H1299, and HeLa cells with siCtrl or siSHCBP1 siRNA and then treated them with 0.1 μM DTX, which arrest cells at M-phase, with a peak effect at 12–16 h, at different time points to observe the differences in cell proportion from the G1 to M phases (Fig. 4C-D and Supplementary Fig. 7B). The changes of A549 cells in the siSHCBP1 group during each cell cycle phase still lagged behind those in the siCtrl group, but with higher proportions of M-phase cells in the 4N (G2 + M) phase curve in the siSHCBP1 group during continuous exposure (Fig. 4C ii). Compared to the siCtrl group, NCI-H1299 and HeLa cells in the siSHCBP1 group consistently showed higher proportions of M-phase cells throughout the cell cycle or in the 4N (G2 + M) phase curve (Fig. 4D and Supplementary Fig. 7B). These findings demonstrated that SHCBP1 knockdown in tumour cells forced cells to prematurely enter the M phase.

Therefore, we inferred that the activity of CDK1 (also cdc2), a key kinase that regulates the transition of cells from G2 to M phase, might be dysregulated. CDK1 activation requires dephosphorylation of Tyr15 residues [28, 29]. To test this, we first synchronized NCI-H1299 and HeLa cells to the turning point of the G2–M phase by releasing cells from TdR double-block for indicated time, and then released them again into fresh media for 0, 3, and 6 h (with or without nocodazole) to observe the M-phase entry of cells from the G2 phase. Detection of M-phase entry (determined by PHH3 and phos-cdc25c [Thr48]) and phos-cdc2 [Tyr15]) after SHCBP1 knockdown by western blotting showed that SHCBP1 inhibition increased the mitotic index and significantly reduced phos-cdc2 (Tyr15) level regardless of nocodazole exposure (Fig. 4E-F, Supplementary Fig. 8A–C). Our findings suggested that the early M phase entry after SHCBP1 inhibition occurred due to decreased CDK1-Tyr15 phosphorylation, which removed the inhibition of CDK1 activity and increased the cell transition from G2 to M phase. WEE1 kinase is a key gatekeeper of the G2–M transition through inhibitory phosphorylation of CDK1 at the conserved Tyr15 residues and is an effective anti-cancer target [6, 30–32], we subsequently examined the expression of WEE1 kinase protein and mRNA levels after SHCBP1 knockdown in three tumour cell lines (Fig. 4G, H). Consistent with our supposition, WEE1 mRNA and protein and p-cdc2 (Tyr15) protein levels were both significantly down-regulated after SHCBP1 knockdown, while total cdc2 protein was not. In general, our experimental results in the three cell lines showed that SHCBP1 knockdown induced premature M-phase entry during cell cycle by damping the WEE1-pcdc2 (Tyr15) axis, the gatekeeper of the G2–M phase transition.

### SHCBP1 knockdown causes multipolar spindle formation and delays mitotic exit in tumour cells

**Fig. 5:**
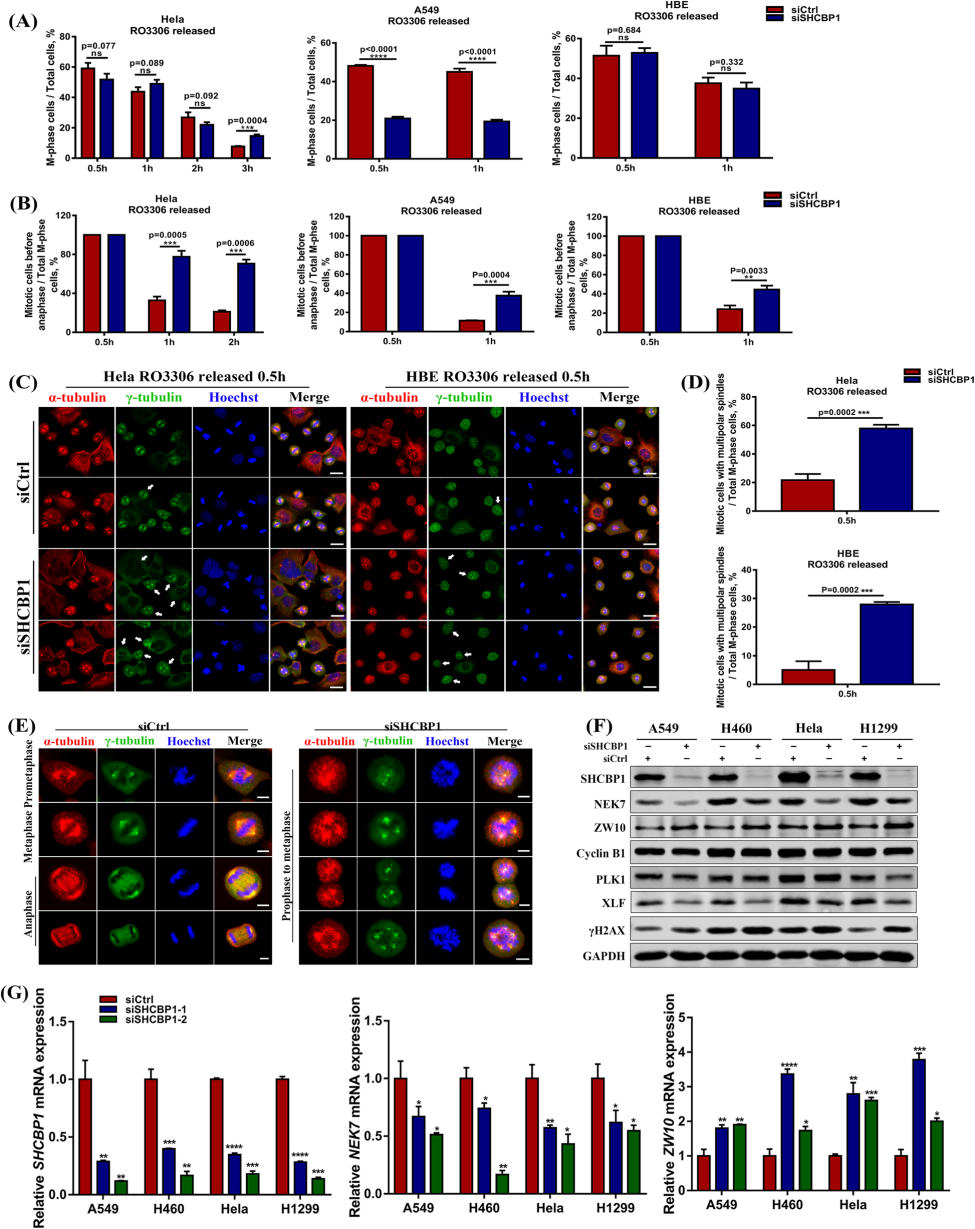
SHCBP1 knockdown causes multipolar spindle formation and delays mitotic exit in tumour cells. **A** The percentage of mitotic cells counted in HeLa, A549 and HBE cells transfected with control or SHCBP1 siRNA after release from RO3306 block (9μΜ) for indicated time. Immunofluorescence staining for α-tubulin (red) and γ-tubulin (green) followed by confocal fluorescence imaging and cell counting were performed at each time point. Three different fields at each time point were randomly selected for counting at least 500 cells within each field. Data are shown as mean ± SD (*n* = 3) and analyzed by the unpaired Student’s t test. ***, *P* < 0.001; ****, *P* < 0.0001; ns = not significant. **B** The percentage of mitotic cells prior to anaphase in total M-phase cells was analyzed from the same experiment in (**A**). Three different fields at each time point were randomly selected for counting at least 200 mitotic cells within each field. Data are shown as mean ± SD (*n* = 3) and analyzed by the unpaired Student’s t test. **, *P* < 0.01; ***, *P* < 0.001. **C** Representative immunofluorescence images of α-tubulin (red) and γ-tubulin (green) staining after 0.5 h release from RO3306 arrest (9 μM) in HeLa and HBE cells transfected with siCtrl or siSHCBP1. White arrows indicate the cells with multipolar spindle in M phase. Scale bars, 20 μm. **D** The percentage of cells with multipolar spindle in M phase over total mitotic cells in the same experiment from (**C**) were analyzed. Three different fields were randomly selected for counting at least 200 mitotic cells within each field. Data are shown as mean ± SD (*n* = 3) and analyzed by the unpaired Student’s t test. ***, *P* < 0.001. **E** Representative immunofluorescence images of multipolar spindle formation throughout M phase in HeLa cells transfected with SHCBP1 siRNA compared to siCtrl cells with normal spindle pole formation throughout M phase. α-tubulin (red), γ-tubulin (green), and Hoechst (blue) were co-stained in cells. Scale bars, 5 μm. **F**, **G** Western blot images (**F**) and mRNA expression (**G**) analysis of changes in NEK7, ZW10, cyclin B1, PLK1, XLF and γH2AX in A549, NCI-H460, HeLa and NCI-H1299 cells transfected with SHCBP1 siRNA compared to controls. GAPDH was used as internal reference. Data are shown as mean ± SD (*n* = 3) and analyzed by the unpaired Student’s t test. *, *P* < 0.05; **, *P* < 0.01; ***, *P* < 0.001; ****, *P* < 0.0001

As previously described, HeLa cells showed not only premature mitotic entry but also delayed mitotic exit (Fig. 4B iii). To unravel the mechanism behind this observation, HeLa, A549, and HBE cells transfected with siCtrl or siSHCBP1 were synchronized to the late G2 phase by RO3306 block and released into fresh media for 0.5, 1, 2, and 3 h, respectively. Cells were collected for immunofluorescence staining for α- and γ-tubulin, as well as Hoechst, to observe mitosis entry and exit (Supplementary Fig. 8D–F, Fig. 5A, B). These cells reached the M phase peak at 0.5 and 1 h after RO3306 release. Most had exited the M phase at 2 h and 3 h, during which time almost no round mitotic cells were observed except for in HeLa cells (Supplementary Fig. 8D–F). HeLa cells had more round mitotic cells in the siSHCBP1 group than in the siCtrl group at 3 h after RO3306 release (Fig. 5A), indicating that SHCBP1 inhibition affected cell exit from mitosis, while the proportion of M-phase cells in the A549 siSHCBP1 group at 0.5 and 1 h was lower (Fig. 5A), consistent with previous finding that A549 was more sensitive to siSHCBP1 treatment and presented significantly slowed cell cycle progression after SHCBP1 knockdown. To explain why mitotic exit was disrupted, we analyzed the proportions of mitotic-cells-before-anaphase to all M-phase cells at 0.5 and 1 h after RO3306 release (Fig. 5B). Almost all M-phase cells were in prophase to metaphase at 0.5 h after release and most began to enter the anaphase and subsequently exit the M phase at 1 h or 2 h after release (Supplementary Fig. 8D-F, Fig. 5B). However, at 1 or 2 h after RO3306 release, the proportion of mitotic-cells-before-anaphase to the total M-phase cells was significantly higher in the siSHCBP1 group than in the control group (Fig. 5B), suggesting that SHCBP1 inhibition prevented mitotic cells from entering anaphase, precluding successful M phase exit. We further found that HeLa and HBE cells released from the RO3306 block at 0.5 h in the siSHCBP1 group presented many mitotic cells containing multipolar (mostly tripolar) spindles (Fig. 5C), about 3–fourfold more than in the siCtrl cells (Fig. 5D). Therefore, mitotic cells could not move smoothly from metaphase to anaphase, resulting in delayed M-phase exit (Fig. 5E).

To determine the reasons for the multipolar spindle formation and subsequent suppression of the metaphase-anaphase transition in tumour mitotic cells after SHCBP1 inhibition, we screened a series of centrosome and spindle assembly checkpoint (SAC) related proteins (data not shown). NEK7 expression in four tumour cell lines was significantly down-regulated while ZW10 were upregulated, regardless of protein and mRNA levels (Fig. 5F, G). NEK7 is the smallest NIMA-related kinase in mammals; its role in mitosis includes centrosome enrichment and microtubule nucleation, which are important for interphase centrosome replication, prophase centrosome separation, and metaphase spindle assembly [33–36]. NKE7 inhibition leads to defective mitotic spindles and induces multipolar spindle formation [36–38]. Therefore, reduced NEK7 expression indicates centrosome and mitotic spindle disorder. We have previously demonstrated that SHCBP1 localized on centrosomes throughout the cell cycle (Fig. 3). Therefore, SHCBP1 may maintain the functions of tumour cell centrosomes and spindles by regulating NEK7 expression. Zw10 protein is a kinetochore component necessary for proper SAC activity in both *Drosophila* and mammals [39] to ensure correct chromosome separation at the beginning of the anaphase of mitosis [40–45]. These findings indicate the importance of ZW10 as a SAC component to maintain the high fidelity of mitosis. Thus, the up-regulation of ZW10 expression in the siSHCBP1 cells indicated SAC activation to delay the metaphase-anaphase transition. Also, we found that after WEE1 knockdown, the NEK7 expression at mRNA and protein levels were down-regulated while the ZW10 expression was up-regulated, which was consistent with SHCBP1 knockdown.

### SHCBP1 knockdown inhibits tumour growth and increases cell apoptosis and senescence

**Fig. 6:**
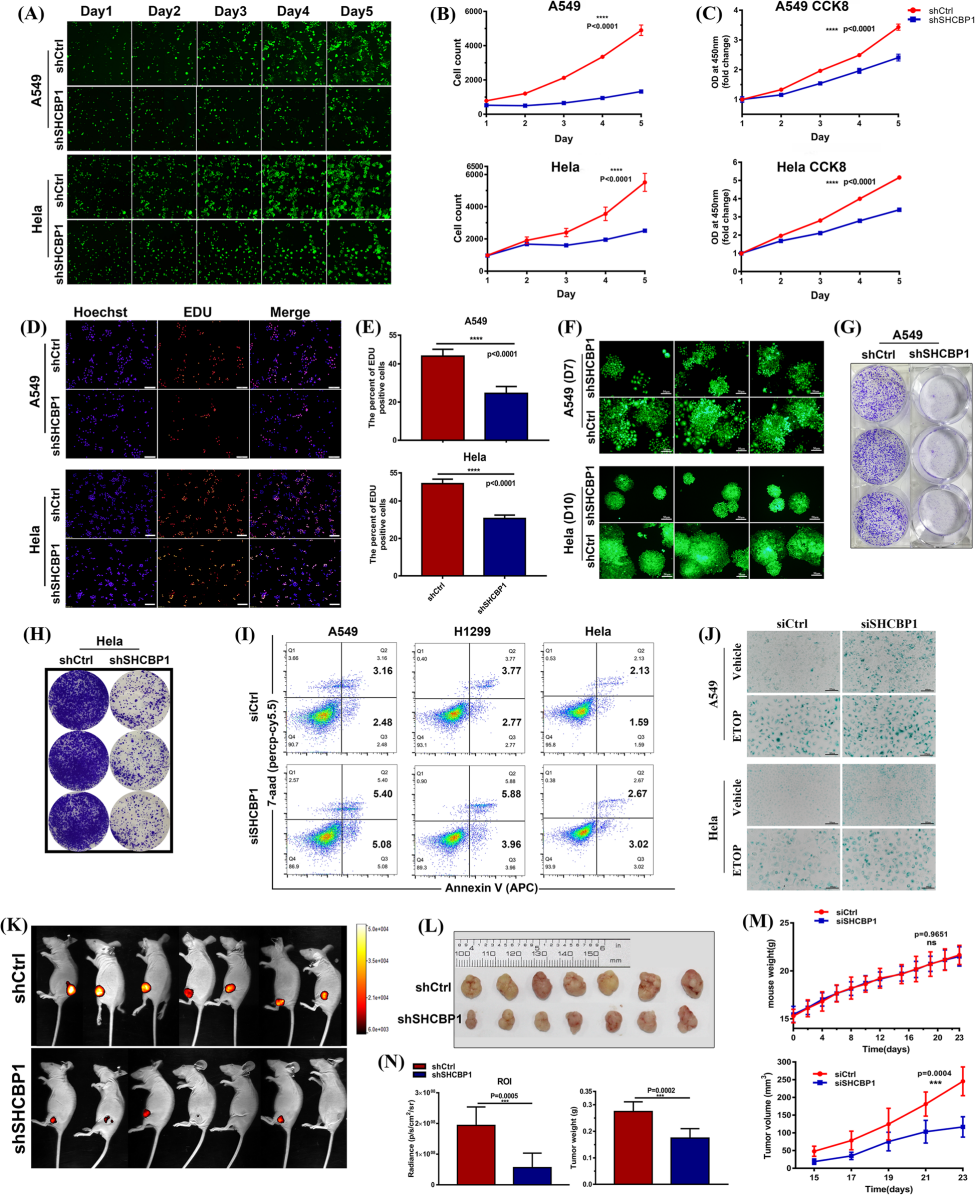
SHCBP1 knockdown inhibits tumour growth and increases cell apoptosis and senescence. **A**-**B** Representative images (**A**) and cell counts (**B**) of live-cell imaging of A549 and HeLa cell growth for 5 consecutive days following infection with green fluorescent protein (GFP)-tagged shCtrl or shSHCBP1 lentiviruses for 72 h. Only cells with green fluorescence were counted. Three wells were repeated in each group, and four visual fields were randomly selected from each well for cell counting; Data are presented as mean ± SD and analyzed by two-way analysis of variance [ANOVA]; ****, *P* < 0.0001. **C** CCK8 assay in A549 and HeLa cells after 72 h infection with GFP-tagged shCtrl or shCBP1 lentivirus. Data are expressed as mean ± SD (*n* = 5 per group). *P* value was determined by two-way ANOVA test. **D**, **E** Representative images (**D**) and quantification (**E**) of EdU incorporation assays in A549 and HeLa cells after 96 h infection with shCtrl or shSHCBP1 lentivirus. At least five visual fields with a minimum of 200 cells per field were randomly selected for cell counting. Data are expressed as mean ± SD, and the *p* value was determined by the unpaired Student’s t test. **F–H** The proliferative ability of A549 and HeLa cells infected with GFP-tagged shCtrl or shCBP1 lentivirus was determined through a plate colony formation assay. Representative fluorescence images (**F**) at the indicated time and colonies stained with 0.5% crystal violet (**G**, **H**) at the 14th day are shown. Scale bars, 50 μm. **I** Representative flow cytometry plots of apoptosis in A549, NCI-H1299 and HeLa cells 48 h after transfection with control or SHCBP1 siRNA. The cells were stained with 7-AAD and APC-labeled Annexin V prior to recognition of early (Annexin V + /7-AAD -) and late (Annexin V + /7-AAD +) apoptosis. **J** Microscopy of senescence associated β-galactosidase staining cells (blue) in A549 and HeLa cells 72 h after transfection with control or SHCBP1 siRNA or transfected with siRNA for 48 h followed by 24 h incubation with low-dose etoposide (A549 cells, 1 μm; HeLa cells, 3 μM); Scale bars, 100 μm. **K-N** BALB/c nude mice (7 mice per group) were injected subcutaneously with 5 × 10^6^ A549-LUC cells (stably expressing luciferase) that had been infected with shCtrl or shSHCBP1 lentivirus for 4–5 days, respectively. In-vivo bioluminescence imaging (**K**) and isolated subcutaneous tumours (**L**) of two groups of tumour-bearing mice at the end point (23 days after tumour inoculation) are shown. **M** Mouse weight and tumour growth curve util the end point; **N** the average tumour radiance (bioluminescence intensity, p/sec/cm.^3^/sr) and tumour weight at the end point were analyzed. *P* values were determined by the two-way ANOVA test and unpaired Student’s t test, respectively. ns = not significant; ***, *P* < 0.001; ****, *P* < 0.0001

As SHCBP1 played a critical role in cell cycle progression, we then sought to verify whether targeting SHCBP1 could inhibit tumour growth in vitro and vivo. After infection with shSHCBP1 or shCtrl lentivirus, the growth of A549 and HeLa cells were visualized and quantified using the Celigo imaging cytometer for 5 consecutive days. Consistent with previous findings [46, 47], SHCBP1 knockdown significantly inhibited A549 and HeLa cell proliferation (Fig. 6A-B). The results of longitudinal CCK8, EdU incorporation, and clonogenic assays also confirmed this finding (Fig. 6C-H). Moreover, cell apoptosis and senescence were increased in SHCBP1 siRNA treated A549, NCI-H1299, and HeLa cells (Fig. 6I, J). Out of these, A549 showed the most significant apoptosis (Fig. 6I) and a more obvious phenomenon in senescence (Fig. 6J), indicating that tumour cells with low SHCBP1 expression were more sensitive to SHCBP1 intervention, but the different residual SHCBP1 levels after knockdown may also account for this phenomenon.

In addition, SHCBP1 inhibition in the in vivo A549 subcutaneous xenograft (Fig. 6K, L) and the tail-vein injected LLC metastasis mouse models (Supplementary Fig. 9) also remarkably attenuated tumour growth. During this period, the body weight growth curves of mice in the shCtrl and shSHCBP1 groups did not differ significantly; however, tumour growth was significantly slower in the shSHCBP1 group than that in the shCtrl group (Fig. 6M, Supplementary Fig. 9E-G). Meanwhile, the fluorescence intensity and tumour weight of mouse subcutaneous tumours differed significantly between the two groups (Fig. 6N).

### Targeting SHCBP1 in combination with low-dose DNA-damaging drugs triggers mitotic catastrophe in tumour cells due to G2–M checkpoint abrogation

**Fig. 7:**
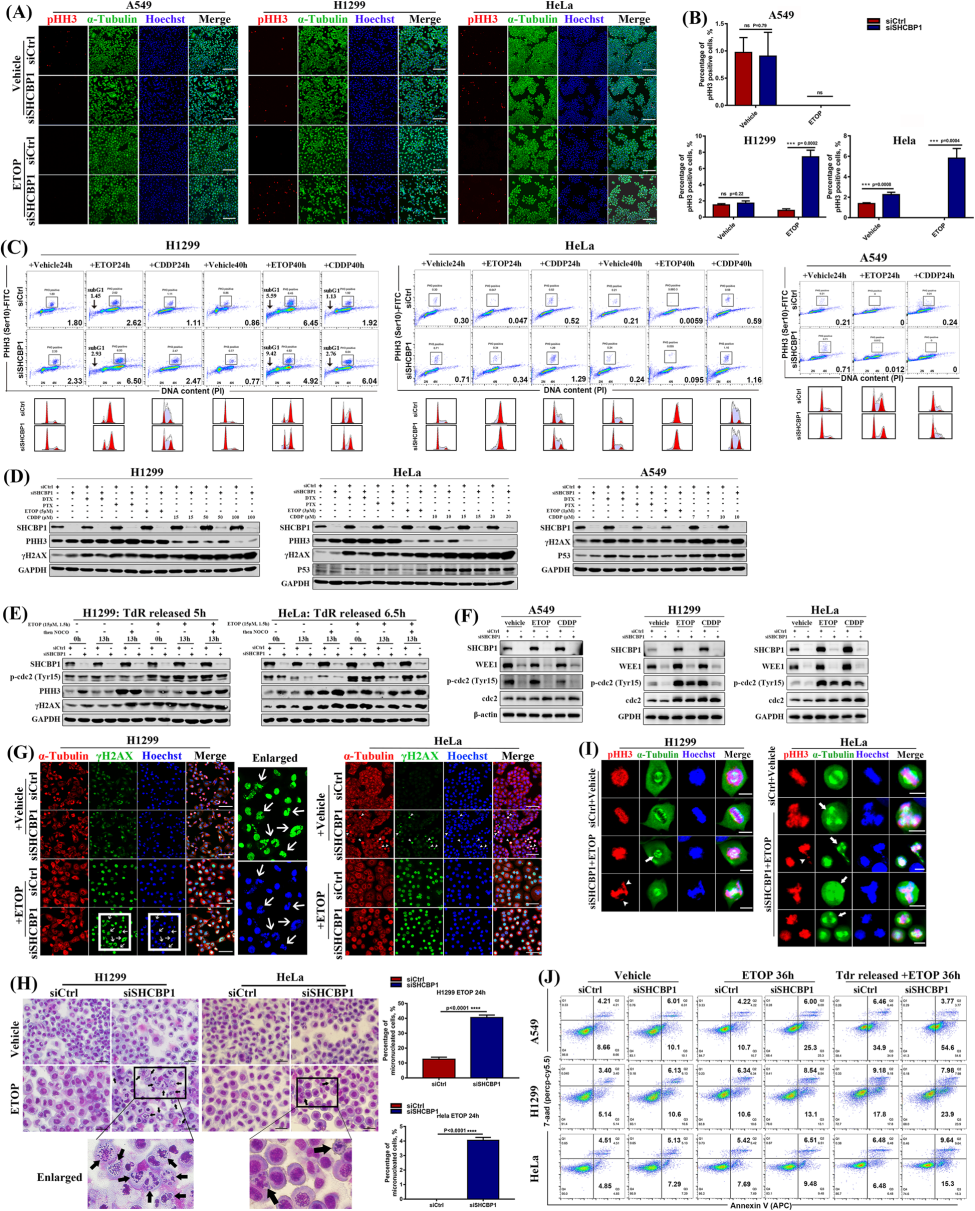
Targeting SHCBP1 in combination with low-dose DNA-damaging drugs triggers mitotic catastrophe in tumour cells due to G2–M checkpoint abrogation.** A**, **B** A549, NCI-H1299 and HeLa cells were transfected with control or SHCBP1 siRNA for 24 h, and then treated with low-dose etoposide (A549 cells, 1 μM; NCI-H1299 cells, 5 μM; HeLa cells, 3 μM) or vehicle for 24 h, respectively. Representative immunofluorescence images of PHH3 (red) and α-tubulin (green) co-staining (**A**) and statistics of PHH3-positive cell counts (**B**) are shown. Scale bars, 200 μm. At least three fields of view were randomly selected with at least 300 cells per field for cell counting. Data are expressed as mean ± SD and analyzed by unpaired Student’s t test; ns = not significant; ***, *P* < 0.001. **C** NCI-H1299, HeLa and A549 cells were transfected with control or SHCBP1 siRNA for 24 h, followed by treatment with either low-dose etoposide (ETOP, same as in **A** and **B**) or cisplatin (CDDP) or corresponding vehicle (DMSO) for 24 h or 40 h; CDDP: NCI-H1299 (10 μM), HeLa (5 μM), A549 (5 μM). Flow cytometry plots and histograms of cells stained with PHH3 (Ser10)-FITC antibody and propidium iodide (PI) are shown. Mitotic cells that are positive for PHH3(Ser10) were circled out by small boxes. Black arrows indicate the sub G1-phase cells. **D** Western blot analysis of SHCBP1, PHH3(Ser10), γH2AX and P53 in NCI-H1299, HeLa and A549 cells, cells were transfected with control or SHCBP1 siRNA for 24 h, followed by 24 h treatment with various chemotherapeutic drugs, respectively; docetaxel (DTX), paclitaxel (PTX), etoposide (ETOP) and cisplatin (CDDP). GAPDH was used as internal reference. **E** NCI-H1299 and HeLa cells transfected with control or SHCBP1 siRNA were synchronized in G2 phase by allowing progression for indicated time after double thymidine release and then pulsed with 15 μM etoposide for 1.5 h, then washed out into fresh media with or without nocodazole (NOCO) for 13 h. Western blot analysis of the SHCBP1, p-cdc2(Tyr15), PHH3, and γH2AX was performed in these cells with GAPDH as internal reference. **F** Western blotting was performed for SHCBP1, WEE1, p-cdc2 (Tyr15) and total cdc2 in A549, NCI-H1299 and HeLa cells which were transfected with control or SHCBP1 siRNA for 24 h, followed by incubation with vehicle solution, low-dose ETOP or CDDP (concentrations are the same as previously described) for 24 h, respectively. **G** HeLa and NCI-H1299 cells transfected with control or SHCBP1 siRNA for 24 h were subjected to low-dose ETOP (3 μM for HeLa cells and 5 μM for H1299 cells) or corresponding vehicle for another 24 h. Representative immunofluorescence images of α-tubulin (red) and γH2AX (green) co-staining in these cells are shown (scale bars, 100 μm). The right panel of NCI-H1299 cells displays the magnified views highlighted in the left panel by the white boxes, and the white arrows indicate the cells undergoing mitotic catastrophe with micronuclei formation. The white-arrowheads in the HeLa cells highlight cells with higher γH2AX level after SHCBP1 knockdown. **H** NCI-H1299 and HeLa cells received the same treatment as in (**G**), and stained with Liu stain for quick checking (left panel). The area marked by the box is magnified, and the black arrows indicate cells undergoing mitotic catastrophe with micronuclei formation; Scale bars, 50 μm. Histogram (right panel) shows the percentage of micronucleated cells over total cells. Data are expressed as mean ± SD and analyzed by unpaired Student’s t test; ****, *P* < 0.0001. **I** NCI-H1299 and HeLa cells received the same treatment as in (**G**). Representative immunofluorescence images of PHH3 (red) and α-tubulin (green) co-staining in cells using single-photon confocal microscope. White arrows indicate cells with multipolar spindles. White-arrowheads indicate chromosomal fragmentation. Scale bars, 20 μm. **J** A549, HeLa, and NCI-H1299 cells transfected with control or SHCBP1 siRNA for 24 h were subjected to low-dose ETOP for 36 h or synchronized through TdR release, followed by exposure to low-dose ETOP for 36 h. Representative flow cytometry plots show co-staining with 7-AAD and APC-labeled Annexin V for identification of early (Annexin V + /7-AAD-) and late (Annexin V + /7-AAD +) apoptosis

Our previous data showed that SHCBP1 knockdown led to early M-phase entry by downregulating WEE1 expression and subsequently reducing CDK1 phosphorylation at Tyr15, resulting in the abrogation of the G2–M checkpoint in tumour cells (Fig. 4). As cell survival is highly dependent on cell cycle arrest after DNA damage, and targeting the G2–M checkpoint in tumour cells combined with DNA-damaging treatment are getting more and more attention [48–50], we then examined the anti-tumour effects of the combination of siSHCBP1 with DNA-damaging agents. We observed increased SHCBP1 protein levels in tumour cells with escalating doses of DNA-damaging agents (within a cell-lethal dose) exposure, including etoposide (ETOP), cisplatin (CDDP), and radiation (Supplementary Fig. 10A-C). The same phenomena were observed in SHCBP1 immunostaining after low-dose ETOP treatment (Supplementary Fig. 10D), with the increased SHCBP1 expression mainly existed in the nucleus (Fig. 2A). These results indicated that SHCBP1 may participate in the DNA damage response. Moreover, as has been reported, we found that tumour cells were arrested at the G2 phase to varying degrees after 20 h of low-dose ETOP administration, while they were arrested in the G1/S phase at 24 h after low-dose CDDP administration, with gradual S or G2 phase arrest after 48 h of CDDP treatment (Supplementary Fig. 11). Then, we detected the percentage of PHH3-positive M-phase tumour cells after SHCBP1 knockdown combined with low-dose ETOP or CDDP treatment by immunostaining assay, flow cytometry, and western blotting analysis (Fig. 7A–D). After ETOP or CDDP administration, almost no PHH3-positive cells were observed in the siCtrl group of A549 and HeLa cells, indicating they were arrested in the G2 or G1/S phase. But the siSHCBP1 group showed PHH3 positivity in HeLa but not A549 cells (Fig. 7A), the latter may be due to the severely inhibited cell cycle progression. Several PHH3-positive cells were observed in the siCtrl group of p53-mutant NCI-H1299 cells after ETOP or CDDP administration for 24 or 40 h; however, the percentage of PHH3-positive cells was significantly higher in the siSHCBP1 group than that in the siCtrl group (Fig. 7A–C). The real-time imaging video of NCI-H1299 and HeLa cells treated with SHCBP1 siRNA combined with low-dose ETOP showed similar effects, as reflected by the earlier appearance of rounded mitotic cells (Supplementary video 1 and 2). In addition, the proportion of sub-G1 cells in NCI-H1299 and HeLa cells in the siSHCBP1 group also increased significantly after ETOP or CDDP administration (Fig. 7C), consistent with increased γH2AX levels in these tumour cells (Fig. 7D). In contrast, γH2AX and p53 expression decreased in A549 and HeLa cells treated with SHCBP1 siRNA combined with docetaxel (DTX) or paclitaxel (PTX) (Fig. 7D), indicating that targeting SHCBP1 combined with DNA-damaging agents enhanced the anti-tumour effect, which might be attenuated when combined with microtubule-toxic drugs. This was also verified by CCK8 assay (Supplementary Fig. 12).

To confirm that the abnormal M phase entry in cells treated with SHCBP1 siRNA combined with DNA-damaging agents was due to G2–M checkpoint abrogation, we synchronized cells to G2 phase by allowing cell progression for 5–7 h after double thymidine release and then exposed them to 15 μM etoposide for 1.5 h, followed by release into fresh media with or without nocodazole to analyze the G2-phase cells entering the M phase after DNA damage. NCI-H1299 and HeLa cells stopped at the G2 phase and did not enter the M phase until 13 h after ETOP removal (Fig. 7E and Supplementary Fig. 13), whereas these cells started entering the M phase 3–6 h after vehicle removal (Fig. 4F). As expected, 13 h after ETOP or vehicle removal, higher PHH3 expression was detected in the siSHCBP1 group compared to the siCtrl group; Meanwhile, lower levels of p-cdc2 (Tyr15) and higher levels of γH2AX were observed in SHCBP1 siRNA-treated cells (Fig. 7E). Similarly, asynchronous A549, NCI-H1299 and HeLa cells treated with low-dose ETOP or CDDP for 24 h showed decreased WEE1 and p-cdc2 (Tyr15) levels after SHCBP1 knockdown (Fig. 7F). These data demonstrated that targeting SHCBP1 in tumour cells abrogated WEE1-phosphorylated cdc2 (Tyr15) axis-mediated maintenance of the G2–M checkpoint, which is responsible for the premature mitotic entry with unrepaired DNA damage.

The DNA-damaging agent ETOP induced G2 arrest in tumour cells, while SHCBP1 inhibition prompted cells with unrepaired DNA damage to override this arrest and enter the M phase (Fig. 7A–D), which may trigger the onset of mitotic catastrophe and lead to cell death [7, 51–53]. Consistent with our hypothesis, nearly 40% of NCI-H1299 cells treated with SHCBP1 siRNA combined with low-dose ETOP showed micronuclei cells, accompanied by higher γH2AX expression (Fig. 7G, H). Likewise, HeLa cells showed a consistent phenomenon, but was less pronounced than that in NCI-H1299 cells (Fig. 7G, H). In addition, cells in the siSHCBP1 group with unscheduled mitotic entry after ETOP administration also showed aberrant mitosis, mainly characterized by lagging or broken chromosomes and multipolar spindle formation during cell division (Fig. 7I). Consistently, we observed a higher frequency of cell apoptosis in the siSHCBP1 group after ETOP administration compared to the siCtrl group combined with ETOP (Fig. 7J). This difference was more pronounced when ETOP was administered after TdR synchronization (Fig. 7J), which may be related to the increased chance of cells simultaneously entering the M phase after synchronization.

### Targeting SHCBP1 in combination with low-dose DNA-damaging drugs compromises DNA repair

**Fig. 8:**
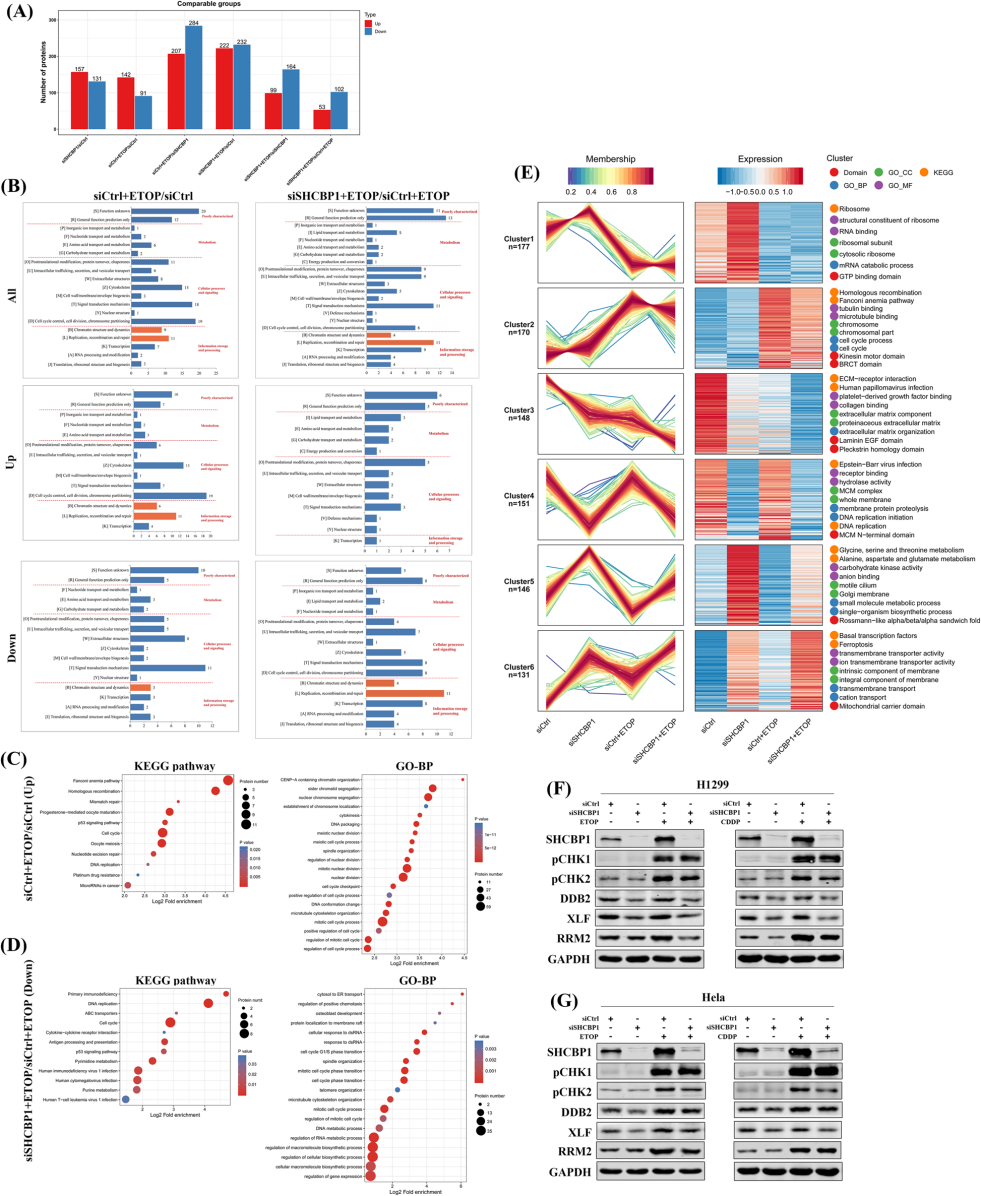
Targeting SHCBP1 in combination with low-dose DNA-damaging drugs compromises DNA repair function. NCI-H1299 cells were transfected with control or SHCBP1 siRNA for 24 h and then treated with 5 μM etoposide or corresponding vehicle for 24 h. The cells were collected for proteomic analysis to identify differentially expressed proteins (DEPs) between siCtrl, siSHCBP1, siCtrl + ETOP, siSHCBP1 + ETOP. **A** Number of DEPs in siSHCBP1 vs. siCtrl, siCtrl + ETOP vs. siCtrl, siCtrl + ETOP vs. siSHCBP1, siSHCBP1 + ETOP vs. siCtrl, siSHCBP1 + ETOP vs. siSHCBP1 and siSHCBP1 + ETOP vs. siCtrl + ETOP groups, respectively. **B** Histograms representing clusters of orthologous groups (COG) classification in siCtrl + ETOP vs. siCtrl and siSHCBP1 + ETOP vs. siCtrl + ETOP. The identified differential proteins were classified into four categories (“poorly characterized”, “metabolism”, “cellular processes and signaling” and “information storage and processing”) according to the COG analysis. The horizontal axis represents the number of corresponding differential proteins. The clusters of “Chromatin structure and dynamics” and “replication, recombination and repair” in the category of information storage and processing are highlighted in orange. **C**, **D** KEGG pathway and GO (Gene Ontology) analysis of biological process (GO-BP) for the up-regulated DEPs between siCtrl + ETOP vs. siCtrl (**C**) and the down-regulated DEPs between siSHCBP1 + ETOP vs. siCtrl + ETOP (**D**). **E** DEPs clustered by their expression pattern across the different treatment group were achieved by applying fuzzy c-means clustering analysis using the R package Mfuzz. Left panel: Six clusters of DEPs with different expression patterns (n = number of DEPs). Right panel: Heatmaps of each cluster with the top two KEGG pathways and GO analyses of molecular function (GO_MF), cell component (GO_CC), biological process (GO_BP) and protein domain are displayed. **F**, **G** NCI-H1299 (**F**) and HeLa cells (**G**) were subjected to the same treatment as in A-E (24 h siRNA transfection followed by 24 h low-dose ETOP or CDDP exposure), cells were collected for western blot analysis of SHCBP1, phosphorylated-CHK1, phosphorylated-CHK2, DDB2, XLF and RRM2 with GAPDH as internal reference. NCI-H1299 (5 μM ETOP, 10 μM CDDP); HeLa (3 μM ETOP, 5 μM CDDP)

To further investigate the molecular mechanisms underlying the beneficial effects of the combination strategy, NCI-H1299 cells were transfected with control or SHCBP1 siRNA and then incubated with low-dose ETOP for 24 h for unbiased proteomic analysis. Together, 288, 233, 491, 454, 263, and 155 differentially expressed proteins (DEPs) were detected in siSHCBP1 versus siCtrl, siCtrl + ETOP versus siCtrl, siCtrl + ETOP versus siSHCBP1, siSHCBP1 + ETOP versus siCtrl, siSHCBP1 + ETOP versus siSHCBP1 and siSHCBP1 + ETOP versus siCtrl + ETOP groups, respectively (Fig. 8A). Clusters of Orthologous Groups of proteins (COG/KOG) analysis classified the DEPs into four functional clusters (Fig. 8B). DEPs related to “chromatin structure and dynamics” and “replication, recombination, and repair” function, which were classified into the information storage and processing functions, were enriched in comparable numbers in the siCtrl + ETOP versus siCtrl and siSHCBP1 + ETOP versus siCtrl + ETOP groups. However, these DEPs were increased and decreased in the siCtrl + ETOP versus siCtrl and siSHCBP1 + ETOP versus siCtrl + ETOP groups, respectively (Fig. 8B). KEGG and GO analyses were further conducted. The top enriched functions revealed that DNA damage repair pathways, such as the Fanconi anemia, homologous recombination (HR), and mismatch repair pathways; the cell cycle process, including sister chromatid segregation, spindle organization; and cell cycle checkpoint process, were significantly up-regulated in the siCtrl + ETOP versus siCtrl group, while the DNA replication and the cell cycle pathways were down-regulated in the siSHCBP1 + ETOP versus siCtrl + ETOP group (Fig. 8C, D). These data indicated activation of the DNA repair system and cell cycle checkpoint in NCI-H1299 cells treated with DNA-damaging agent, but some of these precise controls were lost after siSHCBP1 combined treatment.

Given the above observations, we then performed a soft clustering analysis using Mfuzz package [54] to detail the change patterns of DEPs among these four groups. Proteins in the same clusters showed similar expression trends [54]. Six cluster types were finally enriched according to the DEPs (Fig. 8E). The top two KEGG pathways and GO results are displayed. Consistently, the DEPs of cluster 2, with HR and cell cycle process enrichment, showed slight increases in these DEPs in the siSHCBP1 group compared to the siCtrl, with dramatic increases after ETOP treatment; however, DEPs in the siSHCBP1 + ETOP group did not increase to the same level as in the siCtrl + ETOP group, suggesting insufficient DNA damage repair and cell cycle checkpoint after the combination strategy. The same expression change pattern could be found in P53 wild-type A549 cells, but the magnitude of change was not as obvious as that of H1299 cells. However, it can be seen from the figure that cell cycle inhibition occurs in almost all clusters, which further indicates that the proliferation of A549 cells is significantly inhibited after SHCBP1 knockdown (Supplementary Fig. 14). Moreover, the DEPs in cluster 6 showed an increased trend in ferroptosis after SHCBP1 knockdown, a trend that was more obvious after the combination of SHCBP1 knockdown and ETOP administration (Fig. 8E), suggesting another effective factor contributing to tumour lethality.

Based on the proteomic findings, we then examined the expression of DNA-damage associated proteins, including phospho-CHK1, phospho-CHK2, DDB2, XLF, and RRM2 in the NCI-H1299 and HeLa cell lines after the same treatment (Fig. 8F, G). Western blotting analysis showed decreased pCHK2 but not pCHK1 levels in the siSHCBP1 + ETOP group compared to the siCtrl + ETOP group; DDB2 and XLF showed a downward trend after SHCBP1 knockdown with or without ETOP, but RRM2 only decreased in the NCI-H1299 cells (Fig. 8F, G). Altogether, targeting SHCBP1 in combination with low-dose DNA-damaging drugs not only attenuated tumour cell cycle checkpoints but also compromised DNA repair, leading to the synergistic effects of the combination strategy.

### SHCBP1 knockdown synergistically enhances the tumour-killing effects of DNA-damaging drugs in vivo

**Fig. 9:**
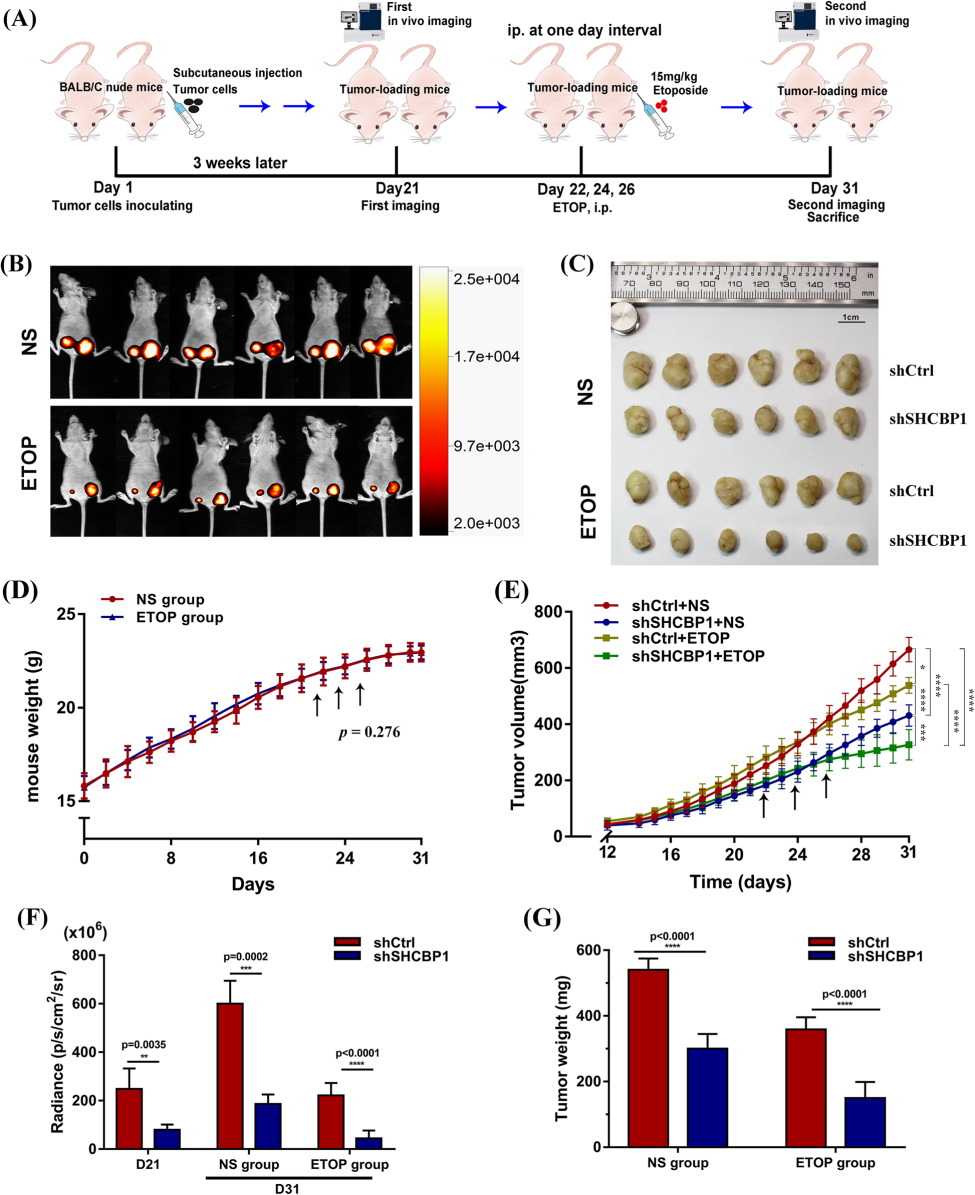
SHCBP1 knockdown synergistically enhances the tumour-killing effects of DNA-damaging drugs in vivo. BALB/c nude mice (6 mice per group) were injected subcutaneously in both flanks with 5 × 10^5^ HeLa-LUC cells (stably expressing luciferase) that had been infected with shCtrl or shSHCBP1 lentivirus for 4–5 days, respectively. The tumour-bearing mice were given etoposide (15 mg/kg) or the normal saline (NS) through intraperitoneal injection every other day when the subcutaneous tumour reaching around 200 cm.^3^, and the mice were finally sacrificed 31 days after tumour inoculation (end point). **A** Schematic diagram of the whole process of the tumour-bearing mouse experiment. **B**, **C** In-vivo bioluminescence imaging (left: shSHCBP1, right: shCtrl) (**B**) and isolated subcutaneous tumours (**C**) of two groups of tumour-bearing mice at the end point (31 days after tumour inoculation) are shown. **D**, **E** mouse weight (**D**) and tumour growth curve (**E**) util the end point among the groups; Black arrows represent the time points of etoposide or NS intervention. Data are expressed as mean ± SD and analyzed by the two-way ANOVA test; *, *P* < 0.05; ***, *P* < 0.001; ****, *P* < 0.0001. **F**, **G** Average tumour radiance (p/sec/cm3/sr) (**F**) of shCtrl and shSHCBP1 groups were analyzed in NS and ETOP groups on days 21 and 31 after tumour inoculation. Tumour weight (**G**) of shCtrl and shSHCBP1 groups were analyzed in NS and ETOP groups at the end point. Data are expressed as mean ± SD, *p* values were determined by the paired t-test. **, *P* < 0.01; ***, *P* < 0.001; ****, *P* < 0.0001

To test the anti-tumour effects of the combination strategy in vivo, we established xenografts of HeLa-LUC cells pre-infected with shCtrl or shSHCBP1 lentivirus in nude mice and began treatment with relatively low-dose ETOP (intraperitoneal, 15 mg/kg, every other day, 3 times) once the tumours reached ~ 200 mm^3^ in size (Fig. 9A). The expression of SHCBP1 in mouse subcutaneous tumour tissues was also detected by western blot analysis throughout the modeling period (Supplementary Fig. 15). While both shSHCBP1 and ETOP monotherapies moderately slowed tumour growth, combination therapy significantly repressed tumour growth more than either treatment alone (Fig. 9B, C). The mouse weight-gain curves did not differ among the four groups, demonstrating that the low-dose ETOP did not cause severe adverse effects (Fig. 9D). However, the subcutaneous tumour growth curve differed significantly among the four groups, indicating that the combination strategy with shSHCBP1 and ETOP significantly delayed the tumour growth compared to any monotherapy (Fig. 9E). Moreover, the tumour radiance intensity and weight on the 31st day after tumour inoculation were higher in the shCtrl/shSHCBP1 + normal saline (NS) group than in the shSHCBP1 + ETOP group (Fig. 9F, G), indicating that the combination strategy induced a synergistic tumour-killing effect.

## Discussion

This study revealed the spatiotemporal distribution of SHCBP1 during the cell cycle process and demonstrated that G2–M checkpoint abrogation (downregulated WEE1 expression) and mitotic multipolar spindle formation (dysregulated NE7 and ZW10 expression) in tumour cells were caused by SHCBP1 inhibition. These changes forced tumour cells to prematurely enter the M phase and subsequently experienced spindle dysfunction in the M phase, leading to tumour cell cycle disruption and tumour suppression in vitro and vivo. Moreover, we found that low-dose DNA-damaging agents can upregulate the expression of SHCBP1. Based on the theory that tumour cell survival during DNA damage largely depends on efficient cell cycle checkpoints, we further demonstrated that the combination of SHCBP1 inhibition and low-dose DNA-damaging drugs had synergistic effects on tumouor therapy. Mechanistically, SHCBP1 inhibition attenuated DNA-damaging drug-induced G2 phase arrest by compromising the WEE1-mediated G2–M checkpoint, subsequently inducing mitotic catastrophe and enhancing DNA damage. However, SHCBP1 knockdown combined with tubulin-toxic drugs weakened the killing effect of the drugs on tumour cells, which is an interesting phenomenon that deserves further study. Moreover, we suggest that tumours with lower SHCBP1 expression are more sensitive to DNA-damaging agents, which may aid in the selection of chemotherapeutic agents in clinical practice.

Our findings demonstrated the important clinical role of SHCBP1 in tumour progression. SHCBP1 is not only an effective prognostic indicator but also a potential biological treatment target, which was confirmed in this study of tumour data collected from three different clinical sources (TCGA database, Union Hospital clinical sample, and out-of-hospital tissue array). These data revealed upregulated SHCBP1 expression in the tumour tissues of patients and verified the association of SHCBP1 with clinical stage and survival prognosis in patients with NSCLC, consistent with previous findings on SHCBP1 [15, 16, 46]. In addition, compared with that of patients in the SHCBP1 low-expression group, the survival prognosis of LUAD patients in the SHCBP1 high-expression group who received radiotherapy was worse, indicating that lung cancer patients with low SHCBP1 expression are more sensitive to radiotherapy.

Mechanistically, We revealed the spatiotemporal expression of SHCBP1 during the cell cycle process, as indicated by the relatively upregulated expression in the G2 and M phases and its dynamic change of distribution in the cell cycle, with localization to the centrosome, co-localization with the centrasplindlin complex (RACGAP1, MKLP1) at different spindle sites during the mitosis, and gathering to the midbody during the cytokinesis, consistent with previous studies reporting that SHCBP1 binds to the central spindle, recruits cytokinesis-related proteins, and promotes cell division [20–22]. Therefore, SHCBP1 knockdown increased the tendency for aberrant mitosis which may be responsible for tumour growth inhibition. Consistently, we found that SHCBP1 knockdown induced multipolar spindle formation and delayed mitotic exit in tumour cells, which also caused moderate DNA damage in the treated tumour cells, and the downregulated NEK7 expression and upregulated ZW10 expression caused by the SHCBP1 inhibition may account for the disrupted mitosis. Previous studies demonstrated that NEK7 is a centrosomal kinase required for proper spindle formation during mitosis [33–35]. And NEK7 knockdown results in various mitotic defects, including multipolar spindle phenotypes [33], mitotic delay at metaphase with fragile mitotic spindles [35], and apoptosis following mitotic arrest [35]. Incorrect chromosome-spindle attachments during mitosis can activate the mitotic checkpoints, which in turn arrest cells in the M ​​phase [55]. In this study, we found that the protein and mRNA levels of Zw10 were up-regulated. Researchers have reported that Zw10 and rough deal (Rod) are new components of the SAC [41, 42]. In the case of kinetochore misattachment to the mitotic spindle, the RZZ (Rod, ZW10 and Zwilch) complex and Mad1 protein bind to the kinetochore to induce a spindle checkpoint signal to block cells from staying in the metaphase until the kinetochore and the mitotic spindle are properly connected [44, 45]. So we consistently observed a delayed transition of cells from metaphase to anaphase during mitosis (Fig. 5A, B), leading to the delayed mitotic exit.

In addition, we found that the premature M-phase entry in tumour cells after SHCBP1 knockdown was due to WEE1 kinase downregulation, which resulted in decreased CDK1 phosphorylation at the Tyr15 residue. CDK1 activation requires the dephosphorylation at residues Thr14 and Tyr15 and plays a key role in G2–M phase transition [28, 29]. WEE1 is a nuclear serine/threonine protein kinase that directly phosphorylates the Tyr15 residue of CDK1 to inactivate and induce cell cycle arrest at the G2–M transition [56, 57]. Therefore, targeting SHCBP1 in tumour cells could abrogate the G2–M checkpoint by inhibiting the WEE1-pCDK1 (Tyr15) axis. However, despite premature mitotic entry, tumour cell cycle progression was slowed, and this change was accompanied by a slight increase in γH2AX levels (Fig. 5F). These effects occurred due to subsequent mitotic disturbances (increased multipolar spindle formation and delayed metaphase-to-anaphase transition), resulting in delayed mitotic exit and DNA damage. In other words, cells that prematurely enter the M-phase prematurely experience spindle disorders and DNA-damaging lesions. Researchers have shown that targeting WEE1 kinase results in extensive replication stress, such as aberrant replication firing, increased fork degradation, and nucleotide deprivation [58–63], as well as an override of DNA damage checkpoints, leading to premature mitotic entry [7, 32], making it an effective target for inducing cell death. In our study, targeting SHCBP1 showed a consistent strong inhibitory effect on tumour progression both in vitro and in vivo, likely mainly through WEE1 kinase downregulation. Besides, three different siRNAs targeting WEE1 kinase also showed downregulated NEK7 expression and upregulated ZW10 expression, which was consistent with the findings of SHCBP1 knockdown, suggesting that targeting SHCBP1 may exert antitumour effects partly by dysregulating WEE1-NEK7-ZW10 axis.

An increasing number of studies suggest that compromising the G2–M checkpoint may allow enhanced genotoxic drug therapy [48–50, 64], prompting us to explore the synergistic tumour-killing effects of targeting SHCBP1 combined with low-dose DNA-damaging agents in vitro and in vivo. We found that low-dose DNA-damaging agents can induce increased expression of SHCBP1 (Supplementary Fig. 10). Moreover, clinical TCGA data suggested increased sensitivity to radiotherapy and longer survival in patients with low SHCBP1 expression (Fig. 1G). Most chemotherapy and radiation therapies directly damage DNA or target the basic cellular and metabolic processes that indirectly lead to DNA damage. DNA damage response (DDR) triggers cell cycle checkpoint activation, promoting repair by pausing the cell cycle or, in the case of irreparable DNA damage, triggering programmed cell death [65, 66]. Therefore, abrogating the DNA damage-induced G2–M checkpoint by SHCBP1 inhibition poses additional cytotoxicity, as cells subjected to siSHCBP1 enter mitosis and attempt chromosome segregation with extensive DNA damage, ultimately leading to mitotic catastrophe and cell death. Consistent with these findings, after ETOP or CDDP administration, we observed an increased mitotic index in siSHCBP1-treated NCI-H1299 and HeLa cells compared to that in control cells, followed by an increased number of cells with micronucleation phenotypes and elevated γH2AX levels. This phenomenon was more prominent in NCI-H1299 cells, suggesting that cells with TP53 mutations, which are deficient in the G1–S checkpoint and fully dependent on the G2–M checkpoint, were more sensitive to this combination. Similarly, in preclinical models of cancer, G2 checkpoint inhibition has been shown to enhance the efficacy of genotoxic drugs [9], preferentially sensitized TP53-deficient tumour cells to DNA damage [67–69].

Additionally, mass spectrometry analysis of NCI-H1299 and A549 cells treated with SHCBP1 siRNA and low-dose ETOP further indicated that the combination treatment not only compromised the cell cycle checkpoint but also attenuated DNA damage repair, as revealed by decreased levels of DNA damage repair proteins, including pCHK2, DDB2, XLF, RRM2 and etc. Therefore, the homologous recombination (HR) function and Fanconi anemia pathway were relatively dampened (Fig. 8E). Similarly, WEE1 kinase inhibitors could compromise HR through CDK1-mediated phosphorylation of BRCA1 and BRCA2 [70–72]. Therefore, siSHCBP1-mediated WEE1 kinase downregulation may account for these two effects (compromised cell cycle checkpoint and DNA damage repair function) in tumour therapy. These data suggest that targeting SHCBP1 could disrupt cell cycle homeostasis and attenuate the DNA repair system, thereby sensitizing tumour cells to DNA-damaging agents.

Collectively, our findings demonstrated SHCBP1 involvement in cell cycle regulation, and verified that SHCBP1 knockdown delayed cell cycle progression, promoted premature mitotic entry and multipolar spindle formation, inhibited M phase exit, and promoted cell apoptosis and senescence to inhibit tumour progression. Moreover, SHCBP1 inhibition in tumour cells abrogated the G2–M checkpoint by downregulating the WEE1 kinase to break the balance between genotoxic stress and the cell cycle arrest and repair system, resulting in further tumour growth inhibition. This effect was largely augmented when combined with low-dose DNA-damaging agents, leading to mitotic catastrophe and tumour cell death. Our results provide a preclinical rationale for the role of SHCBP1 in predicting tumour prognosis and as a therapeutic target. In recent years, targeting WEE1 kinase has attracted great interest in cancer therapy, and clinical trials of WEE1 inhibitors as monotherapies or combination chemotherapies are ongoing in patients with refractory solid tumours [73–76]. Obviously, SHCBP1 may also be a promising target for the treatment of lung cancer patients in the near future, especially those with TP53 mutations, either alone or in combination with DNA-damaging agents.

### Supplementary Information

**Additional file 1: Table S1.** Clinical characteristics of LUAD patients with different SHCBP1 expression from the TCGA dataset. **Table S2.** Clinical characteristics of NSCLC patients with different SHCBP1 expression from the Wuhan Union Hospital. **Table S3.** Clinical characteristics of LUAD patients with different SHCBP1 expression from the tissue microarray. **Table S4.** The primer sequences of the target genes for real‐time PCR.

**Additional file 2: Supplementary Figure 1.** Expression of SHCBP1 in isolated lung adenocarcinoma cells of patients with malignant pleural effusion. **Supplementary Figure 2.** Expression of SHCBP1 in different LUAD and other cell lines. **Supplementary Figure 3.** SHCBP1 is highly consistent with the expression of Mitotic phase-associated proteins. **Supplementary Figure 4.** SHCBP1 expression changed with the cell cycle progression. **Supplementary Figure 5.** Knockdown efficiency of different small interfering RNAs of SHCBP1. **Supplementary Figure 6.** Representative DNA histograms (A) and statistical bar chart (B) obtained by flow cytometry of all cells at different time points after release from the TdR block. **Supplementary Figure 7.** SHCBP1 knockdown slows tumor cell cycle but promotes premature mitotic entry in Hela cells. **Supplementary Figure 8.** Effect of SHCBP1 knockdown on tumor cells entering and exiting M phase. **Supplementary Figure 9.** SHCBP1 knockdown inhibits tumor proliferation and metastasis in C57BL/6 mice. **Supplementary Figure 10.** Expression of SHCBP1 is elevated after treatment with DNA-damaging agents. **Supplementary Figure 11.** Cell cycle arrest after low-dose etoposide and cisplatin treatment. **Supplementary Figure 12.** CCK 8 assay in tumor cells after SHCBP1 knockdown combined with docetaxel (A) or etoposide exposure (B). **Supplementary Figure 13.** Tumor cells transfected with siCtrl or siSHCBP1 siRNA did not enter M phase within a short time after DNA damage in G2 phase. **Supplementary Figure 14.** proteomic analysis of A549 cells after combination treatment. **Supplementary Figure 15.** The expression of SHCBP1 in mouse subcutaneous tumor tissues throughout the modeling period.

**Additional file 3: Supplementary video 1.** H1299 cells were transfected with control or SHCBP1 siRNA for 24 hours and then exposed to 5 μM ETOP for real-time recording. The rounded cells in the real-time video indicate mitotic cells. **Supplementary video 2.** Hela cells were transfected with control or SHCBP1 siRNA for 24 hours and then exposed to 3 μM ETOP for real-time recording. The rounded cells in the real-time video indicate mitotic cells.

## Data Availability

The datasets used and/or analyzed during the current study are available from the corresponding author on reasonable request.
