# Supplementary material for: The dynamic role of nucleoprotein SHCBP1 in the cancer cell cycle and its potential as a synergistic target for DNA-damaging agents in cancer therapy
Source: Cell Commun Signal. 2024 Feb 16;22:131. doi: 10.1186/s12964-024-01513-0 (PMC10874017; doi:10.1186/s12964-024-01513-0)
Supplement: Supplementary file 1 — Additional file 1: Table S1. Clinical characteristics of LUAD patients with different SHCBP1 expression from the TCGA dataset. Table S2. Clinical characteristics of NSCLC patients with different SHCBP1 expression from the Wuhan Union Hospital. Table S3. Clinical characteristics of LUAD patients with different SHCBP1 expression from the tissue microarray. Table S4. The primer sequences of the target genes for real‐time PCR. [file 12964_2024_1513_MOESM1_ESM.docx]

**Supplementary methods:**

**Bioinformatics analysis of online public databases**

SHCBP1 gene expression in normal and cancer tissues was analyzed using Oncomine cancer microarray database ([www.oncomine.org](http://www.oncomine.org) ) across various cancer subtypes using a cancer versus normal differential analysis. The different expression of SHCBP1 levels between a human patient’s tumor and the normal tissues across the TCGA database was analyzed by the TIMER (<https://cistrome.shinyapps.io/timer/>). Clinical data for 504 NSCLC samples, who had RNA-seq data (SHCBP1) and clinical stage data, were downloaded from TCGA database (<https://portal.gdc.cancer.gov/>) for statistical analysis. Meanwhile, the genes significantly related to SHCBP1 were analyzed for GO function and KEGG pathway enrichment. Survival was analyzed using Kaplan-Meier survival analysis.

**Immunohistochemical staining and scoring of SHCBP1 and Ki67 in human NSCLC tissues**

The human NSCLC tissues from Wuhan Union Hospital (**see details in supplementary methods**) and the purchased tissue microarray were subjected to the immunohistochemical (IHC) staining of SHCBP1 and Ki67 following the standard protocol, which was performed with horseradish peroxidase (HRP) conjugates using Diaminobenzidine (DAB) detection. Briefly, paraffin embedded tissue sections were dewaxed and subjected to antigen retrieval using Tris-EDTA buffer [10 mM tris base, 1 mM EDTA solution, and 0.05% Tween 20 (pH 9.0)]. The sections were incubated in 0.3% hydrogen peroxide in methanol for 30 min at room temperature to block the endogenous peroxidase activity, permeabilized with PBS containing 0.5% Triton-X100 and blocked in PBS containing 3% BSA, then stained with primary antibodies against SHCBP1 (Sigma Aldrich; HPA048876, 1:150) or Ki67 (Abcam, ab16667, 1:200) overnight at 4°C. After incubated with the HRP-conjugated secondary antibodies, IHC slides were visualized with DAB (Proteintech, # PK10006) and counterstained with hematoxylin. The IHC images were acquired by the NIKON Biological Microscope (Ni-E, Japan), and the scoring of IHC staining of SHCBP1 and Ki67 was performed by two observers in a blinded manner. The criteria were listed as follows: a minimum of 3 fields was examined and at least 500 cells were counted in each slide. The proportion of positive cells was classified into one of five categories (0: 0%–5%, 1: 5%–25%, 2: 26%–50%, 3: 51%–75%, or 4: 75%–100%). The staining intensity was classified as 0 (negative), 1 (weak), 2 (moderate), or 3 (strong). Finally, both positive proportion and intensity scores were multiplied to obtain the total IHC staining scores of SHCBP1. The Ki67 index was defined as the percentage of Ki67‐positive nuclei per 100 tumor cells. Staining was classified into 5 grades (0-5%, 5-25%, 25-50%, 50-75%, >75%). The IHC staining scores were used for subsequent survival and correlation analyses.

**Lentivirus package, infection and cell selection**

Lentivirus-carrying luciferase was constructed into a Ubi-MCS-Luc-IRES-Puromycin plasmid stably expressing luciferase, and lentiviruses to express SHCBP1 shRNA was constructed into a hU6-MCS-CMV-EGFP plasmid. These viruses were generated by co-transfecting the desired plasmid and the helper plasmids (pCMV-VSVG, pCMV-dR8.2 dvpr) into HEK293T cells. Viruses were collected 48 and 72 h after transfection through PEG8000 precipitation, mixed with fresh medium and added to the cancer cells together with 8 mg/ml of polybrene for 24 hr. Cells were replaced with fresh medium and cultured for another 48 hours, then 1.5 μg/ml puromycin was added to the media until total selection.

**Preparation of Cytoplasmic and Nuclear Fractions**

Treated cells were washed and scraped with cold PBS. Part of the cells were directly lysed to extract total protein (T) and stored at –80°C. The others were then centrifuged (4℃, 800 ×g, 5 min) and resuspended in cytoplasmic extraction buffer (10 mM HEPES [pH 7.5], 40 mM KCl, 2 mM MgCl2, 10% glycerol). The cells were then properly homogenized and centrifuged at 4000rpm for 5 min (4ºC). The supernatant was centrifuged again at 13000rpm for 15 min (4ºC) to obtain the second supernatant (cytoplasmic fraction) which was stored at –80°C until use. The nuclei in the pellet were isolated by gradient centrifugation with sucrose solution (0.25 M and 0.35 M sucrose buffer) and resuspended in nuclear extraction buffer (10 mM HEPES [pH 7.5], 500 mM NaCl, 1% Triton X-100, 10% glycerol) for 20 min at 4°C. After sonication and centrifugation at 15,000 × g for 15 min at 4ºC, the supernatant (nuclear fraction) was stored at –80°C. At last, total, cytoplasmic and nuclear fractions were subjected to western blotting analysis. GAPDH and Lamin B1 served as loading controls for cytoplasmic and nuclear fractions, respectively.

**Co-Immunoprecipitation**

To detect endogenous interaction between SHCBP1, RACGAP1, MKLP1 and PLK1, tumor cells growing in 10 cm dish were lysed in 1 ml NP-40 buffer (10 mM HEPES, pH 7.5, 142.5 mM KCl, 5 mM MgCl_2_, 1 mM EDTA, 1% NP-40, 5mM sodium pyrophosphate, 50 mM NaF, 1 mM Na_3_VO_4_) with PMSF and complete protease inhibitor cocktail (MCE) added. Following centrifugation at 12,000g at 4°C for 5 min, supernatants were collected and protein concentrations were determined using the BCA protein quantification Kit (Thermofisher, #23227). Take lysates containing 0.5-2 mg protein for each IP (depends on the protein abundance) and save some lysate at –80°C for immunoblotting controls. Briefly, lysates (1 mg protein) were diluted with NP-40 buffer to 500-800 μl and incubated with 30 μl protein A/G agarose beads (Millipore, #IP05) and 5 μg primary antibody overnight at 4°C with vertical rotation. Then, the immunoprecipitates were washed gently in PBS buffer for two times and resuspended in Tris-SDS buffer (50 mM Tris-HCl, pH 7.5, 150 mM NaCl, 1% SDS, 10% glycerin, containing protease inhibitors), boiled at 95°C for 5 min, and chilled on ice, followed by centrifugation at 16,200 x g for 5 min. The supernatants were then mixed with β- Mercaptoethanol and bromophenol blue for subsequent Western blotting.

**Western blotting**

Frozen tissue specimens were ground under liquid nitrogen for protein extraction. Cells were plated on 12-well or 6-well plates, treated as indicated. Whole cell extracts were prepared using western lysis buffer (50 mM Tris-HCl, pH 8.0, 150 mM NaCl, 1 mM EDTA, pH 8.0, 0.1% SDS, 1% NP-40, 5mM sodium pyrophosphate, 50 mM NaF, 1 mM Na_3_VO_4_, 5 mM dithiothreitol) with PMSF and complete protease inhibitor cocktail (MCE) added. Lysates were added with 4×SDS loading buffer (200mM Tris-base, 8% SDS, 40% glycerin, pH 6.8) and boiled. Protein concentration was determined by the BCA method as described above. Lysates containing 5-30 µg protein (mixed with β- Mercaptoethanol and bromophenol blue) were loaded to run on SDS-PAGE gels and transferred onto nitrocellulose membranes (whatman, # 10401196). Membranes were blocked in 3% w/v milk in TBST (50mM Tris, 150mM NaCl, 0.1% TWEEN, pH 7.6-8.0) and then incubated with antibodies at 1:1000 dilution in 3% BSA overnight at 4°C. Membranes were washed three times in TBST, then incubated with 1:10,000 secondary antibody (IRDye® 800CW anti-Rabbit or mouse IgG (H+L), LI-COR Biosciences) dilution in 3% BSA. Membranes were again washed three times in TBST and then signals were visualized and analyzed using Odyssey CLX (LI-COR, USA). Membranes were stripped using Restore™ Western Blot Stripping Buffer (Thermofisher) for sequential detections.

**FACS staining and analysis and cell cycle analysis**

For flow cytometry analysis of apoptosis, 1.0–5.0 × 10^6^ cultured cells were collected by trypsin digestion without EDTA and washed twice by cold PBS. After centrifuged at 800 rpm (4 °C) for 5 min, cells were resuspended in 200 μL 1ⅹAnnexin V Binding Buffer (BD pharmingen, #556454) and stained under dark with 2μl APC -conjugated Annexin V antibody (BD pharmingen, #550474) for 30 min. After being washed twice, cells were resuspended in 200 μL of 1ⅹAnnexin V Binding Buffer again and added with 5 μL 7-AAD (BD pharmingen, #559925) to incubate for 15-20min, which were finally examined by BD LSRFortessa X-20 flow cytometer (San Diego,CA).

For flow cytometry analysis of cell cycle, trypsinized cells were fixed overnight in 70% ethanol at -20 °C and washed out by the staining buffer (PBS containing 0.2% FBS), then stained with anti-phospho-histone H3 (PHH3) antibody (Alexa Fluor® 488 Conjugate, Cell Signaling Technology, 1:100) at 4°C for 30 min. DNA was subsequently stained with 40 μg/ml propidium iodide solution 15-20min. Cells were finally examined by the flow cytometer. All the acquired flow cytometry data were analyzed by the flowjo 10.0 or Modfit LT3.1software. To dynamically study the process of tumor cell cycle progression, we blocked A549, NCI-H1299 and HeLa cells at G1/S or G2/M phase by double thymidine or RO3306 block as previously mentioned, followed by a release into fresh medium for various time periods and then subjected to the above cell cycle analysis.

**Cell proliferation assays**

The cell growth was estimated by Live cell imaging counting (Celigo imaging cytometer, Nexcelcom), Cell Counting Kit-8 (CCK8) assay, or EdU incorporation assay (Thermo Fisher Scientific) according to the manufacturer’s instruction. Tumor cells were infected with shCtrl or shSHCBP1 lentivirus (expressing green fluorescent protein [GFP]) for 48-72 hours or otherwise indicated, and then subjected to the above assays.

Infected A549 and HeLa cells (1000-1500 cells / well) were seeded into 96-well plates with each well containing 100μl culture medium, and with 5-8 replicate wells per group per plate. Infected cells expressing GFP were visualized and quantified using the Celigo (Nexcelcom) imaging cytometer for 5 consecutive days. For CCK8 assay, 10 μL of CCK8 solution was added to each well of different plate at 1st, 2nd, 3rd, 4th, and 5th day, respectively, and incubated for 30min-2h at 37°C. OD values of each plate were then measured at 450 nm at the same time each day. The growth curve was drawn according to the daily cell number or OD value to evaluate the cell proliferation. To evaluate the cell viability after drug treatment, cells were transfected with control or SHCBP1 siRNA for 24 hours and then seeded into the 96-well plate. After 12 hours incubation at 37 °C with 5% CO_2_, cells were then subjected to 0.1μM docetaxel (DTX) or low-dose etoposide (ETOP: 1μM for A549, 3μM for HeLa, and 5μM for H1299 cells) for another 24 hours. Then, the CCK8 assay was performed.

For EdU incorporation assay, infected cells were seeded into 24-well plates and incubated at 37 °C with 5% CO_2_ for 12 h, then subjected to 20μM EdU (5-ethynyl-2′-deoxyuridine) incubation (Click-iT™ Plus EdU Alexa Fluor™ 555 Imaging Kit, #C10638) for 2 hours. Cells were fixed and permeabilized, EdU was detected according to the manufacturer's directions, and nuclei were stained with Hoechst.

**Liu staining**

Cells in the well plate were washed with PBS and quickly dried in the air, and then an appropriate amount of Liu A solution (Baso Biotech, China) was added (to ensure that the solution covered all cells at the bottom), staining for about 1 to 2 minutes at room temperature, after that, add twice the volume of Liu B solution, mix solution A and B quickly and thoroughly with ear-washing ball, shake gently on the shaker for about 4-6 minutes. Finally, rinse the well plate under slow running water until the gap between the plates is transparent. After the well plate is naturally dried, it can be photographed under a microscope.

**Proteomic Analysis**

For sample preparation, four sets of cell samples were prepared, each with two biological replicates. Briefly, NCI-H1299 cells were transfected with control or SHCBP1 siRNA for 24 h, followed by exposure to 5μM etoposide or corresponding vehicle (DMSO) for 24 h. Cells were washed and scraped off in 500 μl cold PBS on ice, then lysed in 8 mol/L urea buffer (containing 1% protease inhibitor, 3 μM TSA, 50 mM NAM and 2 mM EDTA) and ultrasonized. The protein concentration was acquired by Biotek Synergy 2 microplate reader. After the protein digestion by trypsin, peptide was desalted by Strata X C18 SPE column (Phenomenex) and vacuum-dried, labeled with tandem mass tag (TMT), and separated by following the instruction for LC‐MS/MS system. By using data‐dependent acquisition mode, a data-dependent procedure that alternated between one MS scan followed by 20 MS/MS scans with 15.0s dynamic exclusion were carried out with full scans in Orbitrap mass analyzer. The resulting MS/MS data were processed using Maxquant search engine (v.1.5.2.8). Tandem mass spectra were searched against human uniprot database concatenated with reverse decoy database. The searching parameters were set to the modified default value.

The protein was quantified by taking the median of the corresponding specific peptide quantification values. For each replicate experiment, the ratio of protein quantification values between two different samples (fold change) was taken as the differential expression of the comparison group. For each comparison group, we used the average fold change value and the coefficient variation (CV) of fold change values of two repeated experiments to identify the differentially expressed proteins (DEPs, when CV-value < 0.2 and fold change >1.25 or <0.80). The MS identified information and analysis between each comparison group were listed in **Supplementary materials**.

Proteins were classified by GO annotation into three categories: biological process (BP), cellular compartment (CC) and molecular function (MF). Encyclopedia of Genes and Genomes (KEGG) database was used to identify enriched pathways. Two-tailed Fisher’s exact tests were employed to test the enrichment of the DEPs against all identified proteins. A corrected p-value < 0.05 was considered significant. For identification of evolutionary homologs (orthologous and paralogous) of DEPs, we performed euKaryotic Conserved Orthologous Groups (KOG) analysis. Furthermore, to cluster DEPs according to the expression patterns of the four groups of samples that received different treatments, we then applied fuzzy c-means clustering (k = 6, m = 2) to all DEPs by using the R package Mfuzz.

**Supplementary tables:**

**Key resources Table**

| **REAGENT or RESOURCE** | **SOURCE** | **IDENTIFIER** |
| --- | --- | --- |
| **Antibodies** | | |
| SHCBP1 | Proteintech | Cat# 12672-1-AP; RRID: AB_10639526 |
| SHCBP1 | Sigma Aldrich | Cat# HPA048876; RRID: AB_2680542 |
| SHCBP1(EPR18843) | Abcam | Cat# ab184467 |
| Ki67(SP6) | Abcam | Cat# ab16667; RRID: AB_302459 |
| alpha Tubulin | Abcam | Cat# ab18251; RRID: AB_2210057 |
| alpha Tubulin (clone DM1A) | Cell Signaling Technology | Cat# 3873S; RRID: AB_1904178 |
| γ-Tubulin | Sigma Aldrich | Cat# T6557; RRID: AB_477584 |
| B23/NPM1 | Proteintech | Cat# 60096-1-Ig; RRID: AB_2155162 |
| RACGAP1/MGCRACGAP(5G5) | Abcam | Cat# ab219112 |
| MKLP1 | Abcam | Cat# ab9259; RRID: AB_307118 |
| PLK1(35-206) | Abcam | Cat# ab17056; RRID: AB_443612 |
| Cyclin B1(Y106) | Abcam | Cat# ab32053; RRID: AB_731779 |
| Cyclin E1 | Proteintech | Cat# 11554-1-AP; RRID: AB_2071066 |
| Rabbit polyclonal anti-Phospho-cdc25C (Thr48) | Cell Signaling Technology | Cat# 9527; RRID: AB_331486 |
| Rabbit monoclonal anti-Phospho-cdc25C (Ser216) (63F9) | Cell Signaling Technology | Cat# 4901; RRID: AB_331215 |
| Phospho-CDK Substrate (p TPXK) (D9V5N) | Cell Signaling Technology | Cat# 14371S; RRID: AB_2798466 |
| Phospho-Histone H3 (Ser10) (D2C8) XP® Rabbit mAb (Alexa Fluor® 488 Conjugate) | Cell Signaling Technology | Cat# 3465S; RRID: AB_10695860 |
| Phospho-Histone H3 (Ser10) (6G3) | Cell Signaling Technology | Cat# 9706S; RRID: AB_331748 |
| Phospho-cdc2 (Tyr15) | Cell Signaling Technology | Cat# 9111; RRID: AB_331460 |
| Wee1 (D10D2) Rabbit mAb | Cell Signaling Technology | Cat# 13084; RRID:AB_2713924 |
| NEK7(EPR4900) | Abcam | Cat# ab133514; RRID: AB_2877625 |
| ZW10 | Proteintech | Cat# 24561-1-AP; RRID: AB_2815001 |
| gamma H2A.X (phosphor S139) (9F3) | Abcam | Cat# ab26350; RRID: AB_470861 |
| P53 | Proteintech | Cat# 10442-1-AP; RRID: AB_2206609 |
| Phospho-Chk1 (Ser345) (133D3) | Cell Signaling Technology | Cat# 2348T; RRID: AB_331212 |
| Phospho-Chk2 (Thr68) | Cell Signaling Technology | Cat# 2661; RRID: AB_331479 |
| DDB2 (D4C4) | Cell Signaling Technology | Cat# 5416; RRID: AB_10694497 |
| XLF(NHEJ1) | Proteintech | Cat# 11888-1-AP; RRID: AB_2282851 |
| RRM2 | Proteintech | Cat# 11661-1-AP; RRID: AB_2180392 |
| GAPDH | Proteintech | Cat# 60004-1-Ig; RRID: AB_2107436 |
| Lamin B1 | Proteintech | Cat# 12987-1-AP; RRID: AB_2136290 |
| beta Actin | Proteintech | Cat# 60008-1-Ig; RRID: AB_2289225 |
| IRDye® 800CW Goat anti-Rabbit IgG (H+L) | LI-COR Biosciences | Cat# 926-32211; RRID: AB_621843 |
| IRDye® 800CW Goat anti-Mouse IgG (H+L) | LI-COR Biosciences | Cat# 926-32210; RRID: AB_621842 |
| Donkey anti-Mouse IgG (H+L) Highly Cross-Adsorbed Secondary Antibody, Alexa Fluor 488 | Thermo Fisher Scientific | Cat# A-21202; RRID: AB_141607 |
| Donkey anti-Rabbit IgG (H+L) Highly Cross-Adsorbed Secondary Antibody, Alexa Fluor 555 | Thermo Fisher Scientific | Cat# A-31572; RRID: AB_162543 |
| Donkey anti-Rabbit IgG (H+L) Highly Cross-Adsorbed Secondary Antibody, Alexa Fluor 488 | Thermo Fisher Scientific | Cat# A-21206; RRID: AB_141708 |
| Goat anti-Mouse IgG (H+L) Highly Cross-Adsorbed Secondary Antibody, Alexa Fluor 546 | Thermo Fisher Scientific | Cat# A-11030; RRID: AB_144695 |
| **Bacterial and Virus Strains** | | |
| Plasmid hU6-MCS-CMV-EGFP | Genechem | Cat# GV115 |
| Plasmid hU6 MCS CMV Puromycin | Genechem | Cat# GV112 |
| Plasmid Ubi-MCS-Luc-IRES-Puromycin | Genechem | Cat# GV260 |
| pCMV-VSV-G | Addgene | Cat# 8454 |
| pCMV-dR8.2 dvpr | Addgene | Cat# 8455 |
| **Biological Samples** |  |  |
| Human lung cancer tissues and malignant pleural effusion | This paper, Wuhan union hospital | NA |
| LUAD tissue microarray | Shanghai Outdo Biotech Co., Ltd | Cat# HLugA180Su02 |
| **Chemicals, Peptides, and Recombinant Proteins** | | |
| Etoposide | Selleck | Cat# S1225 |
| Cisplatin | Selleck | Cat# S1166 |
| Docetaxel | Selleck | Cat# S1148 |
| Paclitaxel | Selleck | Cat# S1150 |
| Nocodazole (R17934) | Selleck | Cat# S2775 |
| RO3306 | Selleck | Cat# S7747 |
| Thymidine | Sigma Aldrich | Cat# T1895 |
| D-Luciferin, Potassium Salt (Proven and Published™) | Goldbio | Cat# 115144-35-9 |
| Puromycin | Sigma-Aldrich | Cat# P9620 |
| Hoechst 33342 | Thermo Fisher Scientific | Cat# H1399 |
| **Critical Commercial Assays** | | |
| Immunohistochemistry kit (KIHC-5) | Proteintech | Cat# PK10006 |
| Click-iT™ Plus EdU Alexa Fluor™ 555 Imaging Kit | Thermo Fisher Scientific | Cat# C10638 |
| Click-iT™ EdU Alexa Fluor™ 647 kit | Thermo Fisher Scientific | Cat# C10424 |
| Senescence β-Galactosidase Staining Kit | Cell Signaling Technology | Cat# 9860S |
| CCK8 assay kit | Beyotime | Cat# C0042 |
| Lipofectamine™ 2000 Transfection Reagent | Thermo Fisher Scientific | Cat# 11668019 |
| Lipofectamine™ RNAiMAX Transfection Reagent | Thermo Fisher Scientific | Cat# 13778150 |
| RNAiso Plus (Trizol) | Takara Bio | Cat# 9109 |
| cDNA synthesis Kit | TOYOBO | Cat# FSQ-201 |
| All in One qPCR RT kit | Genecopoeia | Cat#AOPR-0600 |
| PI/RNase Staining Buffer Solution | BD Pharmingen | Cat# 550825 |
| Annexin V Binding Buffer 10X 50mL | BD Pharmingen | Cat# 556454 |
| 7-AAD Staining Solution 2mL | BD Pharmingen | Cat# 559925 |
| ANNEXIN V RECOM APC 100TST | BD Pharmingen | Cat# 550474 |
| Annexin V binding buffer | BD Pharmingen | Cat# 556454 |
| Protein G Plus/Protein A Agarose Suspension | Millipore | Cat# IP05 |
| BCA Protein Quantitation Kit | Thermo Fisher Scientific | Cat# 23227 |
| Protease inhibitor Cocktail (EDTA-Free,100× in DMSO) | MCE | Cat# HY-K0010 |
| Protein Ladder | Thermo Fisher Scientific | Cat# 26616 |
| **Deposited Data** | | |
| Proteomic data | This paper, supplementary materials | mass spectrometry identified information (excel) |
| **Experimental Models: Cell Lines** | | |
| HBE | ATCC | Cat# CRL-2078 |
| A549 | ATCC | Cat# CCL-185 |
| NCI-H1299 | ATCC | Cat# CRL-5803 |
| NCI-H460 | ATCC | Cat# HTB-177 |
| HeLa | ATCC | Cat# CCL-2 |
| NCI-H292 | ATCC | Cat# CRL-1848 |
| LLC | ATCC | Cat# CRL-1642 |
| HEK293T | ATCC | Cat# CRL-3216 |
| **Experimental Models: Organisms/Strains** | | |
| C57BL/6 wild type mice | Changzhou Cavens Laboratory Animal Ltd | NA |
| BALB/c nude mice | Changzhou Cavens Laboratory Animal Ltd | NA |
| **Oligonucleotides** | | |
| Human siSHCBP1-1 (siRNA1)  CCATAGTGATCCATTGTCT | This paper | NA |
| Human siSHCBP1-2 (siRNA2)  GCAGTGATTGTAGCTACCGTGATAA | This paper | NA |
| Human siSHCBP1-3 (siRNA3)  GCGATTCAGAGCCTATCAA | This paper | NA |
| Human siSHCBP1-4 (siRNA4)  GCTGAAACTCATTGAGAAT | This paper | NA |
| Human shSHCBP1  TGGTGAAACCTACAATCTT | This paper | NA |
| Mouse shSHCBP1  CACCAGATGGCACTTGCAATT | This paper | NA |
| Human siWEE1-1 (siRNA1)  GGAAAAAGGGAAUUUGAUG | Beck H, Nähse V, Larsen MS, Groth P, Clancy T, Lees M, et al. Regulators of cyclin-dependent kinases are crucial for maintaining genome integrity in S phase. The Journal of cell biology. 2010;188(5):629-38. |  |
| Human siWEE1-2 (siRNA2)  GGGAAUUUGAUGUGCGACA |  |  |
| Human siWEE1-3 (siRNA3)  GGUAUAUUCAUUCAAUGUC |  |  |

**Table S1. Clinical characteristics of LUAD patients with different SHCBP1 expression from the TCGA dataset**

| **Characteristics** |  | **SHCBP1 expression (N = 504)** | | ***P* value** |
| --- | --- | --- | --- | --- |
|  |  | **Low (N = 252)** | **High (N = 252)** |  |
| **Age, years** | Median (IQR) | 67.0 (60.0-74.0) | 65.0 (58.0-72.0) | 0.084 |
| **Sex** | Male, n (%) | 112 (44.4%) | 121 (48.0%) | 0.421 |
|  | Female, n (%) | 140 (55.6%) | 131 (52.0%) |  |
| **T** | T1, n/N (%) | 99/251 (39.4%) | 67/250 (26.8%) | 0.02 |
|  | T2, n/N (%) | 120/251 (47.8%) | 151/250 (60.4%) |  |
|  | T3, n/N (%) | 23/251 (9.2%) | 22/250 (8.8%) |  |
|  | T4, n/N (%) | 9/251 (3.6%) | 10/250 (4.0%) |  |
| **N** | N0, n/N (%) | 174/246 (70.7%) | 150/247 (60.7%) | 0.019 |
|  | N1/2/3, n/N (%) | 72/246 (29.3%) | 97/247 (39.3%) |  |
| **M** | M0, n/N (%) | 166/172 (96.5%) | 171/188 (91.0%) | 0.031 |
|  | M1, n/N (%) | 6/172 (3.5%) | 17/188 (9.0%) |  |
| **Clinical tumor staging** | I, n/N (%) | 152/250 (60.8%) | 120/248 (48.4%) | 0.019 |
|  | II, n/N (%) | 54/250 (21.6%) | 65/248 (26.2%) |  |
|  | III, n/N (%) | 37/250 (14.8%) | 46/248 (18.5%) |  |
|  | IV, n/N (%) | 7/250 (2.8%) | 17/248 (6.9%) |  |

**Note:** Data were presented as median (IQR) for continuous variables and n/N (%) for category variables. Mann-Whitney U test was used for analysis of continuous variables and χ² test for analysis of all category variables between two groups. All tests were two-tailed, and a p value less than 0.05 was deemed statistically significant. Tumor T, N, M and pathological staging were evaluated in accordance with the AJCC 7th Edition Cancer Staging guideline.

**Abbreviations:** LUAD = Lung adenocarcinoma, IQR = interquartile range.

**Table S2. Clinical characteristics of NSCLC patients with different SHCBP1 expression from the Wuhan Union Hospital.**

| **Characteristics** |  | **SHCBP1 expression (N = 213)** | | ***P* value** |
| --- | --- | --- | --- | --- |
|  |  | **Low (N = 83)**  **(IHCss < 12)** | **High (N = 130)**  **(IHCss = 12)** |  |
| **Age, years** | >65, n (%) | 23 (27.7%) | 33 (25.4%) | 0.707 |
|  | ≤65, n (%) | 60 (72.3%) | 97 (74.6%) |  |
| **Sex** | Male, n (%) | 48 (57.8%) | 90 (69.2%) | 0.089 |
|  | Female, n (%) | 35 (42.2%) | 40 (30.8%) |  |
| **Histopathologic type** | LUAD, n (%) | 63 (75.9%) | 93 (71.5%) | 0.483 |
|  | SCC, n (%) | 20 (24.1%) | 37 (28.5%) |  |
| **Smoking History** | Yes, n (%) | 43 (51.8%) | 68 (52.3%) | 0.943 |
|  | No, n (%) | 40 (48.2%) | 62 (47.7%) |  |
| **T** | T1, n (%) | 35 (42.2%) | 31 (23.8%) | 0.027 |
|  | T2, n (%) | 30 (36.1%) | 60 (46.2%) |  |
|  | T3, n (%) | 13 (15.7%) | 22 (16.9%) |  |
|  | T4, n (%) | 5 (6.0%) | 17 (13.1%) |  |
| **N** | N0, n/N (%) | 60/81 (74.1%) | 62/122 (50.8%) | 0.001 |
|  | N1/2/3, n/N (%) | 21/81 (25.9%) | 60/122 (49.2%) |  |
| **M** | M0, n/N (%) | 76/82 (92.7%) | 112/125 (89.6%) | 0.452 |
|  | M1, n/N (%) | 6/82 (7.3%) | 13/125 (10.4%) |  |
| **Clinical tumor staging** | I, n/N (%) | 41/81 (50.6%) | 38/119 (31.9%) | 0.005 |
|  | II, n/N (%) | 18/81 (22.2%) | 19/119 (16.0%) |  |
|  | III, n/N (%) | 16/81 (19.8%) | 50/119 (42.0%) |  |
|  | IV, n/N (%) | 6/81 (7.4%) | 12/119 (10.1%) |  |
| **Ki67 positive index** | 0-5%, n/N (%) | 22/75 (29.3%) | 13/122 (10.7%) | 0.011 |
|  | 5-25%, n/N (%) | 18/75 (24.0%) | 29/122 (23.8%) |  |
|  | 25-50%, n/N (%) | 15/75 (20.0%) | 26/122 (21.3%) |  |
|  | 50-75%, n/N (%) | 13/75 (17.3%) | 36/122 (29.5%) |  |
|  | >75%, n/N(%) | 7/75 (9.3%) | 18/122 (14.8%) |  |
| **EGFR mutation** | Yes, n/N (%) | 24/44 (54.5%) | 33/60 (55.0%) | 0.963 |
|  | No, n/N (%) | 20/44 (45.5%) | 27/60 (45.0%) |  |

**Note:** Data were presented as n/N (%), and χ² test was used for analysis of all category variables between two groups. All tests were two-tailed, a p value less than 0.05 was deemed statistically significant. Tumor T, N, M and pathological staging were evaluated in accordance with the International Association for the Study of Lung Cancer (IASLC) 8th edition TNM staging guideline.

**Abbreviations:** NSCLC = non-small cell lung cancer, IHCss **=** Immunohistochemical staining score, LUAD = Lung adenocarcinoma, SCC = squamous cell lung carcinoma.

**Table S3. Clinical characteristics of LUAD patients with different SHCBP1 expression from the tissue microarray.**

| **Characteristics** |  | **SHCBP1 expression (N = 92)** | | ***P* value** |
| --- | --- | --- | --- | --- |
|  |  | **Low (N = 56)**  **(IHCss < 12)** | **High (N = 36)**  **(IHCss = 12)** |  |
| **Age, years** | >65, n (%) | 20 (35.7%) | 13 (36.1%) | 0.969 |
|  | ≤65, n (%) | 36 (64.3%) | 23 (63.9%) |  |
| **Sex** | Male, n (%) | 31 (55.4%) | 18 (50%) | 0.615 |
|  | Female, n (%) | 25 (44.6%) | 18 (50%) |  |
| **T** | T1, n (%) | 11 (19.6%) | 7 (19.4%) | 0.897 |
|  | T2, n (%) | 33 (58.9%) | 19 (52.8%) |  |
|  | T3, n (%) | 9 (16.1%) | 8 (22.2%) |  |
|  | T4, n (%) | 3 (5.4%) | 2 (5.6%) |  |
| **N** | N0, n/N (%) | 24/44 (54.5%) | 14/31 (45.2%) | 0.423 |
|  | N1/2/3, n/N (%) | 20/44 (45.5%) | 17/31 (54.8%) |  |
| **M** | M0, n/N (%) | 56 (100%) | 34/35 (97.1%) | 0.385^*^ |
|  | M1, n/N (%) | 0 (0%) | 1/35 (2.9%) |  |
| **Clinical tumor staging** | I, n/N (%) | 19 (33.9%) | 8/35 (22.9%) | 0.44 |
|  | II, n/N (%) | 20 (35.7%) | 14/35 (40.0%) |  |
|  | III, n/N (%) | 17 (30.4%) | 12/35 (34.3%) |  |
|  | IV, n/N (%) | 0 (0%) | 1/35 (2.9%) |  |

**Note:** Data were presented as n/N (%), and χ² test or Fisher exact test was used for analysis of all category variables between two groups. All tests were two-tailed, a p value less than 0.05 was deemed statistically significant. Tumor T, N, M and clinical tumor staging were evaluated in accordance with the the AJCC 7th Edition Cancer Staging guideline.

^*^, Fisher exact test

**Abbreviations:** LUAD = Lung adenocarcinoma, IHCss **=** Immunohistochemical staining score

**Table S4. The primer sequences of the target genes for real‐time PCR**

| **Target gene** | **Primer sequences (5’-3’)** |
| --- | --- |
| Human SHCBP1 Forward | GCTACCGTGATAAACCAGGTTC |
| Human SHCBP1 Reverse | AGGCTCTGAATCGCTCATAGA |
| Human GAPDH Forward | TGACTTCAACAGCGACACCCA |
| Human GAPDH Reverse | CACCCTGTTGCTGTAGCCAAA |
| Human WEE1 Forward | AGGGAATTTGATGTGCGACAG |
| Human WEE1 Reverse | CTTCAAGCTCATAATCACTGGCT |
| Human MKi67 Forward | AATCTGTGCAGAGAGTAACG |
| Human MKi67 Reverse | CCCTATGACTTCTGGTTCTTAT |
| Human EPCAM Forward | AATCGTCAATGCCAGTGTACTT |
| Human EPCAM Reverse | TCTCATCGCAGTCAGGATCATAA |
| Human PCNA Forward | ATATCATTACACTAAGGGCCGA |
| Human PCNA Reverse | CTGGTTTGGTGCTTCAAATACT |
| Human NEK7 Forward | GCAGATGCTGGCGACCTATC |
| Human NEK7 Reverse | CCCAGTGGCTGTAATGAACAC |
| Human ZW10 Forward | AGCTGATTGTATGGAAGTTCCCA |
| Human ZW10 Reverse | TCTTTGTGCGATTGTTCAGTGT |
| Mouse SHCBP1-1 Forward | TTCACCATGGCAAGACCACA |
| Mouse SHCBP1-1 Reverse | TATACCAGCACCCTTGGCAC |
| Mouse GAPDH Forward | AATGTGTCCGTCGTGGATCTGA |
| Mouse GAPDH Reverse | GATGCCTGCTTCACCACCTTCT |
