# Supplementary material for: The dynamic role of nucleoprotein SHCBP1 in the cancer cell cycle and its potential as a synergistic target for DNA-damaging agents in cancer therapy
Source: Cell Commun Signal. 2024 Feb 16;22:131. doi: 10.1186/s12964-024-01513-0 (PMC10874017; doi:10.1186/s12964-024-01513-0)
Supplement: Supplementary file 2 — Additional file 2: Supplementary Figure 1. Expression of SHCBP1 in isolated lung adenocarcinoma cells of patients with malignant pleural effusion. Supplementary Figure 2. Expression of SHCBP1 in different LUAD and other cell lines. Supplementary Figure 3. SHCBP1 is highly consistent with the expression of Mitotic phase-associated proteins. Supplementary Figure 4. SHCBP1 expression changed with the cell cycle progression. Supplementary Figure 5. Knockdown efficiency of different small interfering RNAs of SHCBP1. Supplementary Figure 6. Representative DNA histograms (A) and statistical bar chart (B) obtained by flow cytometry of all cells at different time points after release from the TdR block. Supplementary Figure 7. SHCBP1 knockdown slows tumor cell cycle but promotes premature mitotic entry in Hela cells. Supplementary Figure 8. Effect of SHCBP1 knockdown on tumor cells entering and exiting M phase. Supplementary Figure 9. SHCBP1 knockdown inhibits tumor proliferation and metastasis in C57BL/6 mice. Supplementary Figure 10. Expression of SHCBP1 is elevated after treatment with DNA-damaging agents. Supplementary Figure 11. Cell cycle arrest after low-dose etoposide and cisplatin treatment. Supplementary Figure 12. CCK 8 assay in tumor cells after SHCBP1 knockdown combined with docetaxel (A) or etoposide exposure (B). Supplementary Figure 13. Tumor cells transfected with siCtrl or siSHCBP1 siRNA did not enter M phase within a short time after DNA damage in G2 phase. Supplementary Figure 14. proteomic analysis of A549 cells after combination treatment. Supplementary Figure 15. The expression of SHCBP1 in mouse subcutaneous tumor tissues throughout the modeling period. [file 12964_2024_1513_MOESM2_ESM.docx]

**Supplementary Figures:**

**
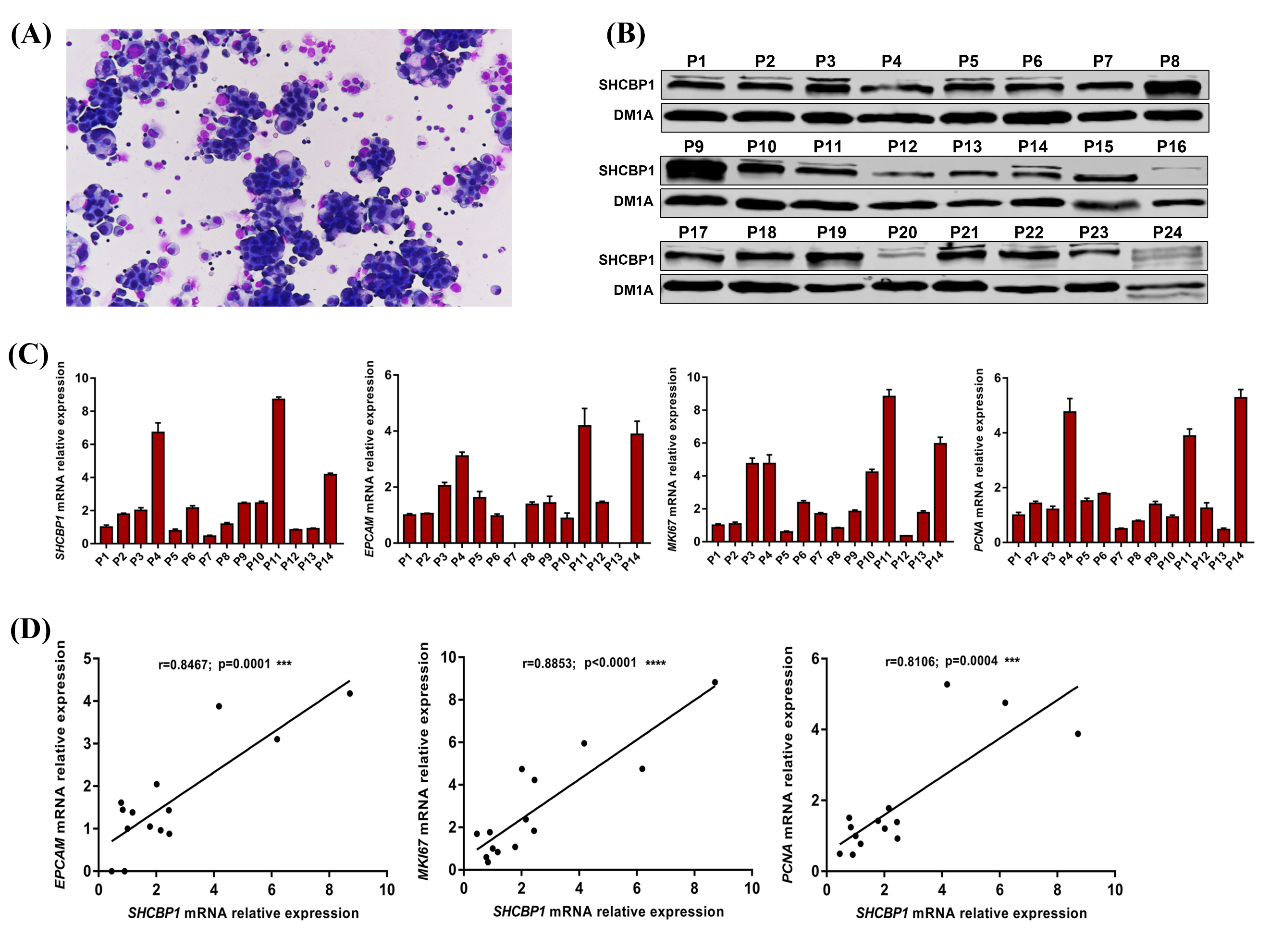
Supplementary Figure 1. Expression of SHCBP1 in isolated lung adenocarcinoma cells of patients with malignant pleural effusion**

**(A)** Representative image of lung adenocarcinoma cells in a patient with malignant pleural effusion.

**(B)** Western blot analysis of SHCBP1 in lung adenocarcinoma cells isolated from patients with malignant pleural effusion (n=24). DM1A (α-tubulin) was used as an internal reference.

**(C, D)** SHCBP1, EPCAM, MKI67 and PCNA mRNA expression relative to GAPDH in lung adenocarcinoma cells isolated from patients with malignant pleural effusion (n=17) **(C)** and their correlation analysis **(D)** are shown. Relative SHCBP1 mRNA expression was calculated as a fold-change versus the patient 1 (P1). Data are expressed as mean ± SD (Technical triplicates were performed for each sample). Pearson correlation analysis was performed to explore the correlation between SHCBP1 and EPCAM, MKI67 or PCNA.


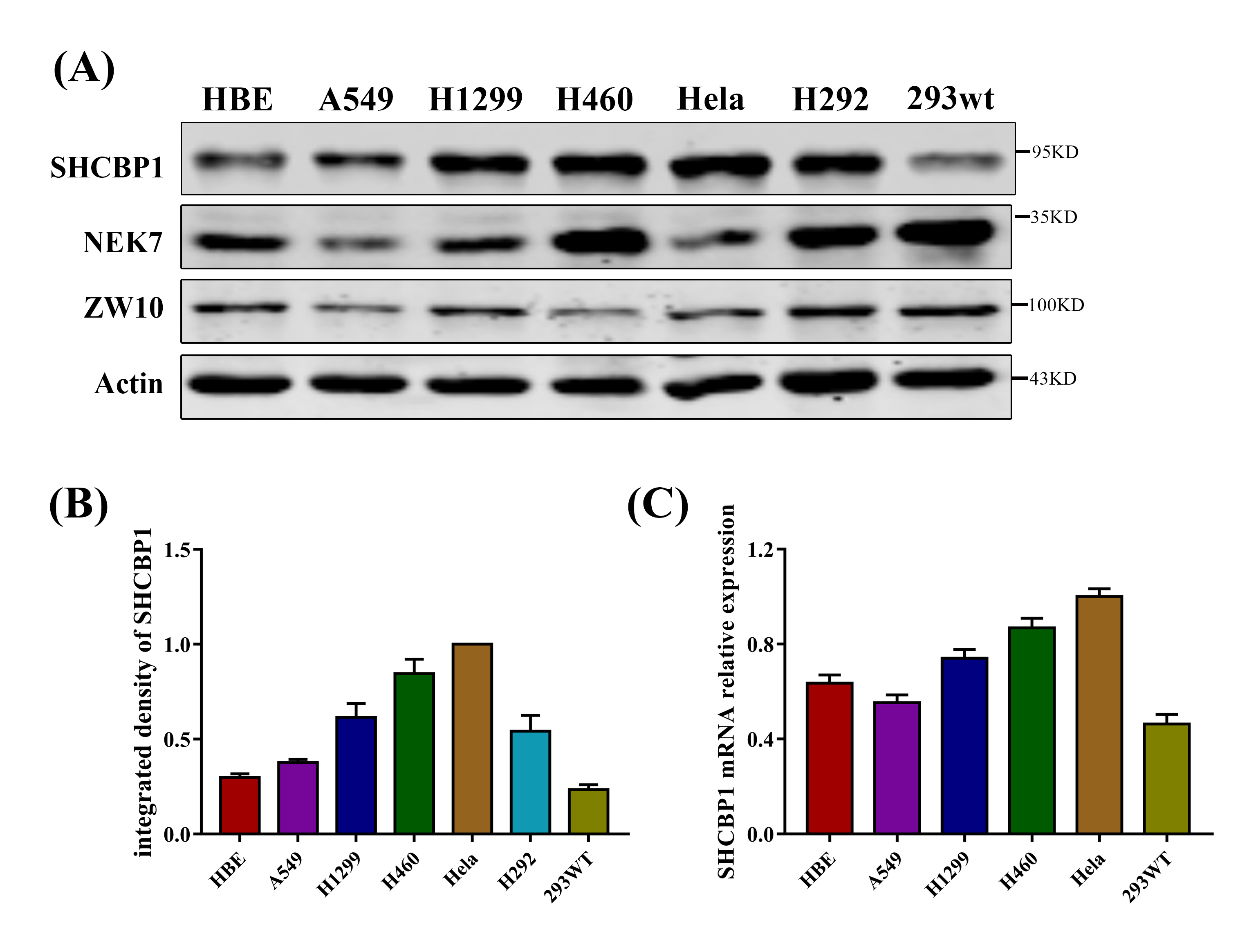


**Supplementary Figure 2. Expression of SHCBP1 in different LUAD and other cell lines**

**(A, B)** Western blot analysis of SHCBP1, NEK7 and ZW10 in seven cell lines, including immortalized human bronchial epithelial cells (HBE), NSCLC cells (A549, NCI-H1299, NCI-H460, and NCI-H292), HeLa and 293wt cells; actin was used as internal reference. **A**, representative western blot images. **B**, gray value quantification of western blot bands for SHCBP1. Results are shown as mean ± SD (n=3).

**(C)** SHCBP1 mRNA expression relative to GAPDH in the above cell lines. Results are shown as mean ± SD (n=3).

**
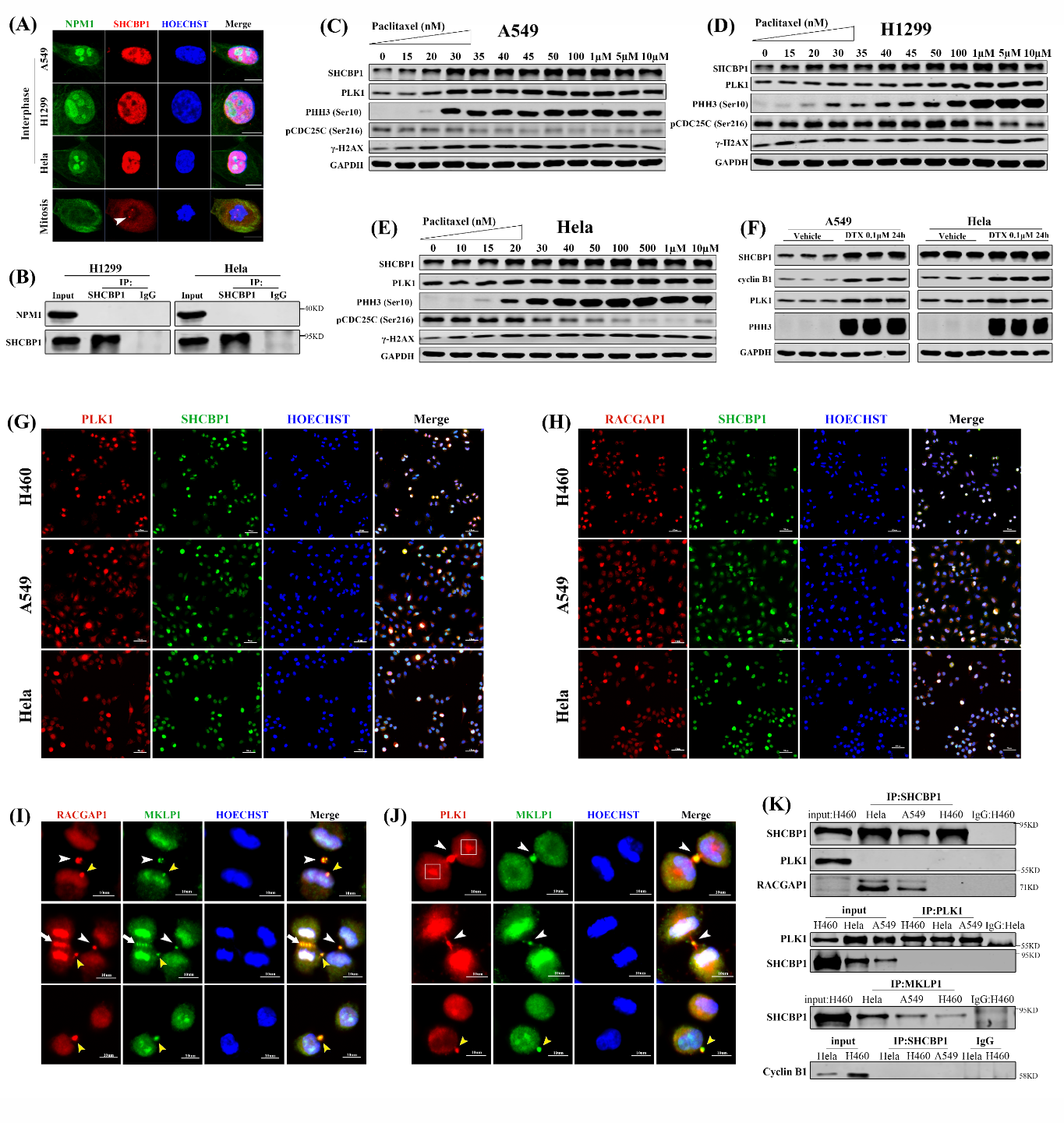
**

**Supplementary Figure 3. SHCBP1 is highly consistent with the expression of Mitotic phase-associated proteins**

**(A)** Immunofluorescence staining shows co-localization of SHCBP1 (red) and nucleophosmin (NPM1, nucleolar protein) (green) at the single-cell level during interphase and mitosis of A549, NCI-H1299 and HeLa cells. White arrows indicate spindle pole. Scale bars, 10μm.

**(B)** Co-immunoprecipitation assay of endogenous SHCBP1 and NPM1 in NCI-H1299 and HeLa cells.

**(C-E)** Western blot analysis of SHCBP1, PLK1, PHH3 (Ser10), phosphorylated cdc25C (Ser216), and γH2AX in A549 **(C)**, NCI-H1299 **(D)**, and HeLa cells **(E)** after treated with different concentration gradients of paclitaxel for 24 h. GAPDH was used as internal reference.

**(F)** Western blot analysis of SHCBP1, cyclin B1, PLK1 and PHH3 (Ser10) in A549 and HeLa cells after treated with 0.1μM docetaxel (DTX) for 24 h. GAPDH was used as internal reference.

**(G, H)** Representative immunofluorescence images showing highly consistent expression patterns of SHCBP1 and PLK1 **(G)** or RACGAP1 **(H)** in NCI-H460, A549 and HeLa cells. Cells were co-stained with anti-SHCBP1 (green), anti-PLK1(red) or RACGAP1(red) antibody and Hoechst (blue). Scale bars, 50μm.

**(I, J)** Immunofluorescence co-staining of MKLP1 (green) and RACGAP1 (red) **(I)** or PLK1 (red) **(J)** in NSCLC cells. White arrows indicate central spindle and the white-arrowheads indicate midbody. Scale bars, 10μm.

**(K)** Co-immunoprecipitation assay of endogenous SHCBP1 with PLK1, RACGAP1, and cyclin B1 in HeLa, A549 and NCI-H460 cells.


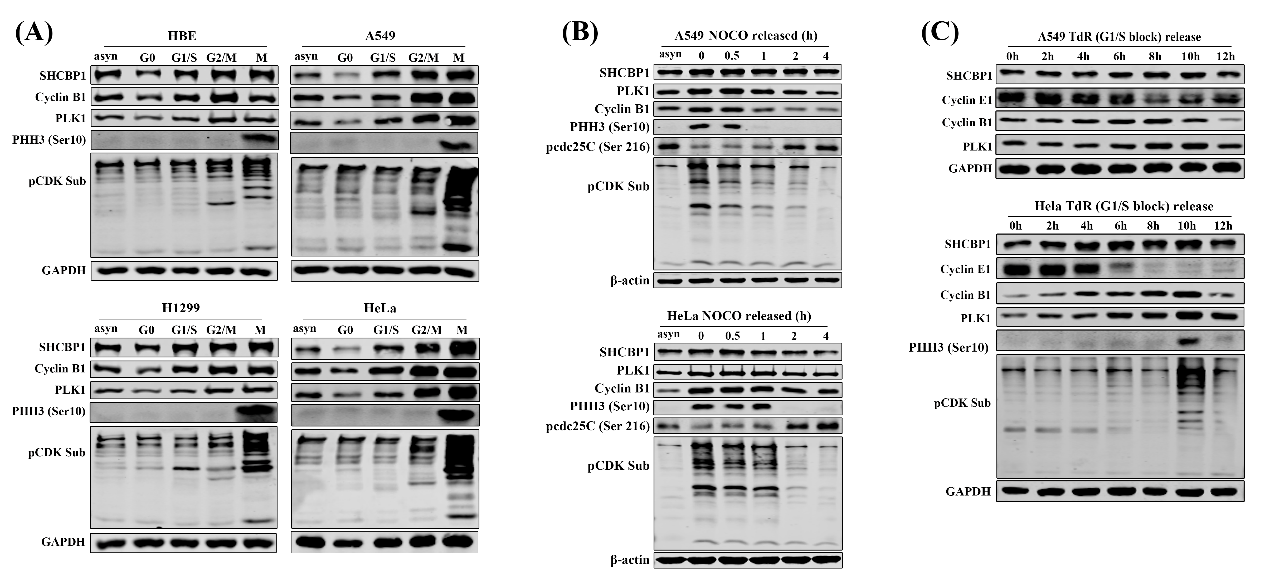


**Supplementary Figure 4. SHCBP1 expression changed with the cell cycle progression.**

**(A)** Representative western blots of SHCBP1 throughout the cell cycle of HBE, A549, H1299 and HeLa cells (cells were synchronized to G0, G1/S, and G2/M phase by serum starvation, double thymidine and RO3306 block, respectively).

**(B)** Representative western blot images of SHCBP1, PLK1, cyclin B1, PHH3 (Ser10), pcdc25C (Ser 216), and pCDK Sub in A549 and HeLa cells analyzed at indicated time points after nocodazole release.

**(C)** Representative Western blot images of SHCBP1, cyclin E1, cyclin B1, PLK1, PHH3 (Ser10) and pCDK Sub in A549 and HeLa cells analyzed at indicated time points after double thymidine (TdR) release.

Note: asyn, asynchronized. PLK1 and Cyclin B1, both well-known G2–M phase-associated proteins. pCDK Sub and PHH3 (Ser10) were used to indicate whether cells were in the late G2 or M phases, respectively. Cyclin E1 was used to indicate whether cells were in the G1–S phase.

**
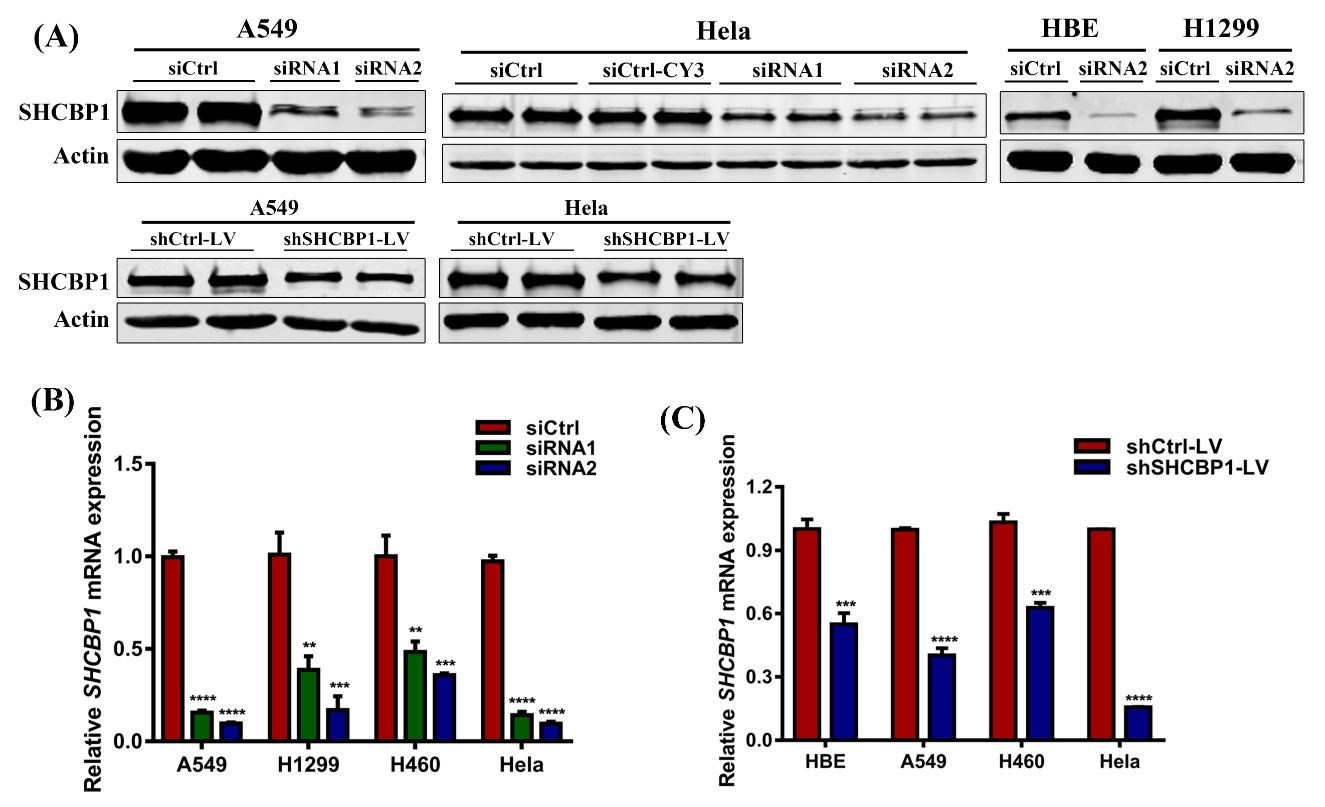
Supplementary Figure 5. Knockdown efficiency of different small interfering RNAs of SHCBP1**

The knockdown efficiency of three SHCBP1 interfering sequences (two small interfering RNAs and one lentiviral shRNA particles) was verified at the protein **(A)** and mRNA level **(B, C)** in multiple tumor cell lines. Figure **(A)** shows the representative immunoblot results with at least three biological replicates. Figure **(B, C)** shows SHCBP1 mRNA expression relative to GAPDH in the above cell lines. Data are expressed as mean ± SD (n=3) and analyzed by the unpaired Student’s t test, **p < 0.01, ***p < 0.001, ****p < 0.0001.


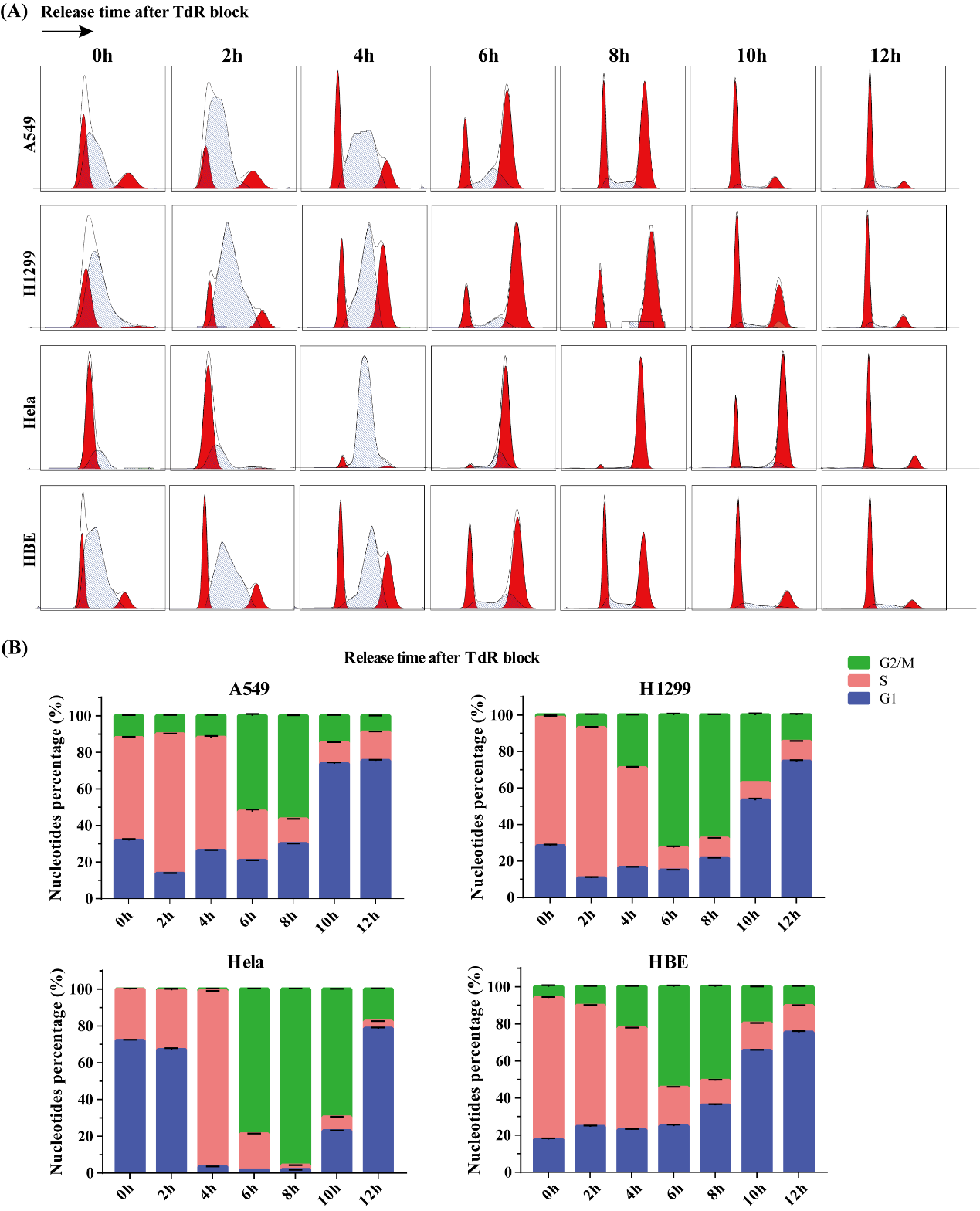


**Supplementary Figure 6. Representative DNA histograms (A) and statistical bar chart (B) obtained by flow cytometry of all cells at different time points after release from the TdR block.**


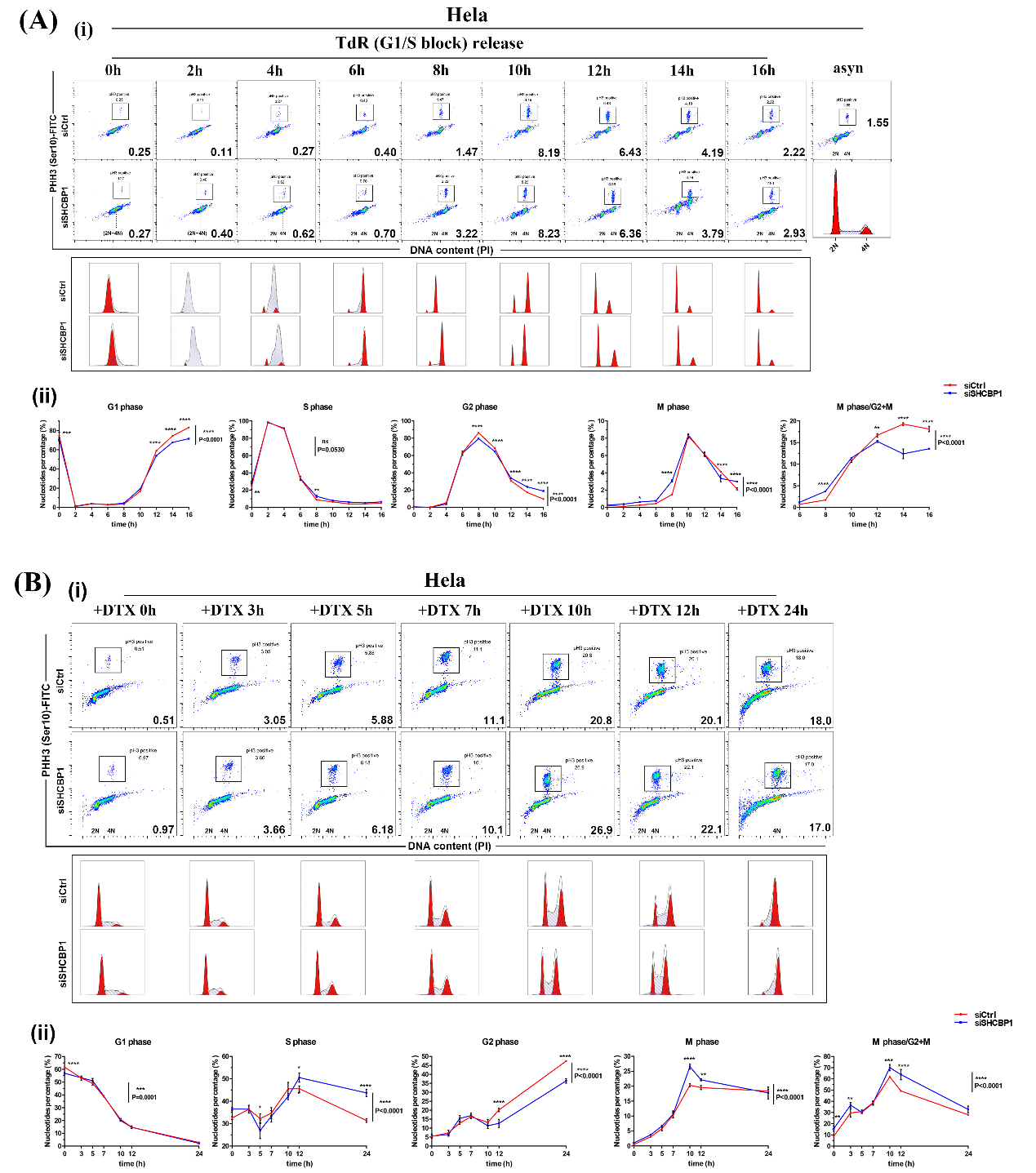


**Supplementary Figure 7. SHCBP1 knockdown slows tumor cell cycle but promotes premature mitotic entry in Hela cells**

**(A)** Cell cycle analysis of HeLa cells transfected with control or SHCBP1 siRNA at indicated time points after double thymidine (TdR) release. (i), flow cytometry plots of cells released at various time points and normally growing asynchronous (asyn) cells analyzed by using ModFit LT 5.0 and FlowJo software. Cells were stained with or without PHH3 (Ser10)-FITC antibody followed by propidium iodide (PI) staining. PHH3 (Ser10) positive cells indicating cells in M phase have been marked by small boxes on the dot plot. (ii), the line plot shows the change trend of cell proportion in each cell cycle phase (cell proportion of G1, S, G2 and M phase to the whole cell cycle, and the M phase to the G2+M [4N] phase) over the released time. Data are expressed as mean ± SD (n=3 independent experiments) and analyzed by the two-way ANOVA test (ns, not significant; *, P < 0.05; **, P < 0.01; ***, P < 0.001; ****, P < 0.0001).

**(B)** HeLa cells transfected with control or SHCBP1 siRNA were subjected to 0.1μM docetaxel (DTX) treatment for 0 h, 3 h, 5 h, 7 h, 10 h, 12 h and 24 h for cell cycle analysis, respectively. The legends of (i) and (ii) are similar to those in Figure A.


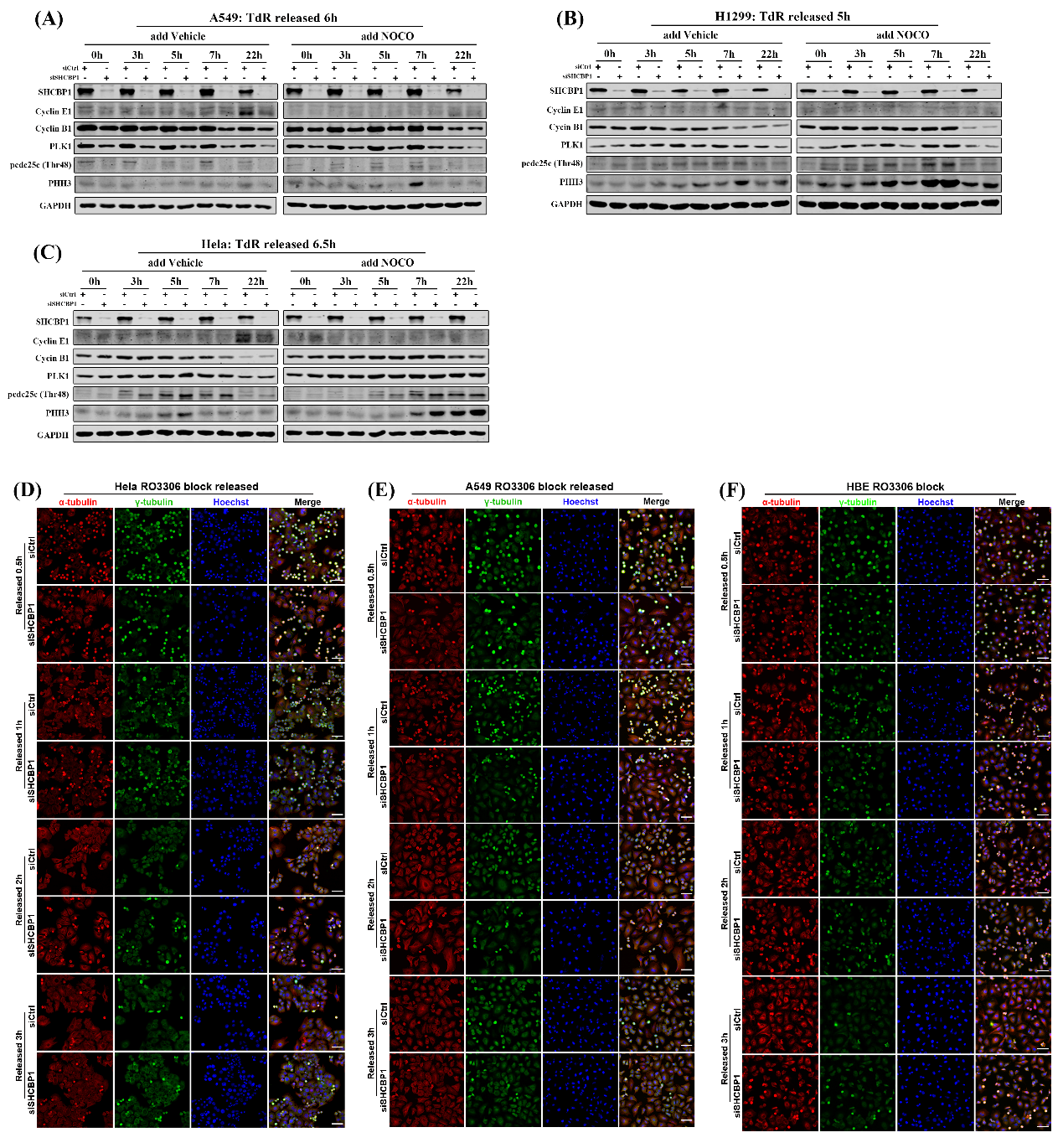
**Supplementary Figure 8. Effect of SHCBP1 knockdown on tumor cells entering and exiting M phase**

**(A-C)** A549, NCI-H1299 and HeLa cells transfected with control or SHCBP1 siRNA were synchronized in G2 phase by allowing progression for indicated time after double thymidine release and then exposed to nocodazole or vehicle for various times. Western blot images of SHCBP1, cyclin E1 (S-phase protein), cyclin B1, PLK1, p-cdc25c(Thr48) and PHH3 (Ser10) (four M-phase proteins) with GAPDH as internal reference at each time point are shown.

**(D-F)** HeLa, A549 and HBE cells transfected with control or SHCBP1 siRNA were released from RO3306 block (9μM) for 0.5 h, 1 h, 2 h and 3 h, respectively. Representative immunofluorescence images showing co-staining of α-tubulin (red), γ-tubulin (green) and Hoechst (blue) at each time point. The round cells with dense chromosomes are mitotic cells. Scale bars, 50μm.

**
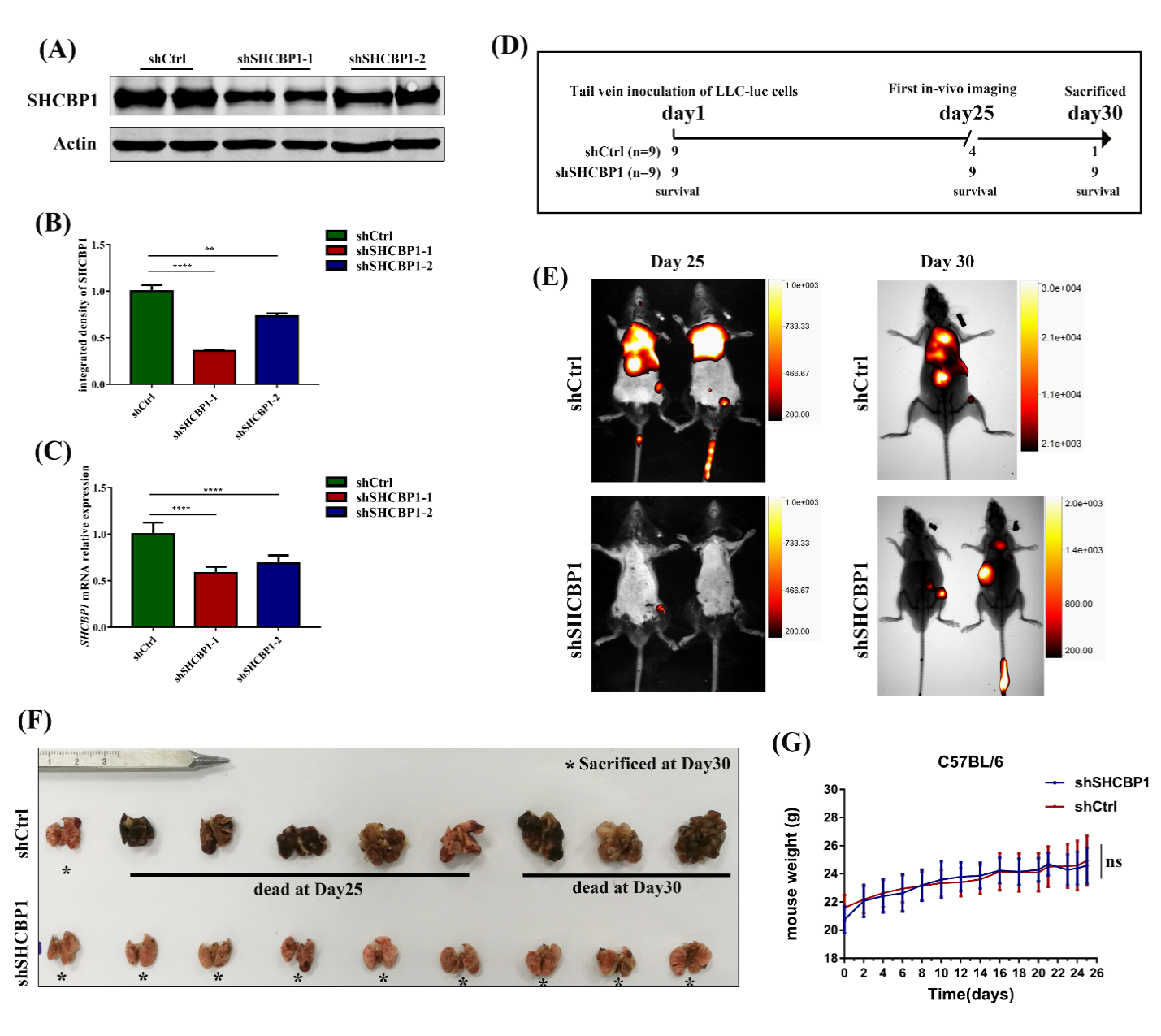
**

**Supplementary Figure 9. SHCBP1 knockdown inhibits tumor proliferation and metastasis in C57BL/6 mice**

**(A-C)** The knockdown efficiency of two shRNA lentiviruses of murine SHCBP1 (lentiviral shRNA1 and shRNA2 particles) was examined at the protein (**A, B**) and mRNA level **(C)** in Lewis lung carcinoma (LLC) cells. Representative western blot images **(A)** and the corresponding quantification of SHCBP1 with β-actin as internal reference **(B)** are shown. SHCBP1 mRNA expression relative to GAPDH was analyzed **(C)**. Data are expressed as mean ± SD (n=3) and analyzed by unpaired Student’s t test, **p < 0.01, ****p < 0.0001.

**(D)** Flow chart of lung adenocarcinoma metastasis model mediated by tail vein injection of 1ⅹ10^6^ stable luciferase-expressing LLC cells (LLC-LUC) in two groups of C57BL/6 mice (shCtrl and shCBP1 groups, n=9/group). In-vivo bioluminescence (BL) imaging was performed on days 25 and 30 after tumor inoculation, and all surviving mice were sacrificed after in-vivo imaging on day 30.

**(E)** Representative in-vivo BL images of tumor-bearing mice on days 25 and 30. The range of BL intensity of tumors in mice has been shown at right of each image (day 25: 200 to 1ⅹ10^3^; day 30: shCtrl group= 2.1ⅹ10^3^ to 3ⅹ10^4^, shSHCBP1 group=200 to 2ⅹ10^3^).

**(F, G)** Isolated mouse lungs **(F)** and the mouse weight curve **(G)** of two groups of tumor-bearing C57BL/6 mice (shCtrl group and shSHCBP1 group) are displayed. P value was determined by the two-way ANOVA test; ns, not significant.

**
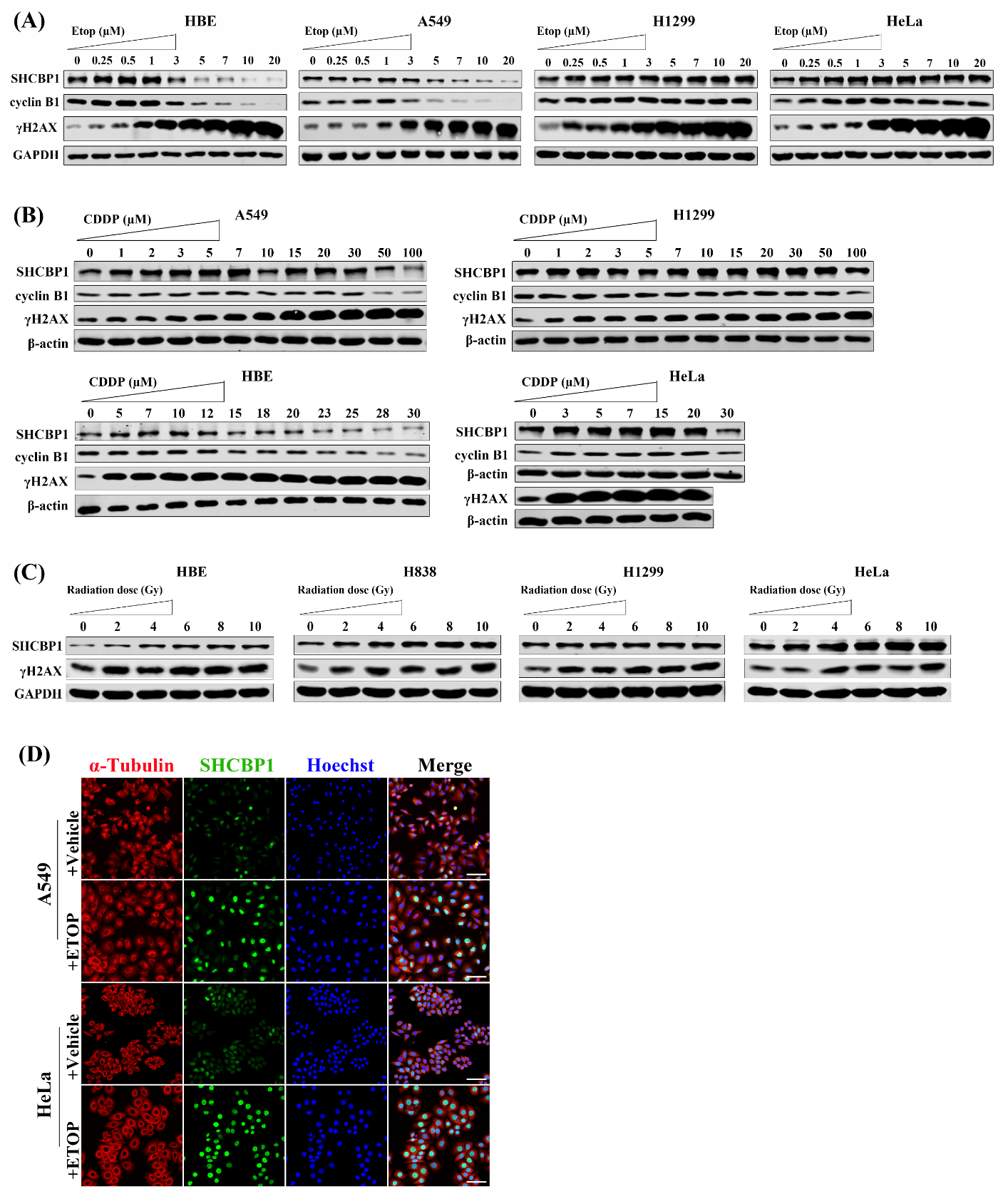
Supplementary Figure 10. Expression of SHCBP1 is elevated after treatment with DNA-damaging agents**

**(A-C)** HBE, A549, NCI-H1299 and HeLa cells were transfected with control or SHCBP1 siRNA for 24 h, followed by 24 h treatment with ETOP, CDDP or radiation at different concentrations or doses. Western blot images of SHCBP1, cyclin B1 and γH2AX with GAPDH or β action as internal reference are shown.

**(D)** Immunofluorescence images of SHCBP1 (green) and α-tubulin (red) co-staining in A549 and HeLa cells after treated with siCtrl or siSHCBP1 for 24 h followed by low-dose ETOP (1μM for A549 cells and 3μM for HeLa cells) or vehicle treatment for 24 h. Scale bars, 100μm.

**
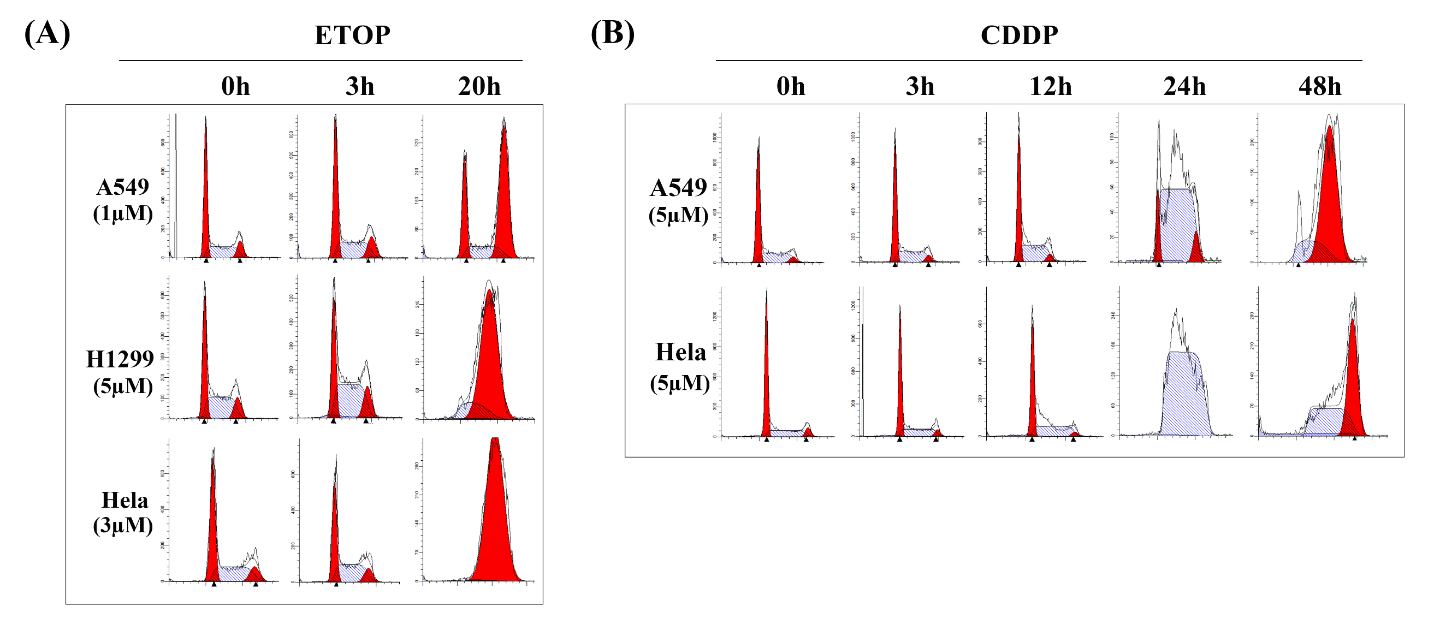
**

**Supplementary Figure 11. Cell cycle arrest after low-dose etoposide and cisplatin treatment**

Flow cytometry plots showing that cells were arrested in G2 phase after low-dose etoposide (ETOP) treatment **(A)** and in S phase after low-dose cisplatin (CDDP) treatment, but could be arrested in G2 phase after a longer time **(B)**.

**
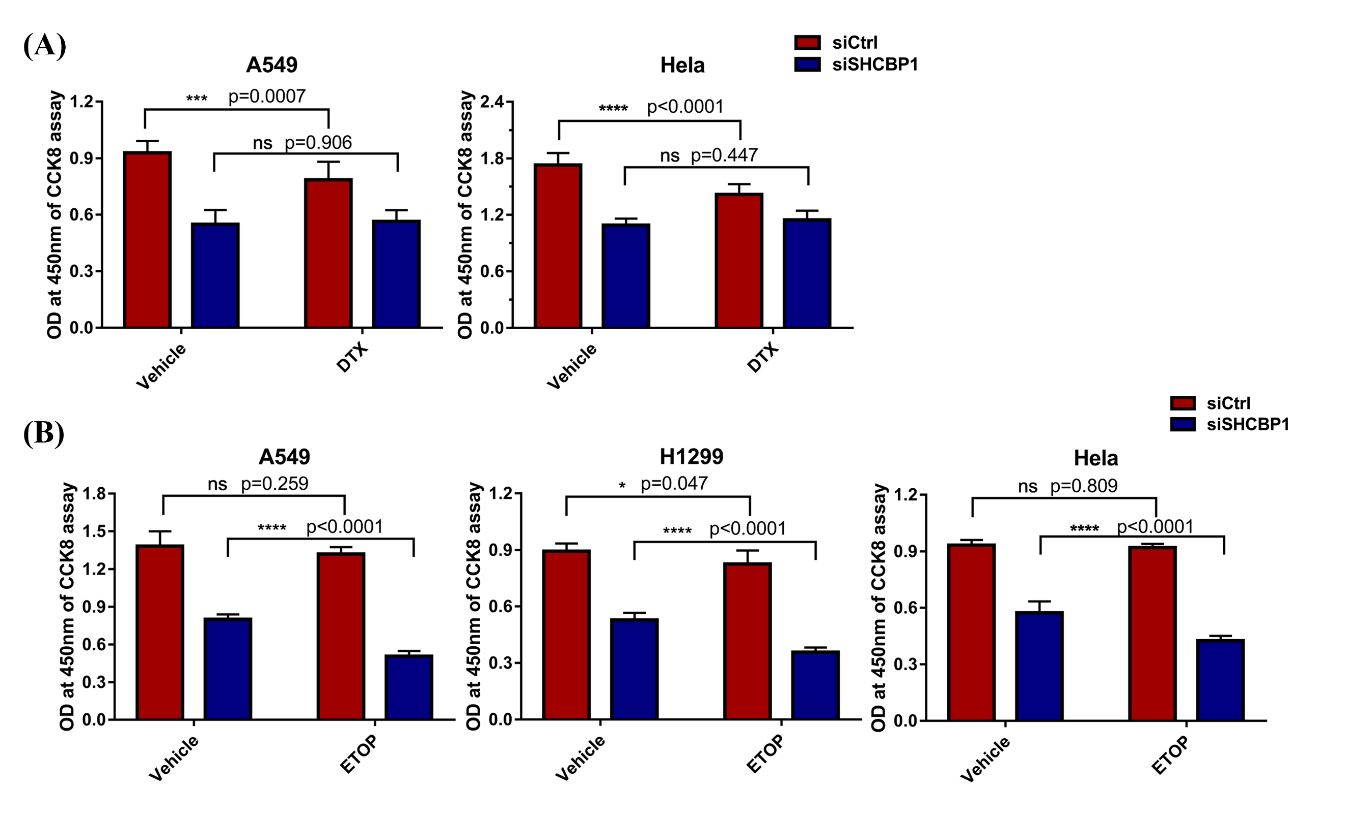
Supplementary Figure 12. CCK 8 assay in tumor cells after SHCBP1 knockdown combined with docetaxel (A) or etoposide exposure (B)**

Cells were transfected with control or SHCBP1 siRNA for 24 h, followed by 0.1μM docetaxel (DTX) or low-dose etoposide (ETOP, same as described previously) treatment for 24 h. Data are shown as mean ± SD (n≥5). P values were determined by two-way ANOVA with Sidak’s multiple comparison test; ns=not significant; *p < 0.05, ***p < 0.001, ****p < 0.0001.


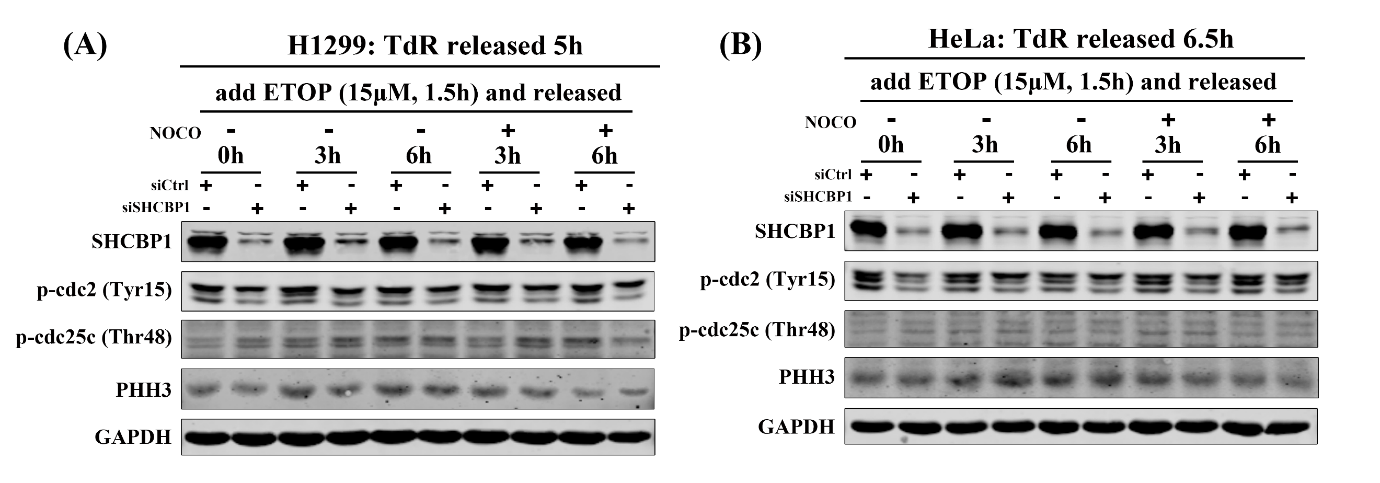


**Supplementary Figure 13. Tumor cells transfected with siCtrl or siSHCBP1 siRNA did not enter M phase within a short time after DNA damage in G2 phase**

NCI-H1299 **(A)** and HeLa cells **(B)** transfected with siCtrl or siSHCBP1 siRNA were synchronized in G2 phase by allowing progression for indicated time after double thymidine release and then exposed to 15 μM etoposide for 1.5h, then washed out into fresh medium with or without nocodazole (NOCO) for 3 or 6 hours. Western blot analysis of the SHCBP1, p-cdc2(Tyr15), p-cdc25c(Thr48) and PHH3 was performed in these cells with GAPDH as internal reference.


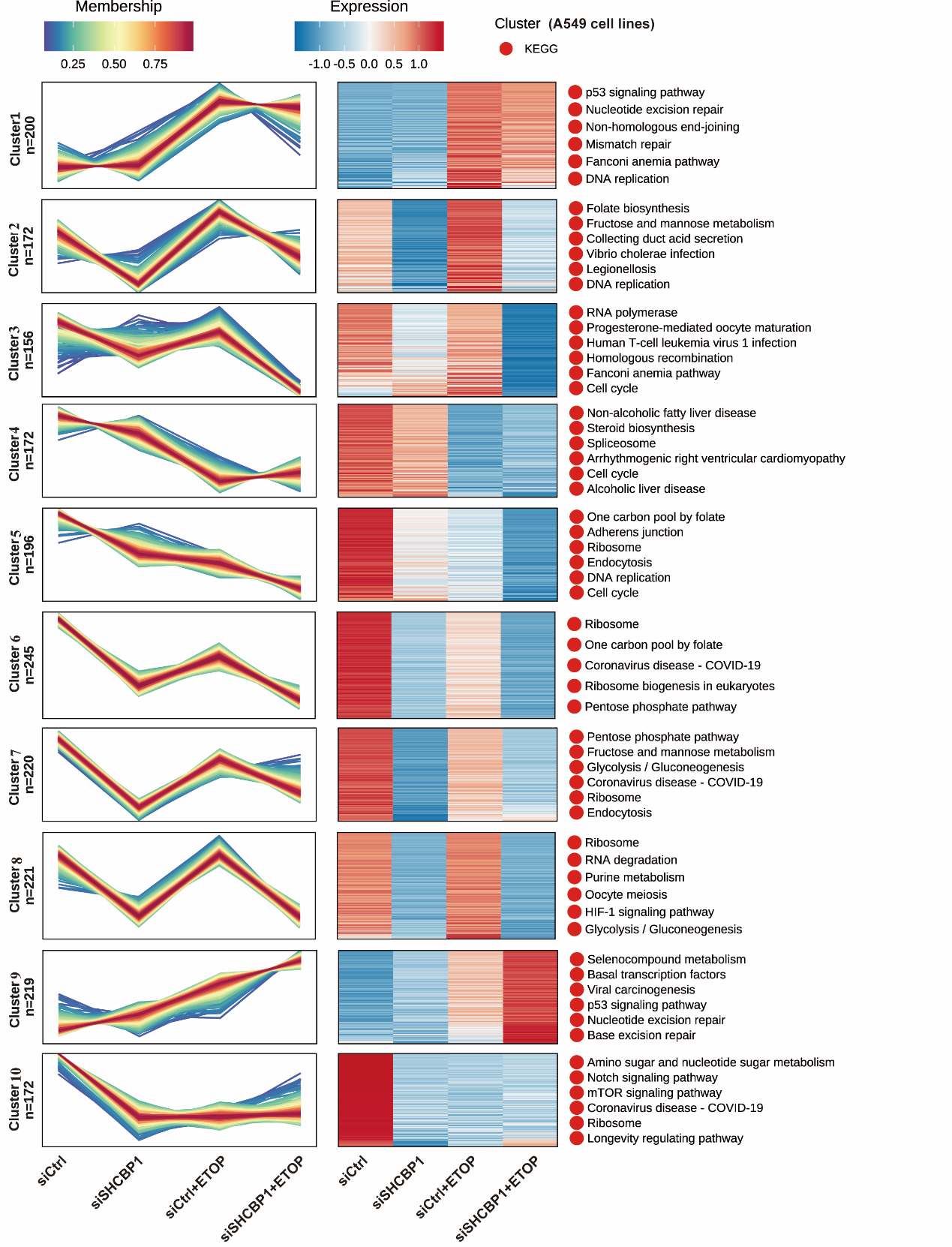


**Supplementary Figure 14. proteomic analysis of A549 cells after combination treatment**

A549 cells were transfected with control or SHCBP1 siRNA for 24 h and then treated with 3μM etoposide or corresponding vehicle for 24 h. The cells were collected for proteomic analysis to identify differentially expressed proteins (DEPs) between siCtrl, siSHCBP1, siCtrl+ETOP, siSHCBP1+ETOP. DEPs clustered by their expression pattern across the different treatment group were achieved by applying fuzzy c-means clustering analysis using the R package Mfuzz.


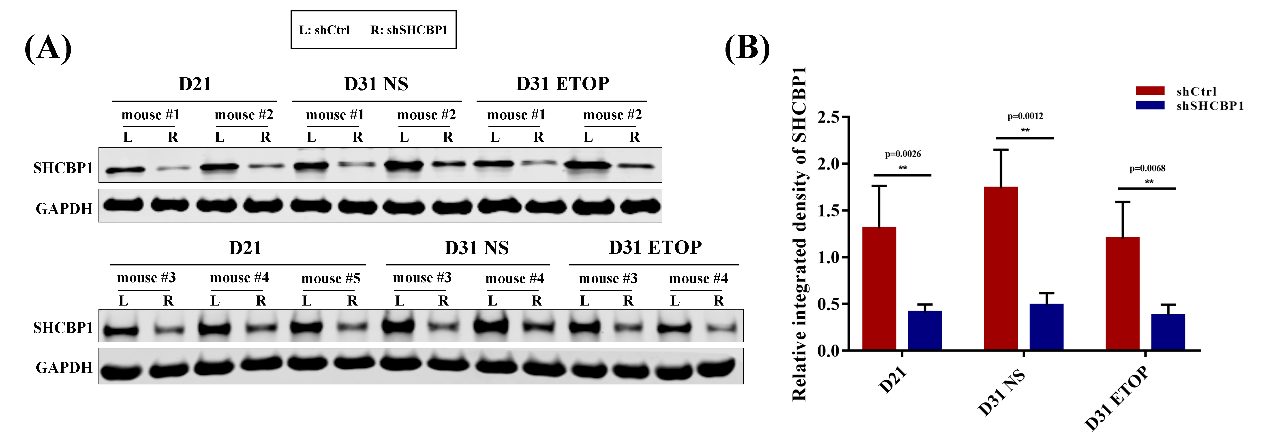


**Supplementary Figure 15. The expression of SHCBP1 in mouse subcutaneous tumor tissues throughout the modeling period**

1. Western blot analysis of SHCBP1 in the shCtrl and shSHCBP1 subcutaneous tumor tissues on the 21st and 31st day in NS group and ETOP group respectively.
2. Statistical analysis of western blot images in (A). Data are shown as mean ± SD (n=5 or 4 each group). P values were determined by unpaired Student’s t test. **, P < 0.01.
