# Supplementary material for: The dynamic role of nucleoprotein SHCBP1 in the cancer cell cycle and its potential as a synergistic target for DNA-damaging agents in cancer therapy
Source: Cell Commun Signal. 2024 Feb 16;22:131. doi: 10.1186/s12964-024-01513-0 (PMC10874017; doi:10.1186/s12964-024-01513-0)
Supplement: Supplementary file 3 — Additional file 3: Supplementary video 1. H1299 cells were transfected with control or SHCBP1 siRNA for 24 hours and then exposed to 5 μM ETOP for real-time recording. The rounded cells in the real-time video indicate mitotic cells. Supplementary video 2. Hela cells were transfected with control or SHCBP1 siRNA for 24 hours and then exposed to 3 μM ETOP for real-time recording. The rounded cells in the real-time video indicate mitotic cells. [file 12964_2024_1513_MOESM3_ESM.zip › Supplementary video legends.docx]

**Supplementary video legends**

**Supplementary video 1. H1299 cells were transfected with control or SHCBP1 siRNA for 24 hours and then exposed to 5 μM ETOP for real-time recording**

The rounded cells in the real-time video indicate mitotic cells

**Supplementary video 2. Hela cells were transfected with control or SHCBP1 siRNA for 24 hours and then exposed to 3 μM ETOP for real-time recording**

The rounded cells in the real-time video indicate mitotic cells
